# Causal effects for genetic variants of osteoprotegerin on the risk of acute myocardial infarction and coronary heart disease: A two-sample Mendelian randomization study

**DOI:** 10.3389/fcvm.2023.1041231

**Published:** 2023-03-07

**Authors:** Peng Chao, Xueqin Zhang, Lei Zhang, Xinyue Cui, Shanshan Wang, Yining Yang

**Affiliations:** ^1^Department of Cardiology, People's Hospital of Xinjiang Uygur Autonomous Region, Xinjiang, China; ^2^Department of Nephropathy, People's Hospital of Xinjiang Uygur Autonomous Region, Xinjiang, China; ^3^Department of Endocrine, People's Hospital of Xinjiang Uygur Autonomous Region, Xinjiang, China

**Keywords:** two-sample Mendelian randomization, osteoprotegerin, acute myocardial infarction, coronary heart disease, casual effect

## Abstract

Although since the 1980s, the mortality of coronary heart disease(CHD) has obviously decreased due to the rise of coronary intervention, the mortality and disability of CHD were still high in some countries. Etiological studies of acute myocardial infarction(AMI) and CHD were extremely important. In this study, we used two-sample Mendelian randomization(TSMR) method to collect GWAS statistics of osteoprotegerin (OPG), AMI and CHD to reveal the causal relationship between OPG and these two diseases. In total, we identified 7 genetic variants associated with AMI and 7 genetic variants associated with CHD that were not found to be in linkage disequilibrium(LD; *r*^2^ < 0.001). Evidence of a positive effect of an OPG genetic susceptibility on AMI was discovered(IVW OR = 0.877; 95% CI = 0.787–0.977; *p* = 0.017; 7 SNPs) and CHD (IVW OR = 0.892; 95% CI = 0.803–0.991; *p* = 0.033; 7 SNPs). After removing the influence of rs1385492, we found that there was a correlation between OPG and AMI/CHD (AMI: weighted median OR = 0.818;95% CI = 0.724–0.950; *p* = 0.001; 6SNPs;CHD: weighted median OR = 0.842; 95% CI = 0.755–0.938; *p* = 1.893 × 10^−3^; 6SNPs). The findings of our study indicated that OPG had a tight genetic causation association with MI or CHD. This genetic causal relationship presented us with fresh ideas for the etiology of AMI and CHD, which is an area of research that will continue in the future.

## Introduction

Although coronary intervention and the management of risk factors such as arterial hypertension, hyperlipidemia, diabetes, and smoking have resulted in a marked decline in CHD mortality rates since the 1980s, CHD remains a leading cause of death and disability in many parts of the world (1). In 2019, there were 56.5 million deaths worldwide, of which 32.9% (18.6 million deaths) were caused by cardiovascular diseases (2). As part of the Sustainable Development Goals (SDGs), the United Nations aims to reduce premature mortality due to noninfectious chronic diseases (NCDs) by one third. Reducing in cardiovascular diseases, especially ischemic heart disease (IHD), might partially achieve this goal (2–4). As a result, it is necessary to keep an eye on the risk factor of CHD. Pathologically, AMI is considered as irreversible necrosis of myocardial cells caused by acute ischemia. (5). Every year, more than 8 million Americans are hospitalized for signs and symptoms that suggest AMI. About 1,700,000 people were finally diagnosed with myocardial infarction(MI) (6). Thus, etiological studies of AMI and CHD are extremely important.

MI patients may benefit from the use of bone-related proteins for early risk stratification and prognosis evaluation. In patients with AMI, OPG levels are correlated with the complexity of coronary artery disease(CAD) (7, 8). Healthy subjects with low or high coronary artery calcification cannot be distinguished by using OPG. A single OPG measurement is limited to the diagnosis of angina pectoris (AP) in patients with suspected CAD (9, 10). OPG is identified as a new biomarker of cardiovascular mortality and clinical events in patients with AMI complicated with heart failure. These findings are consistent with the hypothesis that there may be a connection between bone homeostasis mediators and cardiovascular diseases (10). However, the causal relationship between OPG and AMI or CHD has not been systematically tested due to the existence of potential deviations such as confounding factors or reverse causality, and the causal relationship between OPG and AMI or CHD is still unclear.

In the traditional epidemiology, observational research is used to explore the causes of diseases, but the whole exploration process lacks the content of causal inference, which is often considered unreliable. Randomized controlled study (RCT) is considered to clearly explain factors that contribute to disease outcomes and their causal relationships. However, due to its ethical limitation and its colossal human resources and material resources, RCT research has not been widely implemented in the clinics. In recent years, Mendelian randomization (MR) design has been considered as one of the best ways to make up for RCT. To solve the above dilemma, GWAS database tool variables and genetic variation can be taken as exposure factor tool variables (11).

Analysis of TSMR is one of the most commonly used methods in MR has the following advantages. First of all, with the publication of a large number of GWAS, we can obtain a large number of GWAS data. Secondly, a two-sample study would have included subjects from both cohorts by using previous observational study cohorts, which can significantly expand the sample size and improve detection effectiveness. Finally, with the increasing amount of GWAS data, we can obtain a huge number of tool variables, which also increases the genetic explanation of the causal relationship between the tool variables related to exposure factors and the outcome, making the obtained results more reliable. In this study, we used the TSMR method to collect GWAS statistics of OPG, AMI or CHD to reveal the relationship between OPG and these two diseases.

## Methods

### Study design

We used the TSMR method to investigate the causal relationship between OPG and cardiovascular diseases (including AMI and CHD). This study adopted the published summary data in the GWAS database, so it had no use for ethical approval. It is important to note that this study has not been preregistered and should therefore be considered exploratory in nature.

**Figure 1:**
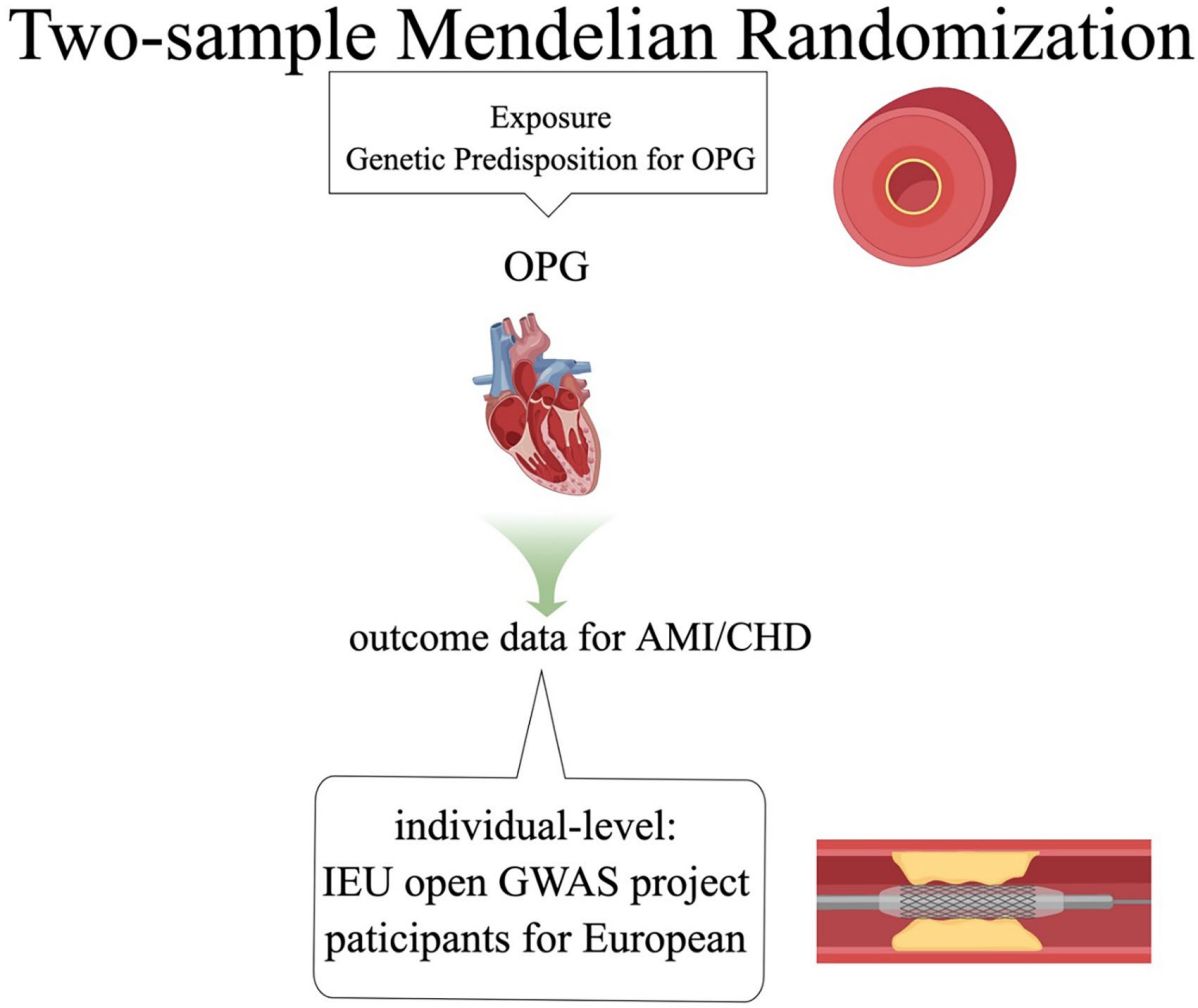
Graphical abstract.

**Figure 2:**
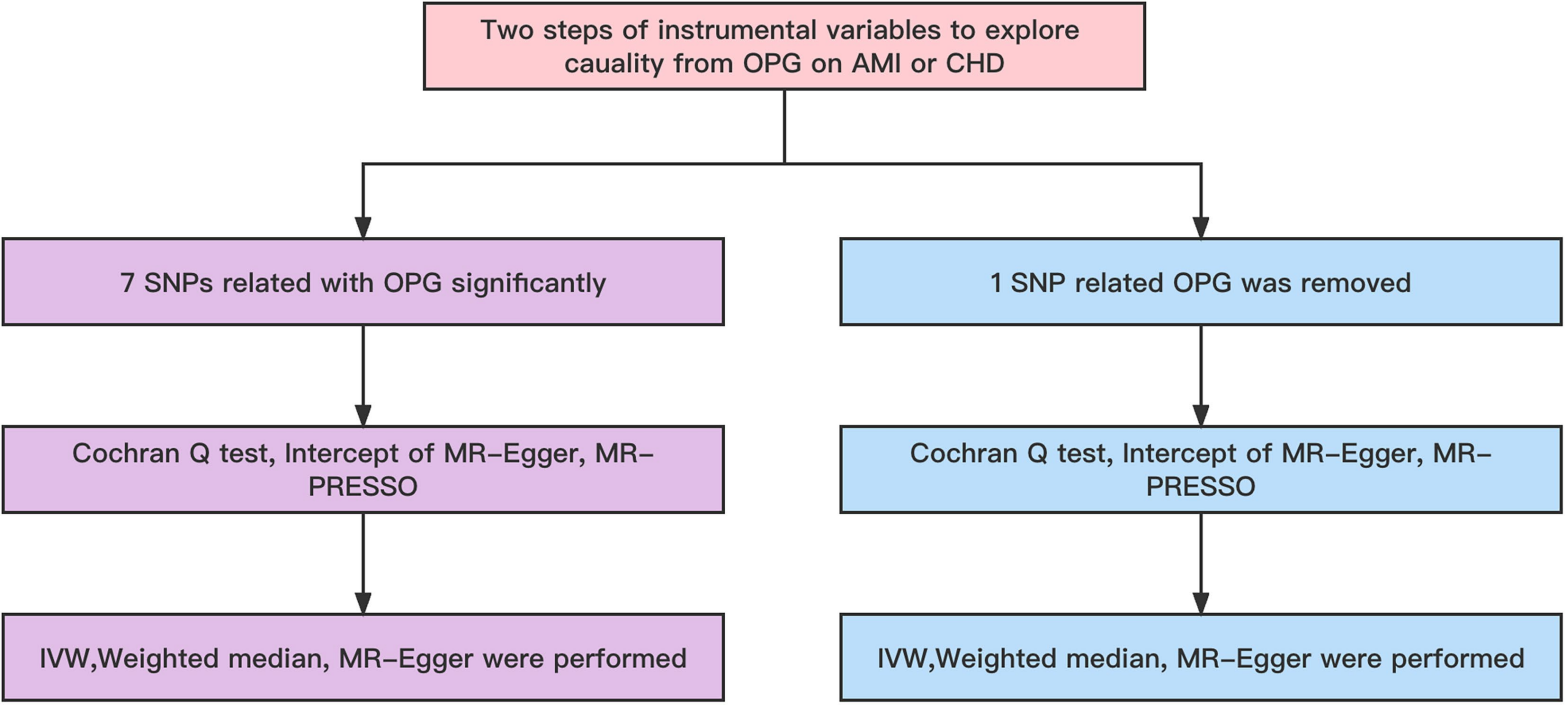
Workflow of MR. IVW, Inverse variance weighted; MR Pleiotropy RESidual Sum and Outlier; SNP, single-nucleotide polymorphisms.

Our basic design is shown in Figures 1, 2. (1) The instrumental variables (IVs) have nothing to do with confounding factors; (2) The IVs have something to do with the exposure factor. (3) IVs are not directly related to the ending variable, but can only be related to the outcome variable through the exposure factor. The study was conducted using the two-sample MR package (version 0.5.4) and the ‘Mendelian Randomization’ package (version 0.5.1) of the R program (version 4.0.0).

CHD was defined as a compound definition including MI, acute coronary syndrome, chronic stable angina, or coronary stenosis >50%, and AMI included in the original GWAS database was all myocardial ischemia-related myocardial infarction, and non-ischemic myocardial infarction was not included in the study.

### Data sets

The summary-level data of OPG was provided by the GWAS summary statistics for Olink CVD-I proteins were collected from 13 European ancestry populations, which consists of 21,758 patients with OPG and 13,138,400 SNPs. All patients were from the European population (12). Summary data of AMI came from Coronary ARtery DIsease Genome wide Replication and Meta-analysis (CARDIoGRAM) plus The Coronary Artery Disease (C4D) Genetics(CARDIoGRAMplusC4D) and CHD also came from CARDIoGRAMplusC4D (13). The MI data set included 43,676 patients with MI and 128,199 controls, all of whom are from Europe (12). The CHD data set included 60,801 patients with CHD and 123,504 controls (13). According to the queuing information reported in the initial GWAS analysis, our investigation identified no sample overlap between OPG and AMI or CHD.

### IVs selection and validation

IVs must be closely related to OPG. In order to ensure the relationship between OPG and IVs, we chose *p* < 5×10^−8^ as the IVs in the GWAS database. Furthermore, PLINK 1.9015 method was applied to remove disequilibrium in the linkage effect of IVs to ensure the independence of the selected IVs. We should guarantee that *r*^2^ < 0.001 in IVs, and those who do not fulfill the criteria should be eliminated. *F* value was used to assess the IVs’ capacity to predict exposure.

The chosen IVs must be independent of AMI and CHD, as well as several confounding variables. Besides, it needed to be closely related to OPG. To begin, we used the aforementioned criteria to choose just the IVs that would be most helpful. (14). Secondly, MR-egger was used for horizontal pleiotropy test (15). Subsequently, palindromic SNPs which were defined as having minor allele frequencies greater than 0.3 were removed to make sure the effect of SNP on plasma OPG corresponded to alleles with the same genotype due to its effect on CHD or AMI. Then, the GWAS catalog of^1^ was used to check the connection of the chosen IVs with adjusted potential confounders. Finally, we produced F statistics utilizing the online application^2^ to discover the correlation between the specified IVs and OPG.

### Statistical analysis

In the present study, the follow-up sensitivity was evaluated using the weighted median, simple median, maximum likelihood, and penalized weighted median methods. The weighted median, simple median, maximum likelihood, and penalized weighted median methods are more robust than the inverse-variance weighted (IVW) for individual genes with highly outlying causal estimations and produce a consistent estimate of the causative influence when valid IVs surpass 50%.

Firstly, our main analysis method was IVW in order to investigate causal relationships between exposure factors and outcomes. To determine whether the MR hypothesis was violated, a sensitivity analysis was conducted. We used Cochran Q-test and I^2^ statistics to detect the heterogeneity of the IVW model (16). In the Cochran Q test, when I ^2^ > 25% and *p* < 0.05, potential heterogeneity was regarded as existing. Excessive heterogeneity indicated that modeling assumptions were violated or invalid instruments were included, leading to horizontal pleiotropy (17). Due to its inherent robustness to heterogeneous outliers, weighted median models were recommended for causal inference in this case. They offered a slightly lower estimation accuracy, but provided a somewhat higher estimation accuracy (18). By using the MR-Egger intercept, a directional pleiotropy was detected. In order to determine whether any single SNP was responsible for the combined IVW estimate, we performed a leave-one-out analysis. Observed causal estimates were filtered using Steiger filtering to determine whether reverse causality affected the observed association (19). TSMR analysis was also repeated, but rs1385492 related to OPG was removed for genome-wide significance analysis (*p* < 5 × 10–8), and leave-one-out analysis was performed to assess causality. As a next step, we checked in the GWAS catalog whether any of the remaining 6 SNPs have a secondary phenotype associated with cardiovascular disease. MR-PRESSO was performed prior to MR analysis in order to identify and exclude any SNPs that might be pleiotropic.

## Results

### Genetic variants selection and validation

Overall, We obtained 7 AMI genetic variants and 7 LD-independent CHD genetic variants (*r*^2^ < 0.001). These genetic variants reached genome-wide significance in the dataset of genetic variants of OPG (*p* < 5*10–8). There were several SNPs that were not found directly in the CHD or AMI datasets, however. Table 1 showed all independent genetic variants analyzed through the TSMR approach. Consequently, we calculated the exposure from MR-Egger regression using the intercept term (Table 1) and found no horizontal pleiotropic pathway. F statistics were analyzed to determine the relationship strength between genetic variants and exposure. Statistical values of F over 10 were considered to be strong enough to eliminate all biases in causal genetic variant estimation. The F statistic value of the gene variants we selected was 1,120 for CHD and 1,073 for AMI, which is enough to alleviate any bias in the assessment of causal genetic variants.

**Table 1:** Genome-wide significant single nucleotide polymorphisms (SNPs) for OPG levels and their association with AMI and CHD.

SNP	OPG					AMI			CHD		
	E/o allele	Eaf	Beta	Se	*P*	Beta	Se	*P*	Beta	Se	*P*
rs114165349	C/G	0.038	−0.240	0.029	2.69 × 10^−16^	0.022	0.033	0.509	0.032	0.030	0.286
rs1385492	G/A	0.443	0.175	0.009	6.07 × 10^−76^	−0.003	0.010	0.783	0.000	0.009	0.959
rs17600346	C/T	0.038	0.175	0.028	3.01 × 10^−10^	−0.057	0.028	0.043	−0.051	0.025	0.047
rs2515001	T/C	0.144	−0.082	0.013	4.35 × 10^−10^	0.001	0.015	0.912	0.003	0.013	0.804
rs28929474	T/C	0.023	0.285	0.037	9.91 × 10^−15^	−0.148	0.052	0.004	−0.146	0.045	0.001
rs3761472	G/A	0.184	0.0700	0.012	2.95 × 10^−09^	−0.022	0.013	0.090	−0.017	0.011	0.145
rs704	A/G	0.473	−0.147	0.009	1.55 × 10^−55^	0.029	0.010	0.005	0.024	0.009	0.008

Eaf: allele frequency; E/O allele: effect allele/other allele; SE: standard error.

### Analyzed by TSMR and sensitivity analysis

**Figure 3:**
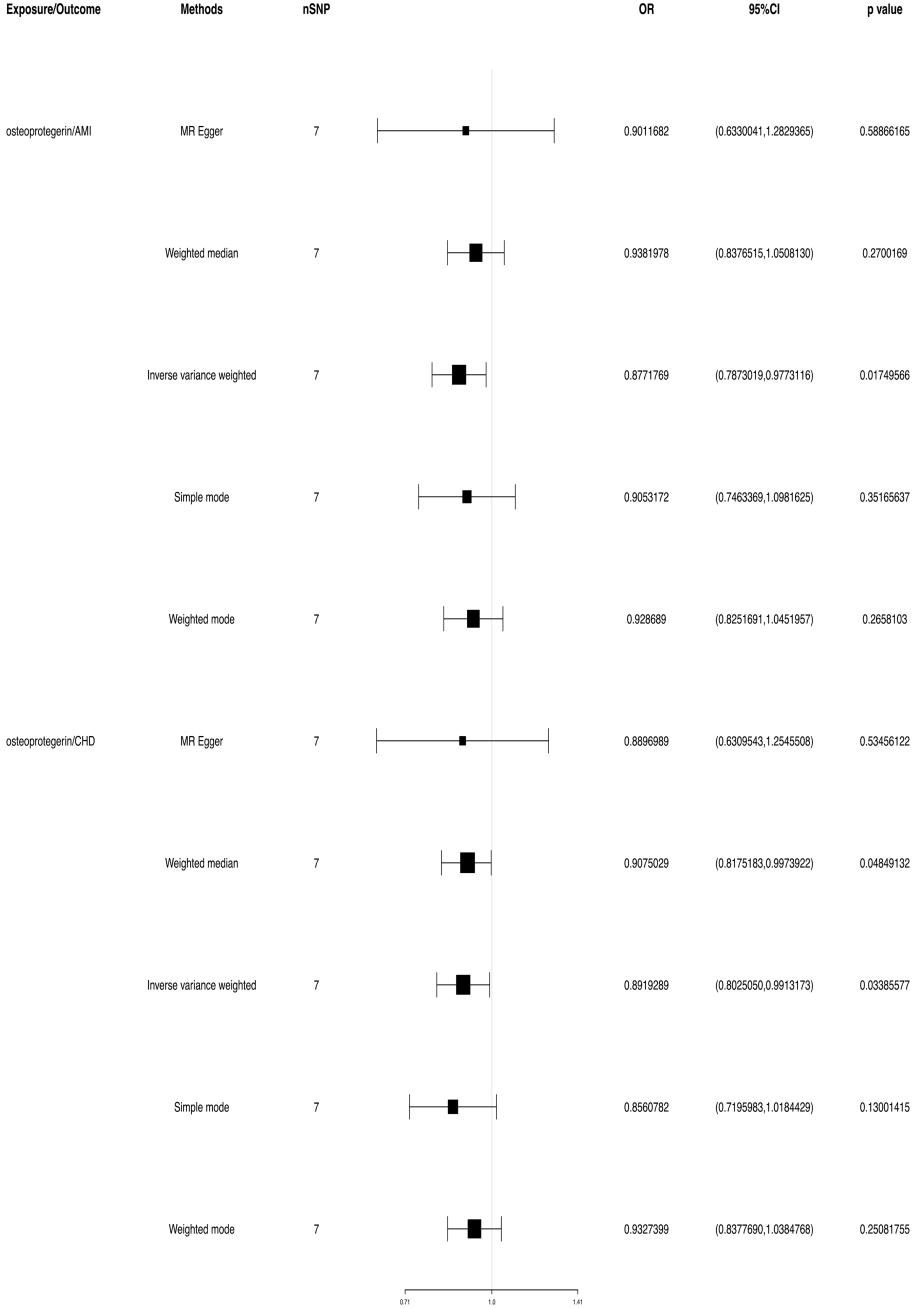
TSMR of plasma OPG and risk of AMI and CHD. We used the genome-wide association study (GWAS) of increasing plasma OPG level unit to summarize the statistics. The result was normalized to increase exposure by one unit. IVW, Inverse variance weighted; MR Pleiotropy RESidual Sum and Outlier; SNP, single-nucleotide polymorphisms.

**Figure 4:**
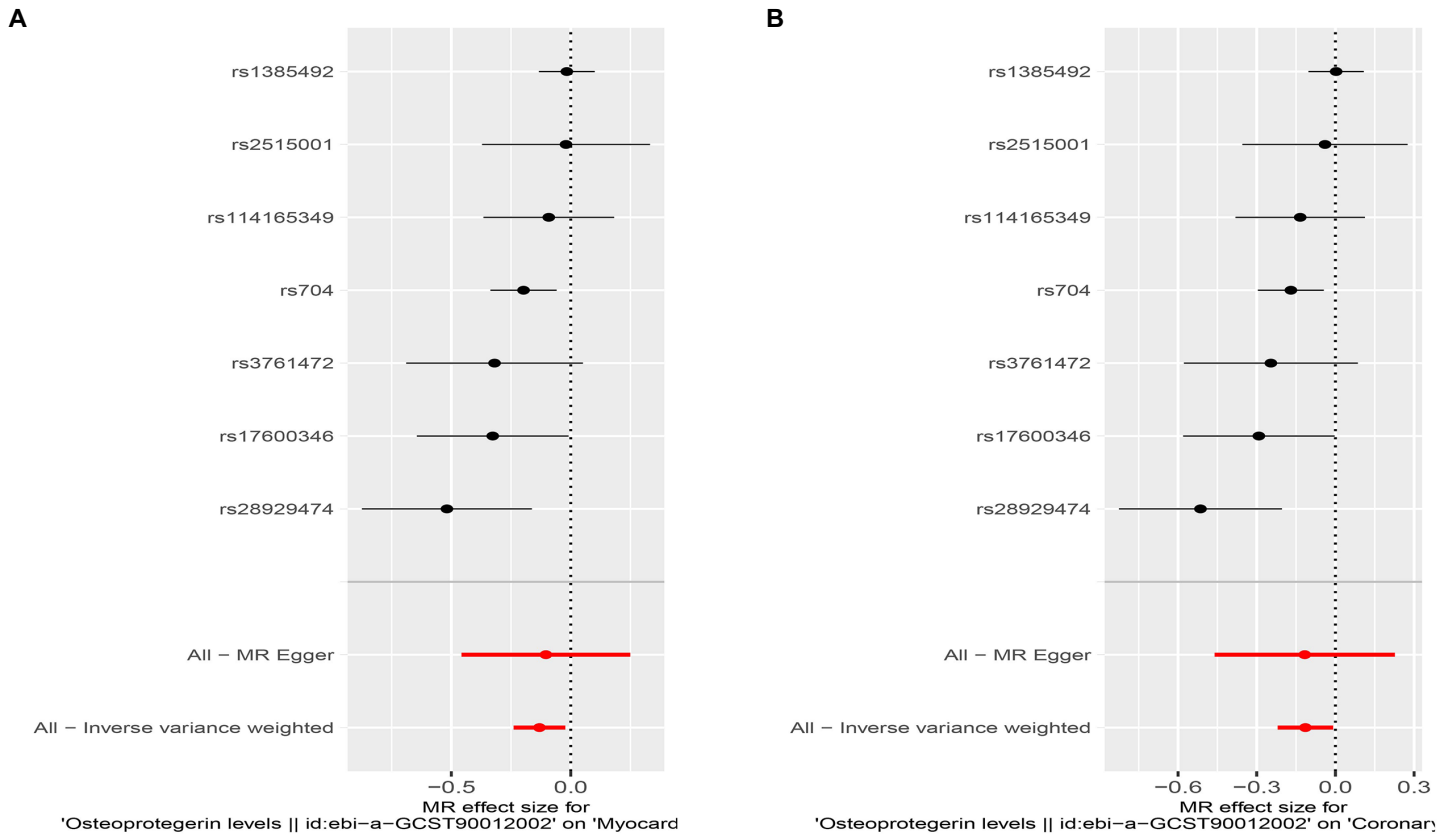
Results of the SNP analyses for the SNP effect of plasma OPG level on outcomes. **(A)** AMI **(B)** CHD.

**Figure 5:**
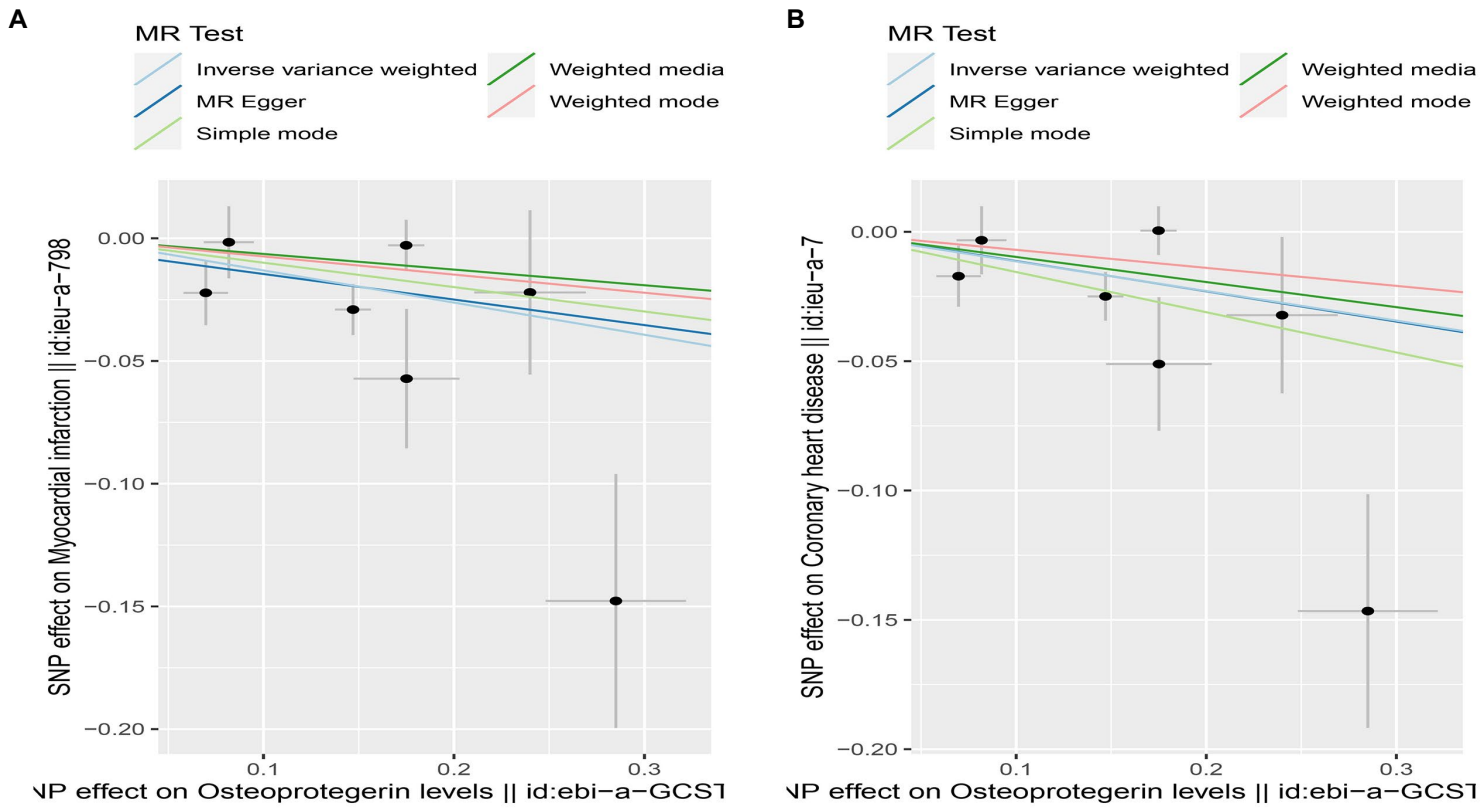
Scatterplot of MR estimates of genetic risk of OPG on AMI and CHD. Scatter-plot of genetic effects on OPG versus their effects on AMI **(A)** and CHD **(B)** with corresponding standard errors denoted by horizontal and vertical lines. The slope of each line corresponds to the estimated MR effect from different methods.

**Figure 6:**
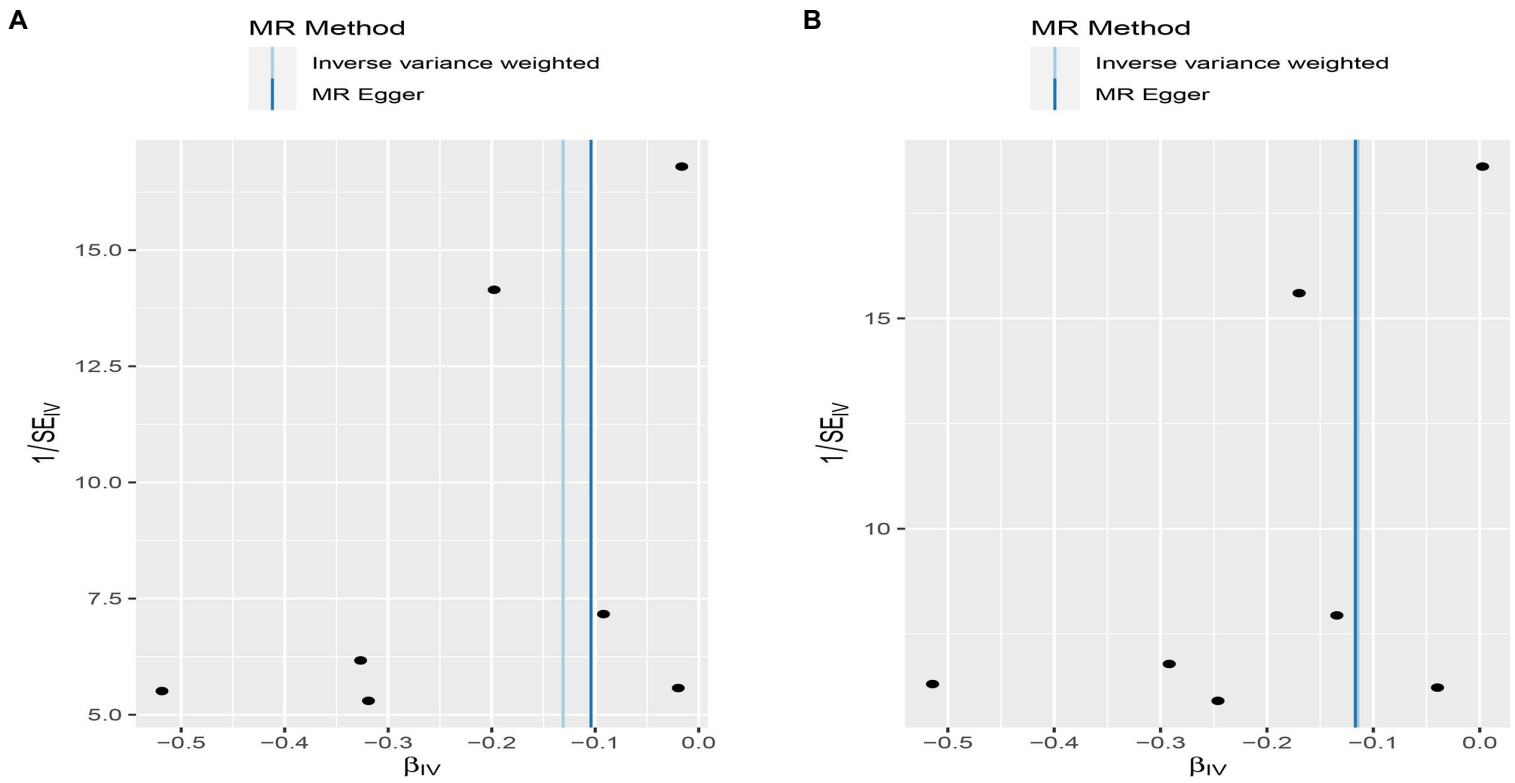
Funnel plot MR estimates of genetic risk of OPG on AMI and CHD. The funnel plot of genetic effect of OPG relative to its effect on AMI **(A)** and CHD **(B)**, and the distribution of corresponding points reflects the heterogeneity.

**Figure 7:**
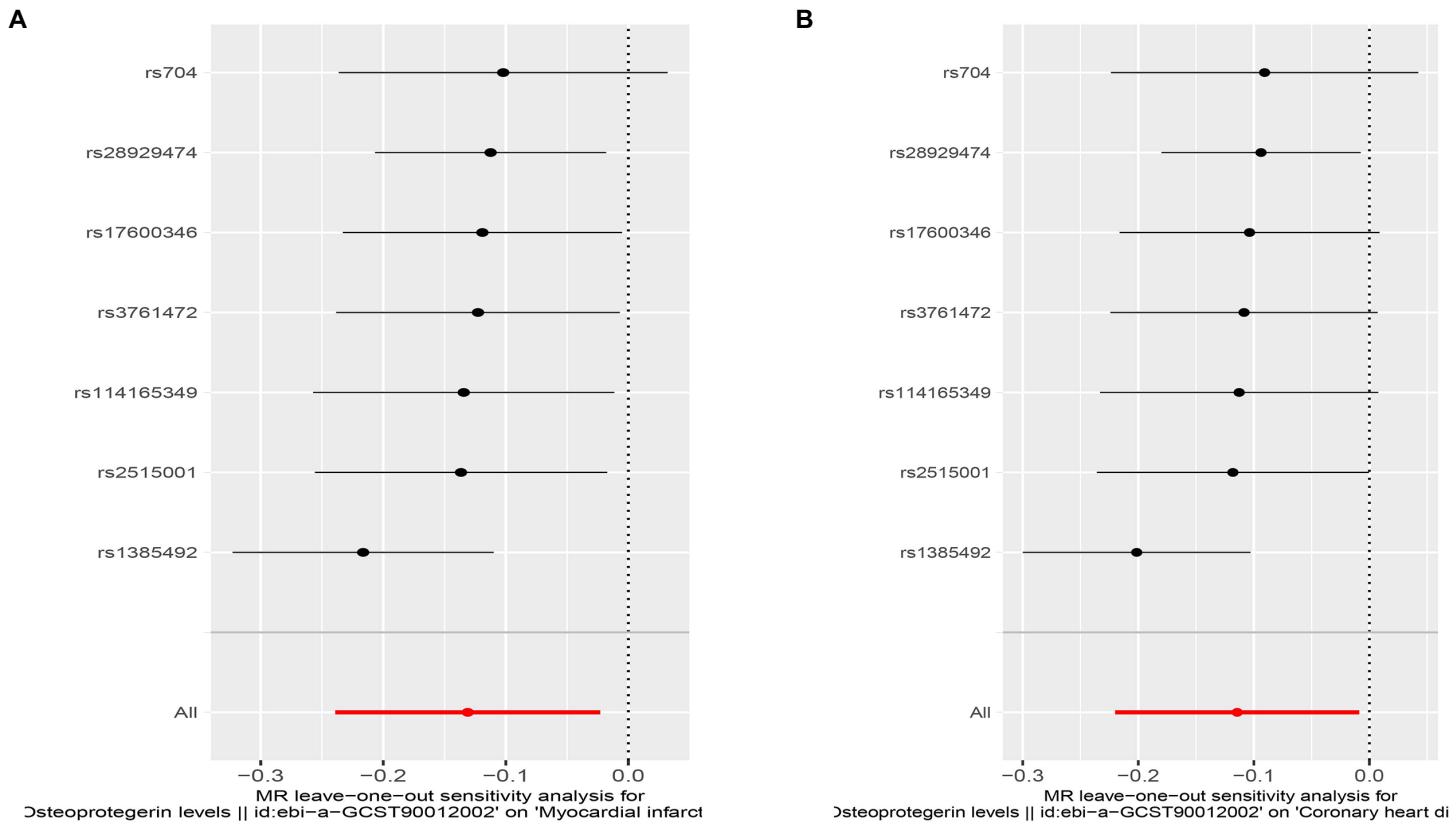
Sensitivity analyses using the leave-one-out approach for the association of plasma OPG level with outcomes. **(A)** AMI; **(B)** CHD.

We found evidence that OPG genetic predisposition is beneficial to AMI (IVW OR = 0.877; 95% CI = 0.787–0.977; *p* = 0.017; 7 SNPs) and CHD (IVW OR = 0.892; 95% CI = 0.803–0.991; *p* = 0.033; 7 SNPs). There was wide consistency among different models of MR in terms of causal estimates (Figures 3–5). Using Cochran’s Q-test, the IVW model appears to have no heterogeneity. Based on the MR-Egger intercept, there was no evidence of directional pleiotropy. Steiger filtering did not detect SNPs in the genome associated with reverse causation, and the association’s causal direction was reliable (Table 2; Figure 6). Additionally, we found that pooled IVW estimations were independent of any single SNP according to the leave-one-out analysis (Figure 7).

**Table 2:** Sensitivity analyses with complementary methods.

Exposure	outcome	Directional pleiotropy		Cochran Q-test		Steiger *P*
		intercepts	value of *p*	Q-statistic	*p*	
OPG	AMI	−0.004	0.879	12.067	0.060	2.89*10^−146^
OPG	CHD	0.0003	0.988	14.159	0.058	1.73*10^−147^

AMI: acute myocardial infarction; CHD: Coronary Heart Disease.

**Figure 8:**
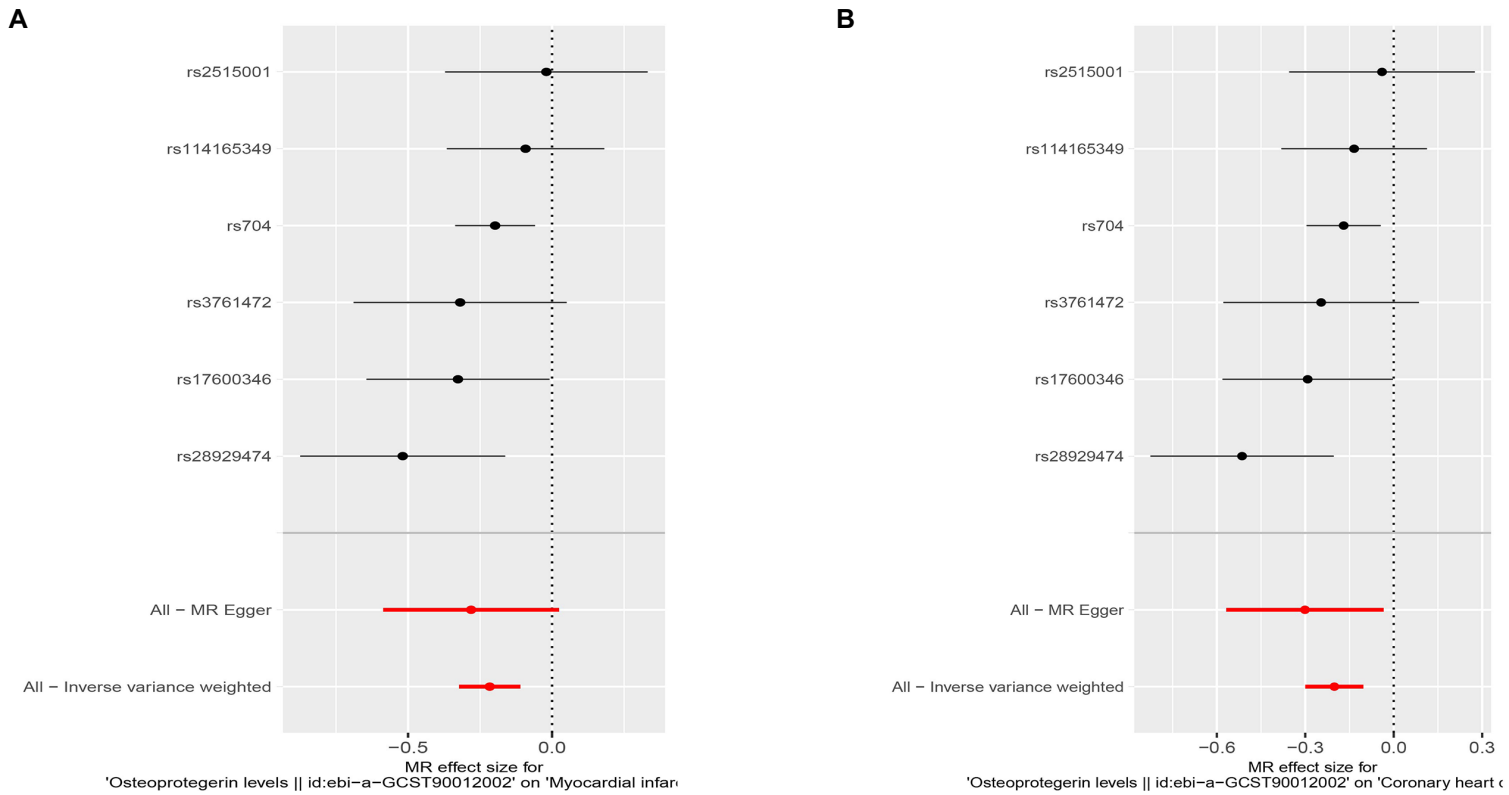
Results of the SNP analyses for the SNP effect of plasma OPG level on outcomes after removing rs1385492. **(A)** AMI **(B)** CHD.

**Figure 9:**
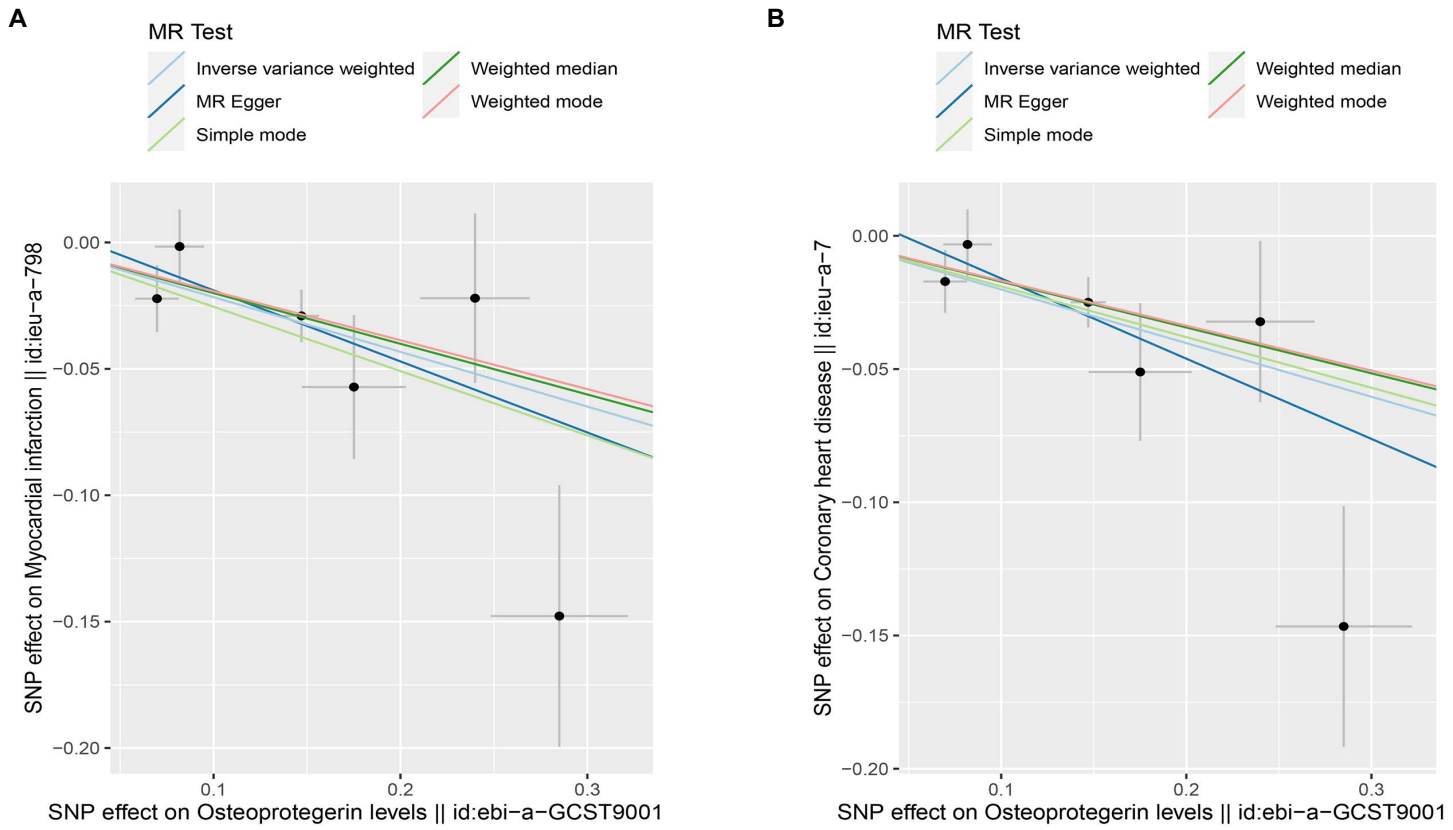
Scatterplot of MR estimates of genetic risk of OPG on AMI and CHD after removing rs1385492. Scatter-plot of genetic effects on OPG versus their effects on AMI **(A)** and CHD**(B)** with corresponding standard errors denoted by horizontal and vertical lines. The slope of each line corresponds to the estimated MR effect from different methods.

**Figure 10:**
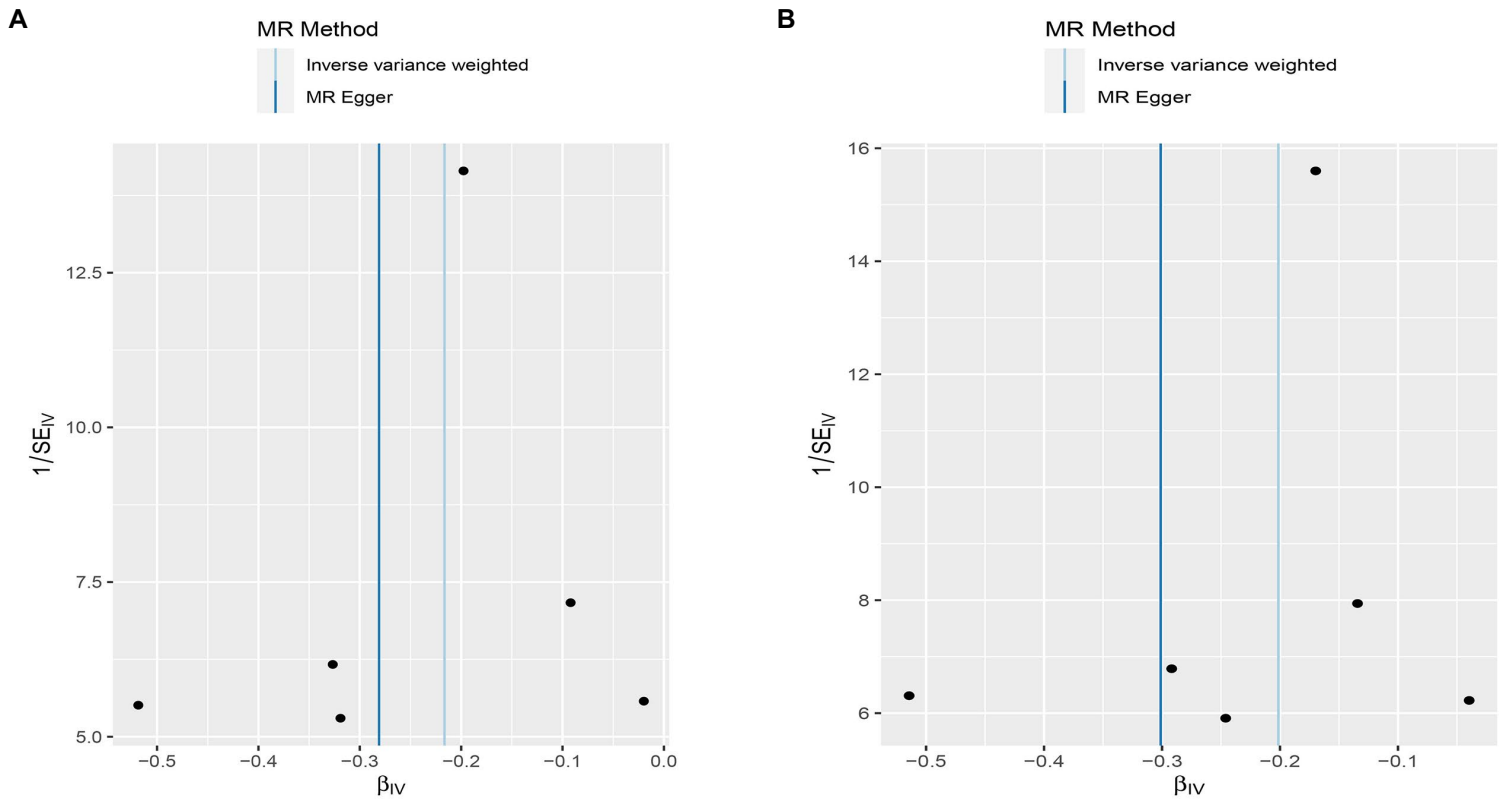
Funnel plot MR estimates of genetic risk of OPG on AMI and CHD after removing rs1385492. The funnel plot of genetic effect of OPG relative to its effect on AMI **(A)** and CHD **(B)**, and the distribution of corresponding points reflected the heterogeneity.

**Figure 11:**
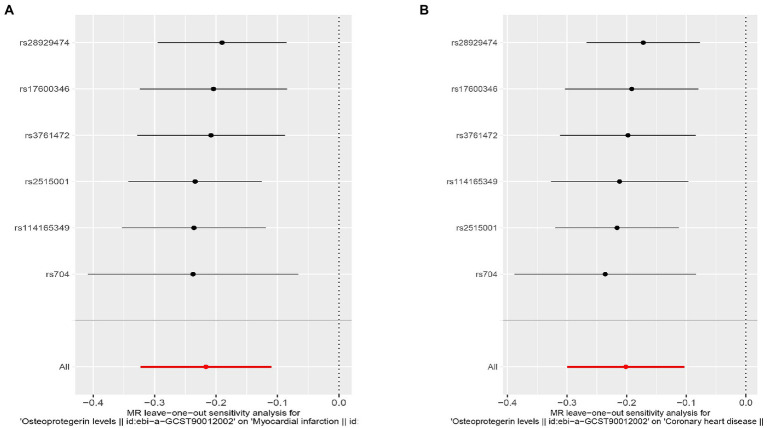
Sensitivity analyses using the leave-one-out approach for the association of plasma OPG level with outcomes after removing rs1385492. **(A)** AMI; **(B)** CHD.

Interestingly, leave-one-out analysis displayed that in AMI and CHD, the null estimates of the weighted median method are driven by rs1385492. Observed significant estimates in the weighted median method support the idea that IVW is biased toward causal inference by outliers (Table 3). As a result, rs1385492 might bias MR estimations of SNPs with seven genome-wide significance. That is to say, after removing the influence of rs1385492, we found that there was a correlation between OPG and AMI/CHD (AMI: weighted median OR = 0.818;95% CI = 0.724–0.950; *p* = 0.001; 6SNPs;CHD:weighted median OR = 0.842; 95% CI = 0.755–0.938; *p* = 1.893×10^−3^; 6SNPs; Table 3; Figure 8). Neither heterogeneity nor pleiotropy was detected in the sensitivity analysis (Supplementary Table S1; Figures 9, 10). No high-impact points were found by Leave-one-out analysis (Figure 11).

**Table 3:** MR associations of genetic determined OPG (*P* < 5 × 10^−8^) with AMI or CHD.

Methods	AMI (6SNPs)			CHD (6SNPs)		
	OR	95%CI	P	OR	95%CI	P
MR Egger	0.755	0.556–1.024	0.145	0.740	0.566–0.966	9.168 × 10^−2^
Weighted median	0.818	0.724–0.950	0.001	0.842	0.755–0.938	1.893 × 10^−3^
Inverse variance weighted	0.805	0.724–0.896	6.820×10^−5^	0.818	0.740–0.902	6.184 × 10^−5^
Simple mode	0.775	0.641–0.937	0.046	0.828	0.701–0.975	7.344 × 10^−2^
Weighted mode	0.824	0.719–0.944	0.038	0.845	0.749–0.952	4.007 × 10^−2^

AMI: acute myocardial infarction; CHD: Coronary Heart Disease.

## Discussion

Consistent with most previous literature, our research found that the genetic tendency of OPG, as a polypeptide associated with the tumor necrosis factor receptor (TNF), is related to the reducing of the risk of AMI and CHD. As a member of the superfamily, OPG is a receptor for nuclear factor κB ligand receptor (RANKL), which is also an avid receptor for TNF-linked apoptosis-inducing ligand (TRAIL). OPG is commonly expressed in bone cells, vascular smooth muscle cells and endothelial cells. It is widely believed that it can be used as a sensitive biomarker of vascular calcification (7). Considering the rising frequency of CHD and AMI each year, the incidence of these conditions is growing, the mortality and disability rate are also high, and more and more scholars are interested in the causes of CHD and AMI. As early as 2004, Toshiki Nagasaki and other scholars believed that OPG was closely related to MI and vascular injury of CHD (20). This study aimed to determine whether OPG correlate with AMI or CHD by using GWAS on a large scale. It was revealed that the OPG had a causal link to CHD or AMI. More recently, Mieczysław Dutka (21) also observed the connection between OPG and AMI or CHD, and showed that OPG was related to the onset of AMI or CHD. The result indicated that OPG, as a single pathogenic factor in patients with AMI and CHD, has a substantial impact on prognosis and considerably affects the development of AMI and CHD. OPG may be the leading participant, not the bystander. And so far, no variant has been found within or near the OPG gene associated with circulating OPG levels.

The presence of calcification in atherosclerotic plaques has been confirmed in a growing number of studies as a factor in the pathogenesis of atherosclerosis, as well as associated with atherosclerosis morbidity. Calcification in this area is affected by the exact regulatory mechanism as that in bone tissue, so OPG and OPG/RANKL axis were initially studied in relation to cardiovascular disorders. The first study to knock out the OPG gene in mice found that along with severe osteoporosis, the aortic wall calcified more rapidly than before. However, opposite results were shown in later clinical research (22). It was found that the high cardiovascular risk in patients with CHD was closely related to high OPG levels (21, 23). RANKL and TRAIL contribute to this association through their mutual interactions with OPG (24–26). Although several clinical experiments have proved that OPG is closely related to CHD or AMI (9, 21, 27), and this relationship is positive, there is still controversy about this relationship in academic circles. In addition, other studies have found an association between OPG and other cardiovascular diseases, including congestive heart failure, aortic stenosis, and aortic valve calcification (8, 28). At the same time, it is not only related to the occurrence of cardiovascular diseases, but also firmly to the prognosis of related cardiovascular diseases (29–33). However, these observational studies are limited to the sample size and experimental design. The causal relationship between OPG and CHD or AMI cannot be obtained.

RCT research is the highest level of epidemiological evidence, and it is also a research design that can best explain causality. However, due to the difficulty of its development, few researchers have done RCT research, plus the considerable human resources and material resources it needs, it is tough to conduct an RCT study. Empirical applications of mendelian randomization in traditional epidemiology skillfully make up for the deficiency of research on epidemiology traditionally in determining the cause of disease; at the same time, it can also make up for the weaknesses of previous observational study, including unavoidable confounding factors and inability to explore causality. At present, it has become one of the best epidemiological means to make up for RCT research (34). Because offspring inherit their genotypes from their parents randomly, SNPs are an excellent tool for analyzing the causal relationship between two factors (35). Our research enriched the literature on the relationship between OPG and MI or CHD. Firstly, we used the MR method to provide evidence for the causal relationship between OPG and AMI or CHD. Especially the causal relationship among them, IVW, weighted median and MR-Egger were provided to clarify the causal relationship among them. Although the weighted median method confirmed no causal relationship between OPG and AMI or CHD at the beginning of the study, with the deepening of the research, we found that rs1385492 was an important tool variable that affected the overall research results. After removing the influence of rs1385492, a reliable causal relationship among the three factors was obtained. Secondly, rs1385492 is related to TNF by consulting the GWAS catalog. It is well known that TNF plays a crucial important role in causing and developing the disease in cardiovascular medicine (36–38).

Our research also has limitations, mainly in the following aspects. First of all, we used the dataset of GWAS, not a single raw data, which caused many inconveniences in the analysis, the most significant of which is that subgroup analysis is impossible. AMI and CHD have many subtypes, for example, AMI including 5 types (Thrombosis in coronary artery caused by rupture, crack or dissection of coronary plaque leads to spontaneous myocardial infarction is designated as a type 1 MI; type 2 MI is the pathophysiological process that leads to ischemic myocardium damage in the setting of an imbalance between oxygen supply and demand; because death has occurred, patients suspected of sudden cardiac death due to myocardial ischemia, or suspected of cardiac death due to new ECG ischemic changes or new LBBB have no time to collect blood samples for myocardial marker determination, which is defined as type 3 MI; type 4 is defined as myocardial infarction related to PCI; type 5 is defined as AMI related to coronary artery bypass grafting.) (39). Secondly, we solely analyzed the link between OPG and AMI or CHD in terms of genetic determinants and did not take into account a number of environmental confounding variables. Thirdly, In the absence of a comprehensive knowledge of the biological function of the chosen SNP, a pleiotropy hypothesis cannot be ruled out completely. In spite of this, it is pleasing that the effect estimation was robust across different MR models, and the IVW sensitivity analysis array failed to detect any pleiotropy when applied to our research. Finally, Population stratification, dynastic mating, and assortative mating should be considered, since they may lead to confusion as they introduce false causality.

## Conclusion

Generally speaking, our results supported that OPG is a casual risk factor for CHD or AMI. This causal relationship provided us with new ideas in the future research field of the etiology of AMI and CHD.

## Data availability statement

The datasets presented in this study can be found in online repositories. The names of the repository/repositories and accession number(s) can be found at: https://www.ebi.ac.uk/gwas/.

## Ethics statement

Ethical approval was not required because only summary-level data were used in our study.

## Author contributions

PC and YY: writing—original draft preparation. LZ, XZ, and XC: validation. PC and SW: writing—review and editing. All authors contributed to the article and approved the submitted version.

## Conflict of interest

The authors declare that the research was conducted in the absence of any commercial or financial relationships that could be construed as a potential conflict of interest.

## Publisher’s note

All claims expressed in this article are solely those of the authors and do not necessarily represent those of their affiliated organizations, or those of the publisher, the editors and the reviewers. Any product that may be evaluated in this article, or claim that may be made by its manufacturer, is not guaranteed or endorsed by the publisher.

## Footnotes

^1^ https://www.ebi.ac.uk/gwas/

^2^ https://sb452.shinyapps.io/overlap/
