# Supplementary material for: Causal effects for genetic variants of osteoprotegerin on the risk of acute myocardial infarction and coronary heart disease: A two-sample Mendelian randomization study
Source: Front Cardiovasc Med. 2023 Mar 7;10:1041231. doi: 10.3389/fcvm.2023.1041231 (PMC10028206; doi:10.3389/fcvm.2023.1041231)
Supplement: Supplementary file 1 [file Data_Sheet_1.PDF]

Large-scale association analysis identifies new risk loci for coronary artery disease

Panos Deloukas[1,126], Stavroula Kanoni[1,126], Christina Willenborg[2,126], Martin Farrall[3,4,126], Themistocles L Assimes[5,126], John R Thompson[6,126], Erik Ingelsson[7,126], Danish Saleheen[8–10,126], Jeanette Erdmann[2,126], Benjamin A Goldstein[5], Kathleen Stirrups[1], Inke R König[11], Jean-Baptiste Cazier[4], Åsa Johansson[12], Alistair S Hall[13], Jong-Young Lee[14], Cristen J Willer[15,16], John C Chambers[17], Tõnu Esko[18,19], Lasse Folkersen[20,21], Anuj Goel[3,4], Elin Grundberg[22], Aki S Havulinna[23], Weang K Ho[10], Jemma C Hopewell[24,25], Niclas Eriksson[12], Marcus E Kleber[26,27], Kati Kristiansson[23], Per Lundmark[28], Leo-Pekka Lyytikäinen[29,30], Suzanne Rafelt[31], Dmitry Shungin[32–34], Rona J Strawbridge[20,21], Gudmar Thorleifsson[35], Emmi Tikkanen[36,37], Natalie Van Zuydam[38], Benjamin F Voight[39], Lindsay L Waite[40], Weihua Zhang[17], Andreas Ziegler[11], Devin Absher[40], David Altshuler[41–44], Anthony J Balmforth[45], Inês Barroso[1,46], Peter S Braund[31,47], Christof Burgdorf[48], , Simone Claudi-Boehm[49], David Cox[50], Maria Dimitriou[51], Ron Do[41,43], CARDIOGENICS Consortium[52], DIAGRAM Consortium[52], Alex S F Doney[38], NourEddine El Mokhtari[53], Per Eriksson[20,21], Krista Fischer[18], Pierre Fontanillas[41], Anders Franco-Cereceda[54], Bruna Gigante[55], Leif Groop[56], Stefan Gustafsson[7], Jörg Hager[57], Göran Hallmans[58], Bok-Ghee Han[14], Sarah E Hunt[1], Hyun M Kang[59], Thomas Illig[60], Thorsten Kessler[48], Joshua W Knowles[5], Genovefa Kolovou[61], Johanna Kuusisto[62], Claudia Langenberg[63], Cordelia Langford[1], Karin Leander[55], Marja-Liisa Lokki[64], Anders Lundmark[28], Mark I McCarthy[3,65,66], Christa Meisinger[67], Olle Melander[56], Evelin Mihailov[19], Seraya Maouche[68], Andrew D Morris[38], Martina Müller-Nurasyid[69–72], MuTHER Consortium[52], Kjell Nikus[73], John F Peden[3], N William Rayner[3], Asif Rasheed[9], Silke Rosinger[74], Diana Rubin[53], Moritz P Rumpf[48], Arne Schäfer[75], Mohan Sivananthan[76,77], Ci Song[7], Alexandre F R Stewart[78,79], Sian-Tsung Tan[80], Gudmundur Thorgeirsson[81,82], C Ellen van der Schoot[83], Peter J Wagner[36,37], Wellcome Trust Case Control Consortium[52], George A Wells[78,79], Philipp S Wild[84,85], Tsun-Po Yang[1], Philippe Amouyel[86], Dominique Arveiler[87], Hanneke Basart[88], Michael Boehnke[59], Eric Boerwinkle[89], Paolo Brambilla[90], Francois Cambien[68], Adrienne L Cupples[91,92], Ulf de Faire[55], Abbas Dehghan[93], Patrick Diemert[94], Stephen E Epstein[95], Alun Evans[96], Marco M Ferrario[97], Jean Ferrières[98], Dominique Gauguier[3,99], Alan S Go[100], Alison H Goodall[31,47], Villi Gudnason[81,101], Stanley L Hazen[102], Hilma Holm[35], Carlos Iribarren[100], Yangsoo Jang[103], Mika Kähönen[104], Frank Kee[105], Hyo-Soo Kim[106], Norman Klopp[60], Wolfgang Koenig[107], Wolfgang Kratzer[108], Kari Kuulasmaa[23], Markku Laakso[62], Reijo Laaksonen[108], Ji-Young Lee[14], Lars Lind[28], Willem H Ouwehand[1,109,110], Sarah Parish[24,25], Jeong E Park[111], Nancy L Pedersen[7], Annette Peters[67,112], Thomas Quertermous[5], Daniel J Rader[113], Veikko Salomaa[23], Eric Schadt[114], Svati H Shah[115,116], Juha Sinisalo[117], Klaus Stark[118], Kari Stefansson[35,81], David-Alexandre Trégouët[68], Jarmo Virtamo[23], Lars Wallentin[12], Nicholas Wareham[63], Martina E Zimmermann[118], Markku S Nieminen[117], Christian Hengstenberg[118], Manjinder S Sandhu[1,63], Tomi Pastinen[119], Ann-Christine Syvänen[28], G Kees Hovingh[88], George Dedoussis[51], Paul W Franks[32–34,120], Terho Lehtimäki[29,30], Andres Metspalu[18,19], Pierre A Zalloua[121], Agneta Siegbahn[12], Stefan Schreiber[94], Samuli Ripatti[1,37], Stefan S Blankenberg[74], Markus Perola[23], Robert Clarke[24,25], Bernhard O Boehm[74], Christopher O'Donnell[93], Muredach P Reilly[122,126], Winfried März[26,123], Rory Collins[24,25,127], Sekar Kathiresan[41,124,125,126], Anders Hamsten[20,21,126], Jaspal S Kooner[80,126], Unnur Thorsteinsdottir[35,81,126], John Danesh[9, 126], Colin N A Palmer[38,126], Robert Roberts[78,79,126], Hugh Watkins[3,4,126], Heribert Schunkert[2,126] & Nilesh J Samani[31,47,126]

[1]Wellcome Trust Sanger Institute, Hinxton, Cambridge, UK. [2]Institut für Integrative und Experimentelle Genomik, Universität zu Lübeck, Lübeck, Germany. [3]Wellcome Trust Centre for Human Genetics, University of Oxford, Oxford, UK. [4]Cardiovascular Medicine, Radcliffe Department of Medicine, University of Oxford, Oxford, UK. [5]Department of Medicine, Stanford University School of Medicine, Stanford, California, USA. [6]Department of Health Sciences, University of Leicester, Leicester, UK. [7]Department of Medical Epidemiology and Biostatistics, Karolinska Institutet, Stockholm, Sweden. [8]Center for Non-Communicable Diseases, Karachi, Pakistan. [9]Department of Public Health and Primary

Nature Genetics: doi:10.1038/ng.2480

Care, University of Cambridge, Cambridge, UK. [10]Department of Medicine, University of Pennsylvania, Philadelphia, Pennsylvania, USA. [11]Institut für Medizinische Biometrie und Statistik, Universität zu Lübeck, Lübeck, Germany. [12]Uppsala Clinical Research Center, Uppsala University, Uppsala, Sweden. [13]Division of Cardiovascular and Neuronal Remodelling, Multidisciplinary Cardiovascular Research Centre, Leeds Institute of Genetics, Health and Therapeutics, University of Leeds, Leeds, UK. [14]Center for Genome Science, Korea National Institute of Health, Korea Center for Disease Control and Prevention, Yeonje-ri, Chungwon-gun, Chungcheongbuk-do, Korea. [15]Division of Cardiovascular Medicine, Department of Internal Medicine, University of Michigan, Ann Arbor, Michigan, USA. [16]Department of Human Genetics, University of Michigan, Ann Arbor, Michigan, USA. [17]Department of Epidemiology and Biostatistics, Imperial College London, London, UK. [18]Estonian Genome Center, University of Tartu, Tartu, Estonia. [19]Institute of Molecular and Cell Biology, University of Tartu, Tartu, Estonia. [20]Atherosclerosis Research Unit, Department of Medicine, Karolinska Institutet, Stockholm, Sweden. [21]Center for Molecular Medicine, Karolinska University Hospital, Stockholm, Sweden. [22]Department of Twin Research and Genetic Epidemiology, King's College London, London, UK. [23]Department of Chronic Disease Prevention, National Institute for Health and Welfare, Helsinki, Finland. [24]Clinical Trial Service Unit, University of Oxford, Oxford, UK. [25]Epidemiological Studies Unit, University of Oxford, Oxford, UK. [26]Mannheim Institute of Public Health, Social and Preventive Medicine, Medical Faculty of Mannheim, University of Heidelberg, Mannheim, Germany. [27]Ludwigshafen Risk and Cardiovascular Health (LURIC) Study, Freiburg, Germany. [28]Department of Medical Sciences, Uppsala University, Uppsala, Sweden. [29]Department of Clinical Chemistry, Fimlab Laboratories, Tampere University Hospital, Tampere, Finland. [30]Department of Clinical Chemistry, University of Tampere School of Medicine, Tampere, Finland. [31]Department of Cardiovascular Sciences, University of Leicester, Glenfield Hospital, Leicester, UK. [32]Genetic & Molecular Epidemiology Unit, Department of Clinical Sciences, Lund University Diabetes Center, Skåne University Hospital, Malmö, Sweden. [33]Department of Public Health & Clinical Medicine, Genetic Epidemiology & Clinical Research Group, Section for Medicine, Umeå University, Umeå, Sweden. [34]Department of Odontology, Umeå University, Umeå, Sweden. [35]deCODE Genetics, Reykjavik, Iceland. [36]Institute for Molecular Medicine FIMM, University of Helsinki, Helsinki, Finland. [37]Public Health Genomics Unit, National Institute for Health and Welfare, Helsinki, Finland. [38]Medical Research Institute, University of Dundee, Ninewells Hospital and Medical School, Dundee, UK. [39]Department of Pharmacology, University of Pennsylvania, Philadelphia, Pennsylvania, USA. [40]HudsonAlpha Institute for Biotechnology, Huntsville, Alabama, USA. [41]Broad Institute of Harvard and MIT, Cambridge, Massachusetts, USA. [42]Department of Molecular Biology, Massachusetts General Hospital, Boston, Massachusetts, USA. [43]Center for Human Genetic Research, Massachusetts General Hospital, Boston, Massachusetts, USA. [44]Department of Genetics, Harvard Medical School, Boston, Massachusetts, USA. [45]Division of Cardiovascular and Diabetes

Nature Genetics: doi:10.1038/ng.2480

Research, Multidisciplinary Cardiovascular Research Centre, Leeds Institute of Genetics, Health and Therapeutics, University of Leeds, Leeds, UK. [46]*University of Cambridge Metabolic Research Laboratories, Institute of Metabolic Science, Addenbrooke's Hospital, Cambridge, UK.* [47]*National Institute for Health Research (NIHR) Leicester Cardiovascular Biomedical Research Unit, Glenfield Hospital, Leicester, UK.* [48] *Deutsches Herzzentrum München, Technische Universität München, Munich, Germany.* [49]*Practice of Gynecology, Ulm University Medical Centre, Ulm, Germany.* [50]*Biotherapeutics and Bioinnovation Center, Pfizer,* South San Francisco, California, USA. [51]*Department of Dietetics–Nutrition, Harokopio University, Athens, Greece.* [52]*A list of members and affiliations appears in the* **Supplementary Note**. [53]*Klinik für Innere Medizin, Kreiskrankenhaus Rendsburg, Rendsburg, Germany.* [54]*Cardiothoracic Surgery Unit, Department of Molecular Medicine and Surgery, Karolinska Institutet, Stockholm, Sweden.* [55]*Division of Cardiovascular Epidemiology, Institute of Environmental Medicine, Karolinska Institutet, Stockholm, Sweden.* [56]*Department of Clinical Sciences, Diabetes and Endocrinology, Lund University, University Hospital Malmö, Malmö, Sweden.* [57]*CEA–Genomics Institute, National Genotyping Centre, Paris, France.* Commissariat à l'énergie atomique et aux énergies alternatives*]* [58]*Department of Public Health & Clinical Medicine, Section for Nutritional Research, Umeå University, Umeå, Sweden.* [59]*Department of Biostatistics, Center for Statistical Genetics, University of Michigan, Ann Arbor, Michigan, USA.* [60]*Hannover Unified Biobank, Hannover Medical School, Hannover, Germany.* [61]*First Cardiology Department, Onassis Cardiac Surgery Center 356, Athens, Greece.* [62]*Department of Medicine, University of Eastern Finland and Kuopio University Hospital, Kuopio, Finland.* [63]*Medical Research Council (MRC) Epidemiology Unit, Institute of Metabolic Science, Addenbrooke's Hospital, Cambridge, UK.* [64]*Transplantation Laboratory, Haartman Institute, University of Helsinki, Helsinki, Finland.* [65]*Oxford Centre for Diabetes, Endocrinology and Metabolism, University of Oxford, Oxford, UK.* [66]*Oxford NIHR Biomedical Research Centre, Churchill Hospital, Oxford, UK.* [67]*Institute of Epidemiology II, Helmholtz Zentrum München–German Research Center for Environmental Health, Neuherberg, Germany.* [68]*Institut National de la Santé et la Recherche Médicale (INSERM) Unité Mixte de Recherche (UMR) S937, Institute for Cardiometabolism and Nutrition (ICAN), Pierre and Marie Curie (Paris 6) University, Paris, France.* [69]*Department of Medicine I, University Hospital Grosshadern, Ludwig-Maximilians-Universität, Munich, Germany.* [70]*Chair of Epidemiology, Institute of Medical Informatics, Biometry and Epidemiology, Ludwig-Maximilians-Universität, Munich, Germany.* [71]*Chair of Genetic Epidemiology, Institute of Medical Informatics, Biometry and Epidemiology, Ludwig-Maximilians-Universität, Munich, Germany.* [72]*Institute of Genetic Epidemiology, Helmholtz Zentrum München–German Research Center for Environmental Health, Neuherberg, Germany.* [73]*Heart Centre, Department of Cardiology, Tampere University Hospital, Tampere, Finland.* [74]*Division of Endocrinology and Diabetes, Department of Internal Medicine, Ulm University Medical Centre, Ulm, Germany.* [75]*Institut für Klinische Molekularbiologie, Christian-Albrechts Universität, Kiel, Germany.* [76]*Division of*

Nature Genetics: doi:10.1038/ng.2480

Epidemiology, Multidisciplinary Cardiovascular Research Centre (MCRC) University of Leeds, Leeds, UK. [77]*Leeds Institute of Genetics, Health and Therapeutics, University of Leeds, Leeds, UK.* [78]*University of Ottawa Heart Institute, Cardiovascular Research Methods Centre Ontario, Ottawa, Ontario, Canada.* [79]*Ruddy Canadian Cardiovascular Genetics Centre, Ottawa, Ontario, Canada.* [80]*National Heart and Lung Institute (NHLI), Imperial College London, Hammersmith Hospital, London, UK.* [81]*Faculty of Medicine, University of Iceland, Reykjavik, Iceland.* [82]*Department of Medicine, Landspitali University Hospital, Reykjavik, Iceland.* [83]*Department of Experimental Immunohematology, Sanquin, Amsterdam, The Netherlands.* [84]*Center for Thrombosis and Hemostasis, University Medical Center Mainz, Johannes Gutenberg University Mainz, Mainz, Germany.* [85]*Department of Medicine 2, University Medical Center Mainz, Johannes Gutenberg University Mainz, Mainz, Germany.* [86]*Institut Pasteur de Lille, INSERM U744, Université Lille Nord de France, Lille, France.* [87]*Department of Epidemiology and Public Health, EA3430, University of Strasbourg, Strasbourg, France.* [88]*Department of Vascular Medicine, Academic Medical Center, Amsterdam, The Netherlands.* [89]*Human Genetics Center, University of Texas Health Science Center, Houston, Texas, USA.* [90]*Department of Experimental Medicine, University of Milano–Bicocca, Monza, Italy.* [91]*Department of Biostatistics, Boston University School of Public Health, Boston, Massachusetts, USA.* [92]*National Heart, Lung, and Blood Institute's Framingham Heart Study, Framingham, Massachusetts, USA.* [93]*Department of Epidemiology, Erasmus Medical Center, Rotterdam, The Netherlands.* [94]*Clinic for General and Interventional Cardiology, University Heart Center Hamburg, Hamburg, Germany.* [95]*Cardiovascular Research Institute, Washington Hospital Center, Washington, DC, USA.* [96]*Centre for Public Health, The Queen's University of Belfast, Belfast, UK.* [97]*Research Centre for Epidemiology and Preventive Medicine (EPIMED), Department of Clinical and Experimental Medicine, University of Insubria, Varese, Italy.* [98]*Department of Cardiology, Toulouse University School of Medicine, Rangueil Hospital, Toulouse, France.* [99]*INSERM UMR S872, Cordeliers Research Centre, Paris, France.* [100]*Division of Research, Kaiser Permanente Northern California, Oakland, California, USA.* [101]*Icelandic Heart Association, Kopavogur, Iceland.* [102]*Lerner Research Institute, Cleveland Clinic, Cleveland, Ohio, USA.* [103]*Cardiology Division, Department of Internal Medicine, Cardiovascular Genome Center, Yonsei University, Seoul, Korea.* [104]*Department of Clinical Physiology, Tampere University Hospital and University of Tampere, Tampere, Finland.* [105]*UK Clinical Research Collaboration (UKCRC) Centre of Excellence for Public Health (Northern Ireland), Queen's University of Belfast, Belfast, UK.* [106]*Department of Internal Medicine, Cardiovascular Center, Seoul National University Hospital, Seoul, Korea.* [107]*Department of Internal Medicine II–Cardiology, Ulm University Medical Center, Ulm, Germany.* [108]*Science Center, Tampere University Hospital, Tampere, Finland.* [109]*Department of Haematology, University of Cambridge, Cambridge, UK.* [110]*National Health Service (NHS) Blood and Transplant, Cambridge, UK.* [111]*Division of Cardiology, Samsung Medical Center, Seoul, Korea.* [112]*Munich Heart Alliance, Munich, Germany.* [113]*Division of Translational Medicine and*

Nature Genetics: doi:10.1038/ng.2480

Human Genetics, Department of Medicine, Perelman School of Medicine at the University of Pennsylvania, Philadelphia, Pennsylvania, USA. [114]*Institute for Genomics and Multiscale Biology, Department of Genetics and Genomic Sciences, Mount Sinai School of Medicine, New York, New York, USA.* [115]*Center for Human Genetics, Department of Medicine, Duke University Medical Center, Durham, North Carolina, USA.* [116]*Division of Cardiology, Department of Medicine, Duke University Medical Center, Durham, North Carolina, USA.* [117]*Division of Cardiology, Department of Medicine, Helsinki University Central Hospital (HUCH), Helsinki, Finland.* [118]*Klinik und Poliklinik für Innere Medizin II, Regensburg, Germany.* [119]*Department of Human Genetics, McGill University, Montréal, Québec, Canada.* [120]*Department of Nutrition, Harvard School of Public Health, Boston, Massachusetts, USA.* [121]*Lebanese American University, Chouran, Beirut, Lebanon.* [122]*Cardiovascular Institute, Perelman School of Medicine at the University of Pennsylvania, Philadelphia, Pennsylvania, USA.* [123]*Synlab Academy, Mannheim, Germany.* [124]*Cardiology Division, Center for Human Genetic Research, Massachusetts General Hospital and Harvard Medical School, Boston, Massachusetts, USA.* [125]*Cardiovascular Research Center, Massachusetts General Hospital and Harvard Medical School, Boston, Massachusetts, USA.* [126]*These authors contributed equally to this work.*

Collaborators

Stephen P Fortman[100], Dong-Jik Shin[103], Christopher P Nelson[31], Kyung W Park[106], Bok-Soo Lee[111], Mark A Hlatky[5]

Supplementary Figures

Supplementary Figure 1: Flow-chart of the CARDIoGRAMplusC4D study

Supplementary Figure 2: Histogram of the absolute meta-analysis z-scores for the replication SNPs in Stage 2

Supplementary Figure 3: Forest plots of the 15 novel coronary artery disease loci

Supplementary Figure 4: Regional association plots of the 15 novel CAD loci

Supplementary Figure 5: Histogram of ORs for the104 SNPs at 5% FDR (LD threshold of $r^2 < 0.2$)

Supplementary Figure 6: Diagram depicting part of the overlapping network ON1 and the interactions recorded in the Ingenuity data base between constituent genes

Supplementary Figure 7: Density histogram of p2_final for the 54,803 SNPs considered in the FDR analysis

Supplementary Tables

Nature Genetics: doi:10.1038/ng.2480

Supplementary Table 1a/b: Description of the participating studies

Supplementary Table 2a/b: General study characteristics

Supplementary Table 3a/b: Genotyping, quality control and statistical analysis

Supplementary Table 4: Loci not reaching genome-wide significance in Stage 3

Supplementary Table 5: Subgroup analyses

Supplementary Table 6: Expression Analyses

Supplementary Table 7a: Mouse Model Details for Novel Loci

Supplementary Table 7b: Mouse Model Details for Known Loci

Supplementary Table 8: Overlap between CAD and other traits

Supplementary Table 9: SNPs at an FDR≤5% and LD threshold of $r^2 < 0.2$ used in estimating heritability

Supplementary Table 10: Network molecules

Supplementary Note

1. Study characteristics

2. Background information on novel coronary artery disease risk loci

3. Network analysis in genes not associated to CAD

4. Sources of Funding

5. Consortia

Nature Genetics: doi:10.1038/ng.2480

Supplementary Figures

Supplementary Figure 1: Flow-chart of the CARDIoGRAMplusC4D study. The 196,725 SNP markers on the Metabochip array were selected by a number of consortia working on cardiometabolic traits with the aim to perform extensive replication of initial signals from large GWA meta-analyses and undertake fine-mapping in already known loci. Circa 30% of the markers on this array were not polymorphic in Caucasian studies mainly due to the use of an early release of the 1000 Genomes (July 2009) for selecting SNPs for fine-mapping analysis. Blue arrows mark the path of analysing the 79,138 SNPs with data in both Stage 1 and 2 studies (includes all replication SNPs submitted by CARDIoGRAM) whereas red arrows mark the path of analysing the remaining 49,444 polymorphic SNPs with data only in stage 2 studies (mainly those profiled with the Metabochip array).

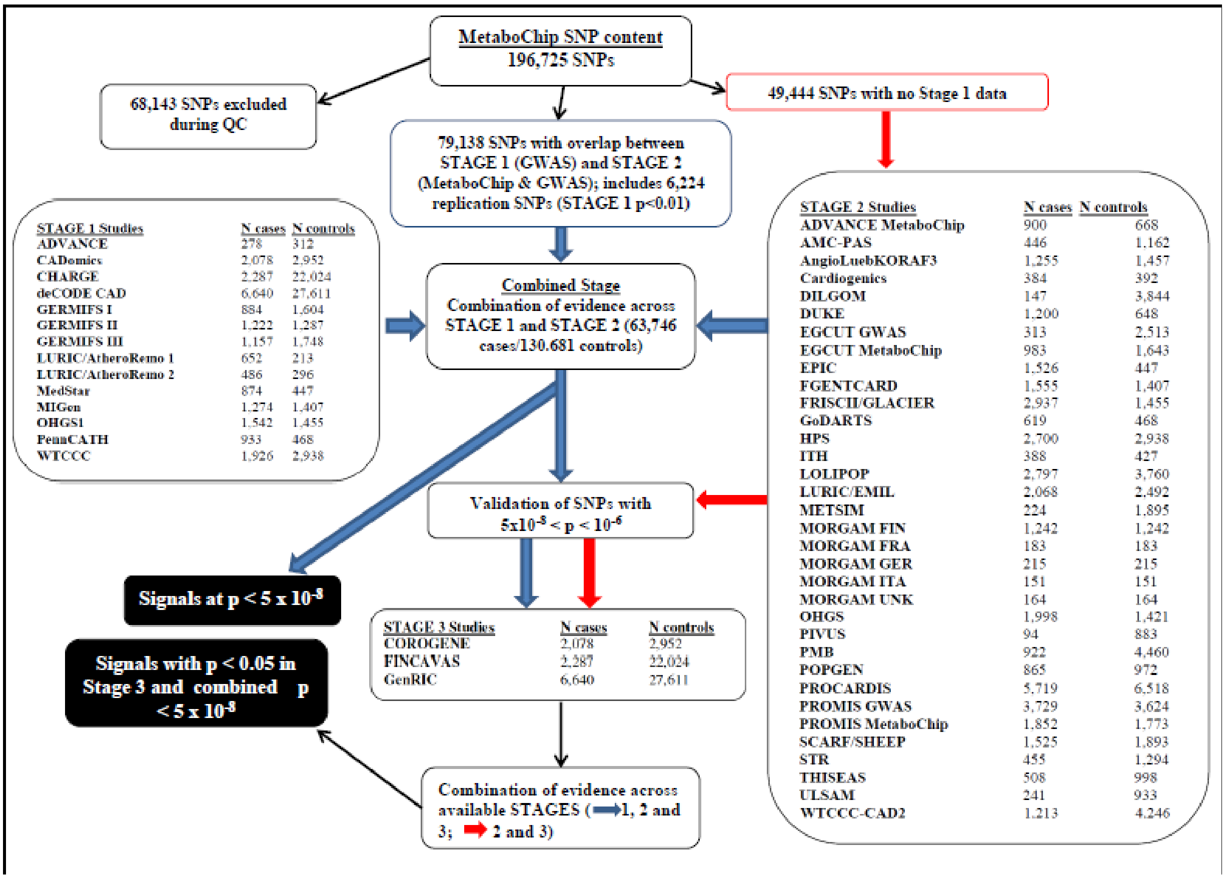

Supplementary Figure 2: Histogram of the absolute meta-analysis z-scores for the replication SNPs in Stage 2; positive/negative z-scores indicate directional consistency/ inconsistency with Stage 1, respectively.

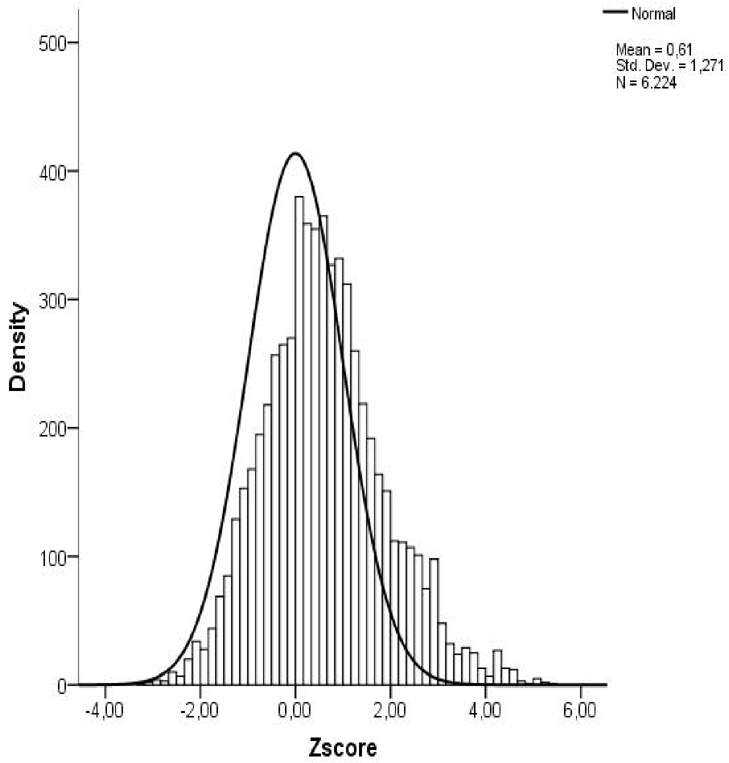

Nature Genetics: doi:10.1038/ng.2480

Supplementary Figure 3: Forest plots of the 15 novel coronary disease loci. Single-study boxes and lines indicate odds ratios and 95% confidence intervals. Box sizes are determined by the weight of the study. Explanation of study abbreviations is provided in Supplementary Table 1.

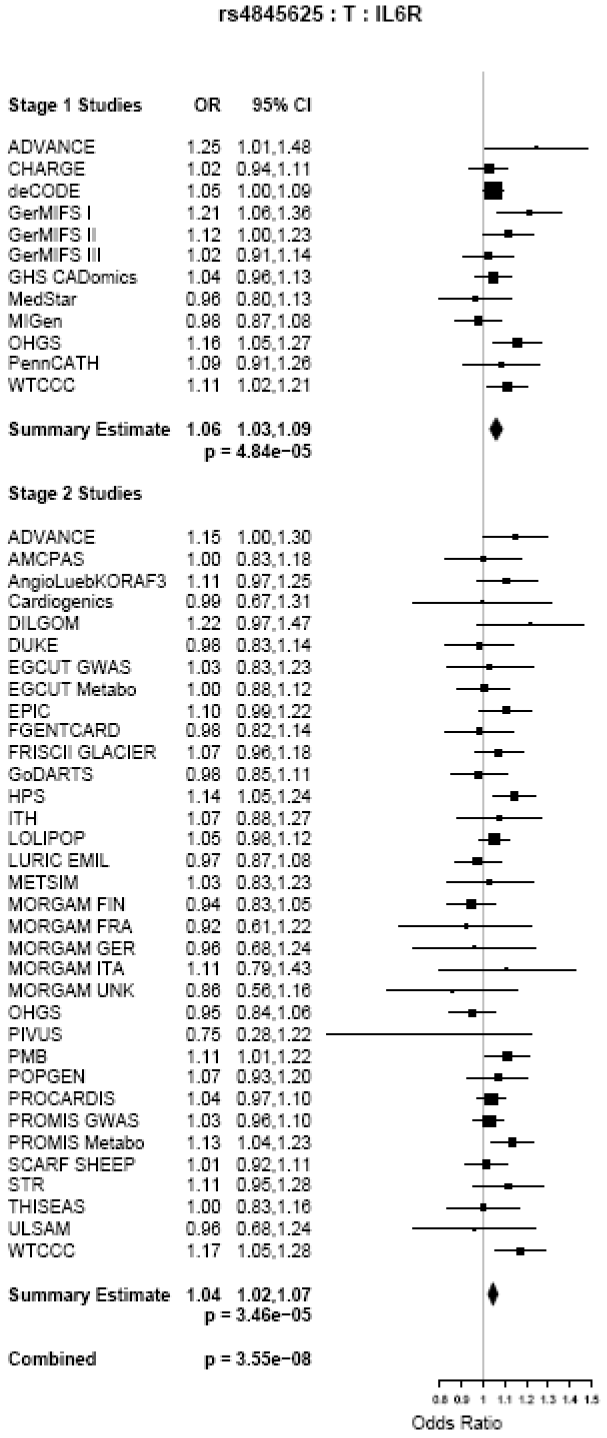

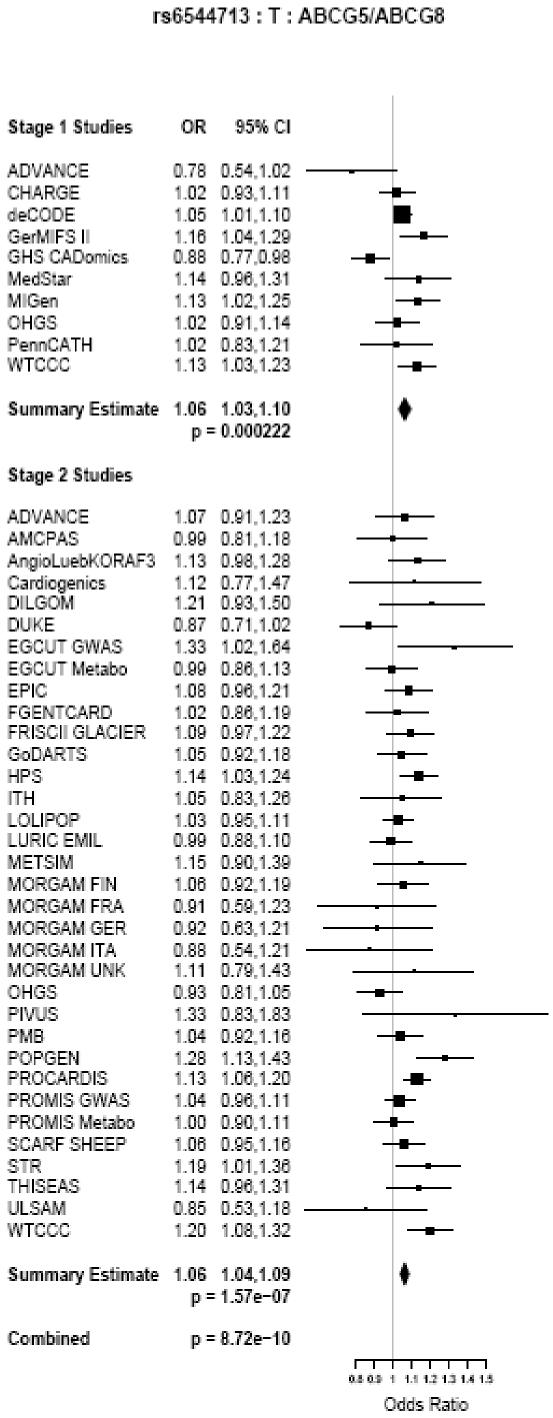

Nature Genetics: doi:10.1038/ng.2480

rs515135 : G : APOB

Stage 1 Studies	OR	95% CI
ADVANCE	1.10	0.78,1.41
CHARGE	1.06	0.94,1.17
deCODE	1.06	1.00,1.13
GerMIFS I	1.15	0.96,1.34
GerMIFS II	1.12	0.98,1.27
GerMIFS III	1.01	0.87,1.16
GHS CADomics	1.05	0.94,1.16
MedStar	0.96	0.75,1.18
MIGen	0.95	0.81,1.09
OHGS	1.34	1.20,1.48
PennCATH	0.90	0.67,1.13
WTCCC	1.07	0.95,1.20
**Summary Estimate**	**1.07**	**1.03,1.10**
		p = 0.000863

Stage 2 Studies	OR	95% CI
ADVANCE	1.06	0.87,1.24
AMCPAS	1.09	0.85,1.32
AngioLuebKORAF3	0.99	0.80,1.17
Cardiogenics	1.36	0.93,1.80
DILGOM	1.20	0.88,1.52
EGCUT GWAS	0.93	0.64,1.21
EGCUT Metabo	1.02	0.86,1.18
EPIC	1.04	0.89,1.20
FGENTCARD	1.01	0.81,1.20
FRISCII GLACIER	0.99	0.83,1.14
GoDARTS	1.12	0.95,1.28
HPS	1.09	0.97,1.22
ITH	1.21	0.95,1.47
LOLIPOP	1.08	0.98,1.18
LURIC EMIL	1.04	0.90,1.18
METSIM	1.08	0.83,1.33
MORGAM FIN	1.11	0.97,1.25
MORGAM FRA	1.09	0.68,1.50
MORGAM GER	1.21	0.83,1.60
MORGAM ITA	1.50	1.08,1.92
MORGAM UNK	1.14	0.73,1.54
OHGS	0.97	0.83,1.11
PIVUS	0.85	0.22,1.49
PMB	1.29	1.14,1.44
POPGEN	1.23	1.06,1.41
PROCARDIS	1.18	1.10,1.27
PROMIS GWAS	1.01	0.92,1.10
PROMIS Metabo	1.10	0.97,1.23
SCARF SHEEP	1.05	0.92,1.19
STR	1.08	0.86,1.30
THISEAS	1.01	0.81,1.23
ULSAM	1.40	0.99,1.80
WTCCC	1.06	0.91,1.21
**Summary Estimate**	**1.08**	**1.05,1.11**
		p = 2.17e−08
**Combined**		p = 4.8e−10

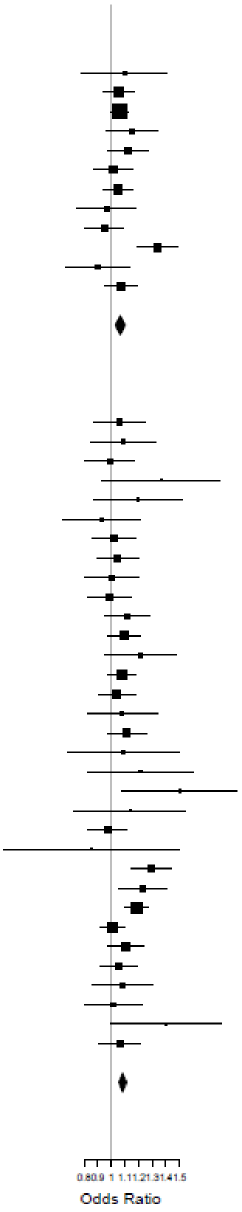

rs15563 : C : UBE2Z

Stage 1 Studies	OR	95% CI
ADVANCE	0.98	0.75,1.21
CHARGE	1.07	0.99,1.15
deCODE	1.04	1.00,1.08
GerMIFS I	1.03	0.90,1.17
GerMIFS II	1.10	0.99,1.21
GerMIFS III	0.92	0.81,1.03
GHS CADomics	1.12	1.04,1.21
LURIC1	1.39	1.14,1.65
LURIC2	1.21	0.99,1.43
MedStar	0.94	0.74,1.15
MIGen	1.13	1.02,1.24
OHGS	1.06	0.95,1.17
PennCATH	1.04	0.85,1.22
WTCCC	1.13	1.04,1.23
**Summary Estimate**	**1.06**	**1.04,1.09**
		p = 5.01e−06

Stage 2 Studies	OR	95% CI
ADVANCE	0.91	0.76,1.06
AMCPAS	1.17	0.99,1.35
AngioLuebKORAF3	0.98	0.84,1.11
Cardiogenics	1.10	0.77,1.43
DILGOM	0.93	0.69,1.17
DUKE	0.95	0.80,1.10
EGCUT GWAS	1.01	0.82,1.21
EGCUT Metabo	1.05	0.93,1.17
EPIC	1.02	0.91,1.14
FGENTCARD	1.26	1.10,1.42
FRISCII GLACIER	1.11	0.99,1.22
GoDARTS	1.01	0.88,1.14
HPS	0.95	0.86,1.05
ITH	0.81	0.61,1.00
LOLIPOP	1.06	0.99,1.14
LURIC EMIL	1.08	0.98,1.19
METSIM	0.90	0.70,1.10
MORGAM FIN	1.01	0.90,1.12
MORGAM FRA	0.93	0.65,1.21
MORGAM GER	0.79	0.52,1.06
MORGAM ITA	1.07	0.75,1.39
MORGAM UNK	1.21	0.91,1.51
OHGS	0.92	0.81,1.02
PIVUS	1.34	0.86,1.82
PMB	0.98	0.88,1.08
POPGEN	1.13	1.00,1.27
PROCARDIS	0.98	0.91,1.04
PROMIS GWAS	0.99	0.92,1.06
PROMIS Metabo	1.01	0.91,1.10
STR	0.97	0.81,1.13
THISEAS	1.01	0.84,1.17
ULSAM	1.14	0.86,1.41
WTCCC	1.12	1.00,1.23
**Summary Estimate**	**1.01**	**0.99,1.03**
		p = 0.122
**Combined**		p = 9.36e−06

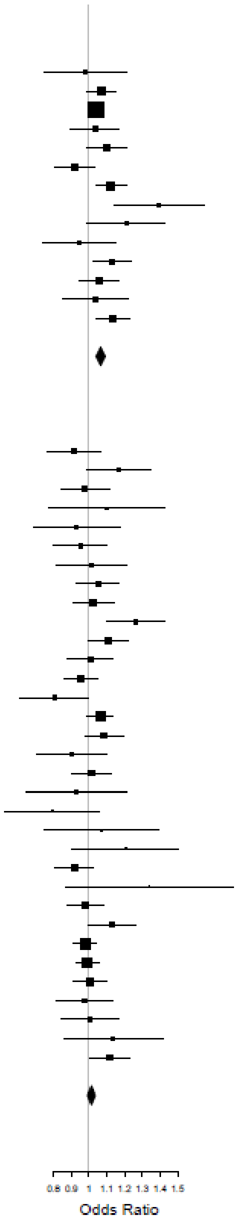

Nature Genetics: doi:10.1038/ng.2480

rs1561198 : A : GGCX/VAMP8

Stage 1 Studies	OR	95% CI
ADVANCE	1.24	0.99,1.49
CHARGE	1.10	1.02,1.18
deCODE	1.04	1.00,1.09
GerMIFS I	1.16	1.01,1.31
GerMIFS II	1.04	0.93,1.16
GerMIFS III	0.93	0.82,1.04
GHS CADomics	1.04	0.95,1.12
LURIC1	1.24	0.99,1.49
MedStar	1.16	0.95,1.37
MIGen	1.05	0.95,1.16
OHGS	1.03	0.92,1.14
PennCATH	1.09	0.91,1.27
WTCCC	1.09	1.00,1.18
**Summary Estimate**	1.06	1.03,1.08
		p = 7.47e−05
**Stage 2 Studies**		
ADVANCE	0.98	0.83,1.13
AMCPAS	1.28	1.11,1.46
AngioLuebKORAF3	1.05	0.91,1.19
Cardiogenics	1.22	0.90,1.55
DILGOM	1.04	0.81,1.28
DUKE	0.97	0.81,1.12
EGCUT GWAS	1.01	0.80,1.21
EGCUT Metabo	1.06	0.94,1.18
EPIC	1.12	1.00,1.24
FGENTCARD	1.06	0.90,1.22
FRISCII GLACIER	1.07	0.96,1.19
GoDARTS	0.96	0.84,1.09
HPS	1.00	0.91,1.10
ITH	0.83	0.63,1.03
LOLIPOP	0.97	0.90,1.05
LURIC EMIL	1.08	0.97,1.18
METSIM	1.02	0.82,1.22
MORGAM FIN	1.10	0.99,1.21
MORGAM FRA	1.09	0.80,1.37
MORGAM GER	0.83	0.56,1.10
MORGAM ITA	0.84	0.52,1.16
MORGAM UNK	0.97	0.67,1.28
OHGS	0.92	0.82,1.03
PIVUS	1.01	0.56,1.45
PMB	1.08	0.98,1.19
POPGEN	1.01	0.87,1.15
PROCARDIS	1.09	1.03,1.16
PROMIS GWAS	1.09	1.02,1.16
PROMIS Metabo	1.11	1.02,1.21
SCARF SHEEP	1.11	1.01,1.21
STR	0.97	0.81,1.13
THISEAS	1.09	0.92,1.25
ULSAM	1.32	1.03,1.61
WTCCC	1.13	1.01,1.24
**Summary Estimate**	1.05	1.03,1.07
		p = 2.57e−06
**Combined**		p = 4.48e−09

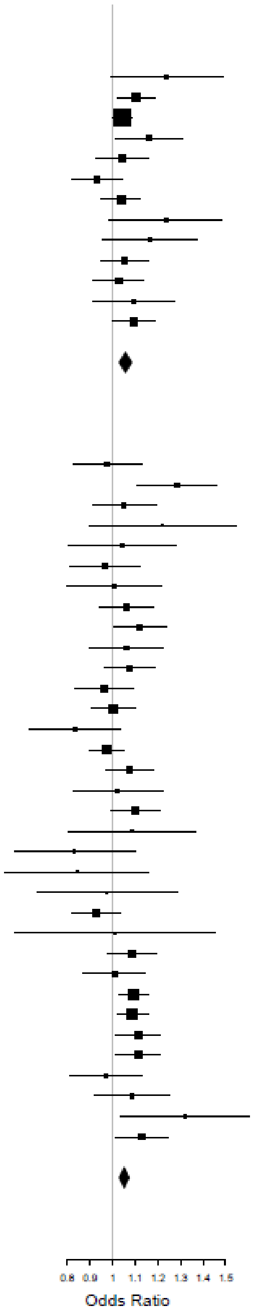

rs7692387 : G : GUCY1A3

Stage 1 Studies	OR	95% CI
ADVANCE	1.03	0.73,1.34
CHARGE	1.08	0.97,1.19
deCODE	1.06	1.01,1.12
GerMIFS I	1.31	1.13,1.49
GerMIFS II	1.11	0.97,1.26
GerMIFS III	1.10	0.95,1.24
GHS CADomics	1.03	0.92,1.14
MedStar	0.98	0.77,1.19
MIGen	1.16	1.03,1.30
OHGS	1.16	1.01,1.30
PennCATH	1.01	0.78,1.24
WTCCC	1.09	0.97,1.21
**Summary Estimate**	1.08	1.05,1.12
		p = 1.04e−05
**Stage 2 Studies**		
ADVANCE	1.11	0.92,1.29
AMCPAS	1.24	1.00,1.48
AngioLuebKORAF3	1.19	1.01,1.37
Cardiogenics	1.04	0.62,1.46
DILGOM	1.08	0.79,1.36
DUKE	0.97	0.77,1.17
EGCUT GWAS	0.94	0.69,1.19
EGCUT Metabo	1.03	0.88,1.18
EPIC	1.02	0.87,1.17
FGENTCARD	1.11	0.90,1.32
FRISCII GLACIER	1.09	0.95,1.24
GoDARTS	0.95	0.79,1.11
HPS	1.10	0.98,1.22
ITH	1.02	0.78,1.27
LOLIPOP	1.15	1.05,1.24
LURIC EMIL	1.12	0.98,1.26
METSIM	1.06	0.83,1.30
MORGAM FIN	0.97	0.84,1.10
MORGAM FRA	0.93	0.59,1.26
MORGAM GER	0.97	0.64,1.31
MORGAM ITA	0.87	0.44,1.29
MORGAM UNK	1.03	0.67,1.40
OHGS	1.00	0.86,1.14
PIVUS	0.92	0.38,1.45
PMB	1.12	0.99,1.26
POPGEN	1.13	0.96,1.30
PROCARDIS	0.97	0.89,1.06
PROMIS GWAS	1.08	1.00,1.17
PROMIS Metabo	1.09	0.97,1.22
SCARF SHEEP	1.01	0.89,1.14
STR	1.12	0.91,1.32
THISEAS	1.03	0.82,1.23
ULSAM	0.91	0.54,1.28
WTCCC	1.14	0.99,1.29
**Summary Estimate**	1.06	1.03,1.09
		p = 1.89e−05
**Combined**		p = 4.57e−09

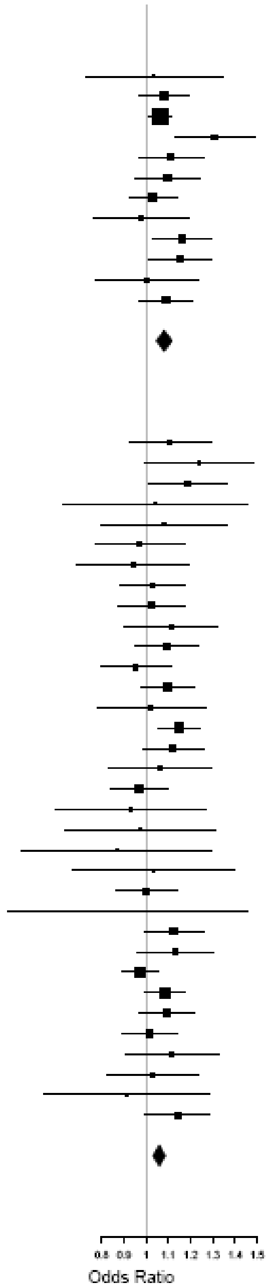

Nature Genetics: doi:10.1038/ng.2480

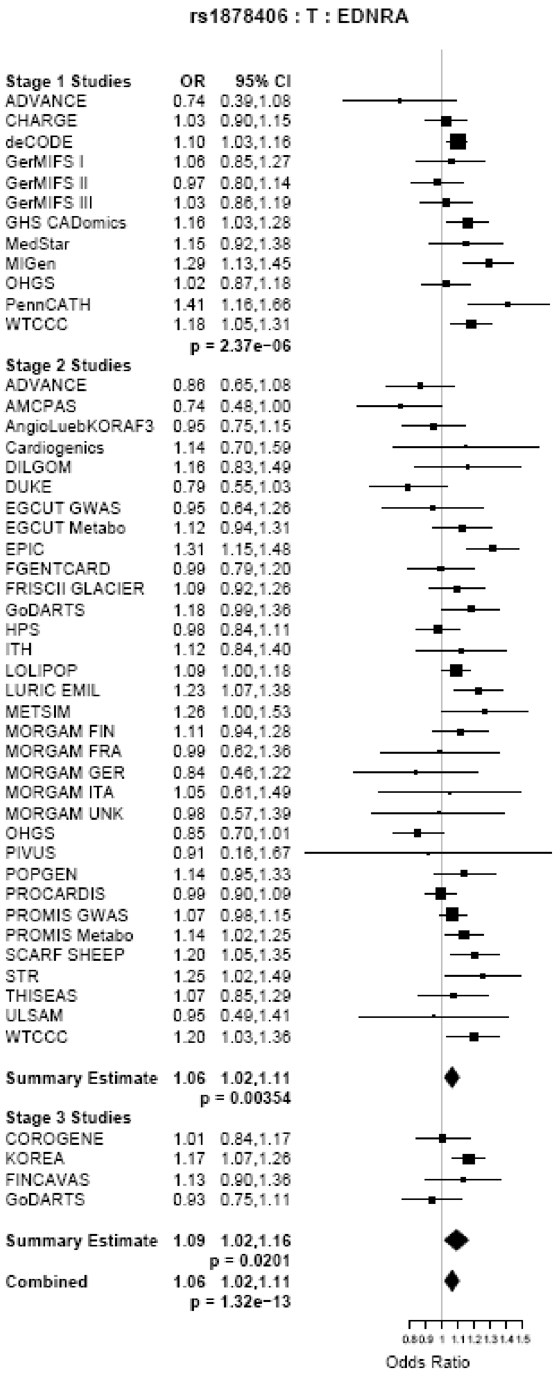

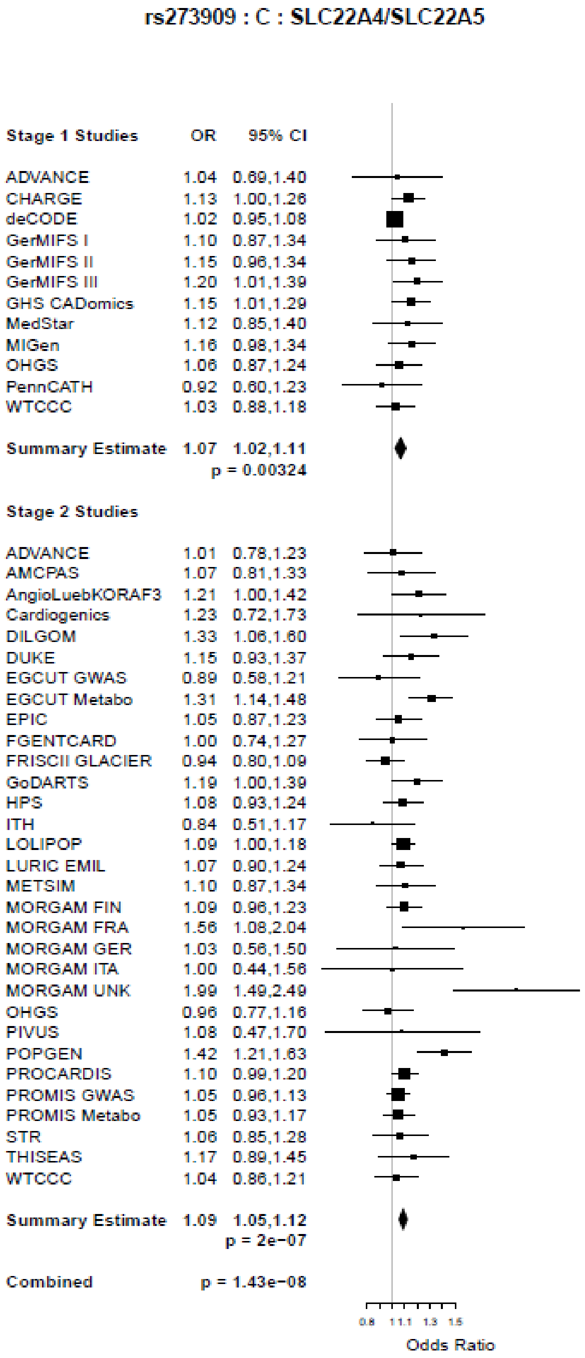

Nature Genetics: doi:10.1038/ng.2480

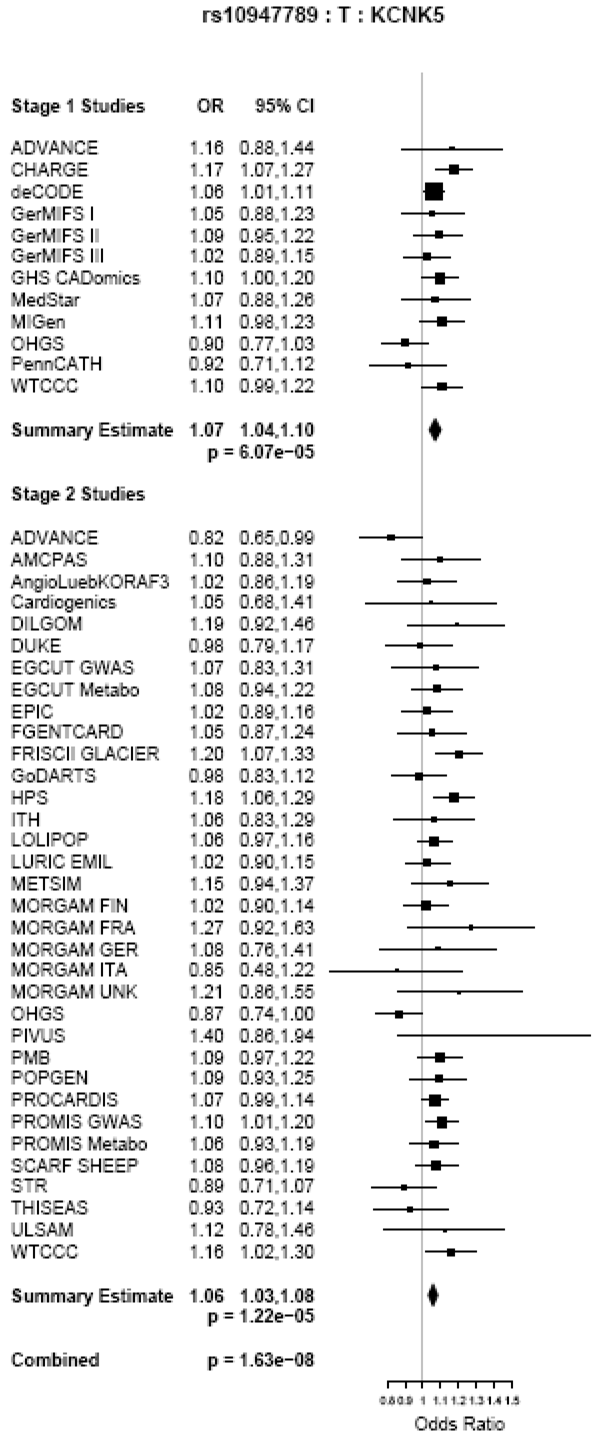

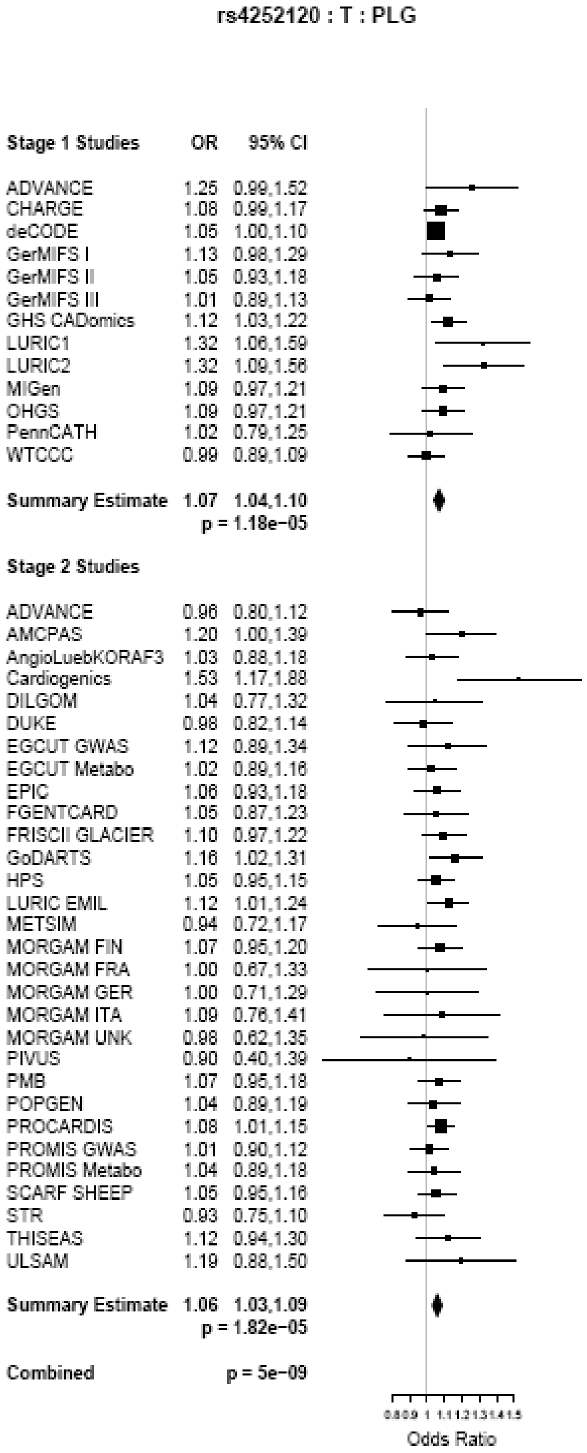

Nature Genetics: doi:10.1038/ng.2480

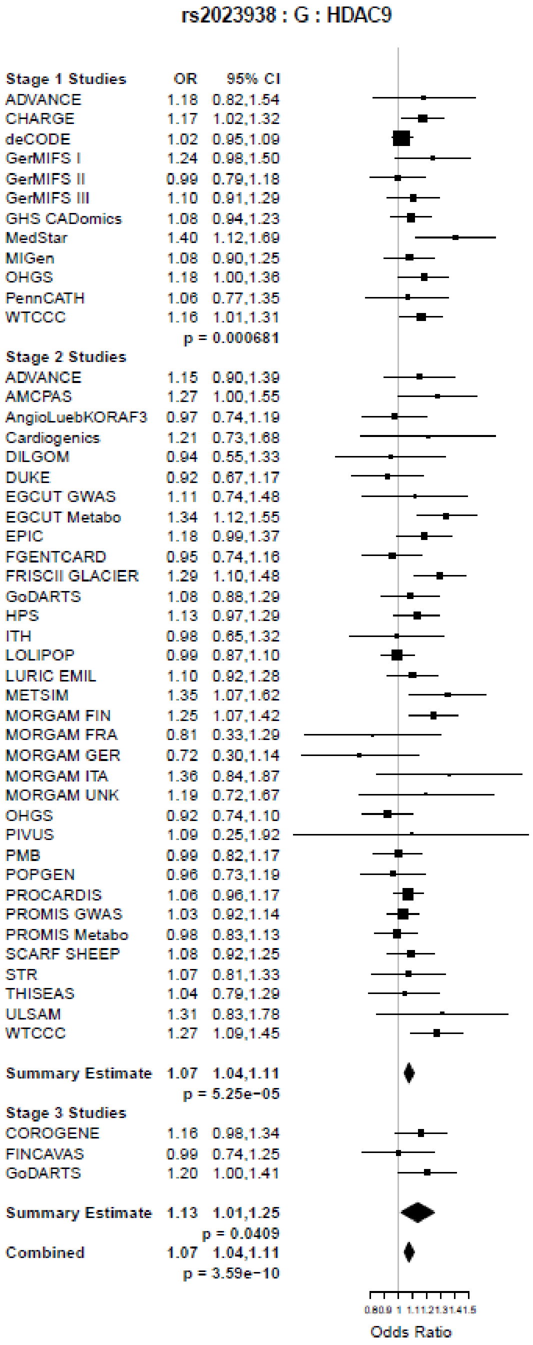

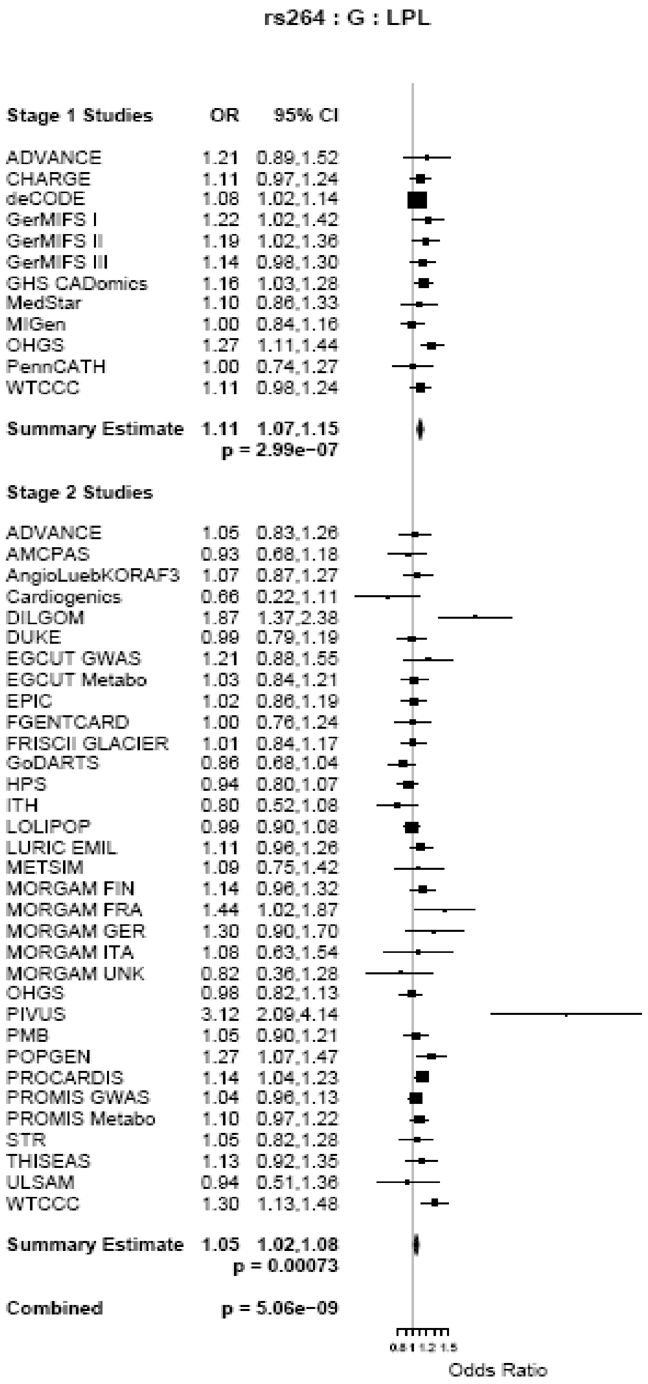

Nature Genetics: doi:10.1038/ng.2480

rs2954029 : A : TRIB1

Stage 1 Studies	OR	95% CI
ADVANCE	1.01	0.77,1.25
CHARGE	1.03	0.95,1.12
deCODE	1.04	0.99,1.08
GerMIFS I	1.26	1.12,1.40
GerMIFS II	0.98	0.87,1.10
GerMIFS III	1.00	0.89,1.11
GHS CADomics	1.08	0.99,1.16
MedStar	1.08	0.91,1.25
MIGen	0.99	0.88,1.09
OHGS	1.20	1.09,1.31
PennCATH	1.08	0.90,1.26
WTCCC	1.18	1.09,1.28
**Summary Estimate**	**1.06**	**1.03,1.09**
		**p = 2.79e−05**
**Stage 2 Studies**		
ADVANCE	1.12	0.97,1.26
AMCPAS	1.12	0.94,1.30
AngioLuebKORAF3	1.05	0.92,1.19
Cardiogenics	1.27	0.95,1.59
DILGOM	1.05	0.80,1.29
DUKE	0.90	0.75,1.04
EGCUT GWAS	1.01	0.81,1.21
EGCUT Metabo	1.04	0.92,1.16
EPIC	1.11	0.99,1.22
FGENTCARD	1.04	0.87,1.20
FRISCII GLACIER	1.11	1.00,1.22
GoDARTS	1.05	0.93,1.18
HPS	1.07	0.97,1.16
ITH	0.88	0.68,1.08
LOLIPOP	1.01	0.94,1.09
LURIC EMIL	1.02	0.91,1.12
METSIM	1.18	0.98,1.38
MORGAM FIN	0.98	0.87,1.09
MORGAM FRA	0.94	0.65,1.23
MORGAM GER	1.02	0.75,1.29
MORGAM ITA	0.80	0.48,1.11
MORGAM UNK	0.91	0.61,1.21
OHGS	0.90	0.79,1.00
PIVUS	0.68	0.22,1.13
PMB	1.04	0.93,1.14
POPGEN	0.87	0.73,1.00
PROCARDIS	1.08	1.01,1.14
PROMIS GWAS	1.06	0.99,1.14
PROMIS Metabo	1.14	1.04,1.24
SCARF SHEEP	1.09	0.99,1.20
STR	1.18	1.02,1.33
THISEAS	1.04	0.88,1.21
ULSAM	0.96	0.68,1.25
WTCCC	1.07	0.96,1.19
**Summary Estimate**	**1.04**	**1.02,1.06**
		**p = 7.75e−05**
**Combined**		**p = 4.53e−08**

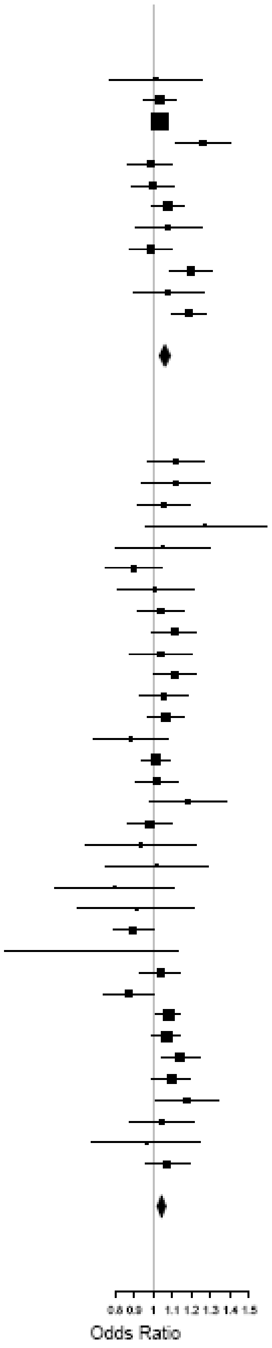

rs17514846 : A : FES

Stage 1 Studies	OR	95% CI
ADVANCE	0.82	0.59,1.05
CHARGE	1.08	0.99,1.17
deCODE	1.06	1.02,1.11
GerMIFS II	1.11	0.99,1.23
GHS CADomics	1.12	1.03,1.21
MedStar	1.07	0.89,1.24
MIGen	1.11	1.00,1.23
PennCATH	0.92	0.73,1.11
**Summary Estimate**	**1.07**	**1.04,1.10**
		**p = 2.37e−05**
**Stage 2 Studies**		
ADVANCE	1.07	0.92,1.21
AMCPAS	1.18	1.01,1.35
AngioLuebKORAF3	0.98	0.84,1.11
Cardiogenics	0.89	0.58,1.20
DILGOM	1.08	0.84,1.33
EGCUT GWAS	1.25	1.05,1.45
EGCUT Metabo	1.00	0.88,1.12
EPIC	1.07	0.96,1.19
FGENTCARD	1.10	0.94,1.26
FRISCII GLACIER	0.97	0.86,1.08
GoDARTS	1.08	0.96,1.21
HPS	1.08	0.98,1.17
ITH	0.88	0.67,1.09
LOLIPOP	1.09	1.01,1.16
LURIC EMIL	1.07	0.97,1.18
METSIM	1.01	0.80,1.21
MORGAM FIN	1.03	0.91,1.14
MORGAM FRA	0.88	0.59,1.18
MORGAM GER	1.03	0.77,1.29
MORGAM ITA	1.11	0.77,1.44
MORGAM UNK	1.81	1.48,2.13
OHGS	0.89	0.78,1.01
PIVUS	0.85	0.42,1.29
PMB	1.03	0.93,1.14
POPGEN	1.02	0.89,1.16
PROCARDIS	1.14	1.08,1.21
PROMIS GWAS	1.02	0.95,1.09
PROMIS Metabo	1.10	1.00,1.20
SCARF SHEEP	1.04	0.94,1.14
STR	0.99	0.83,1.15
THISEAS	1.07	0.91,1.23
ULSAM	1.10	0.81,1.39
WTCCC	1.08	0.97,1.20
**Summary Estimate**	**1.05**	**1.03,1.08**
		**p = 7.35e−07**
**Combined**		**p = 4.49e−10**

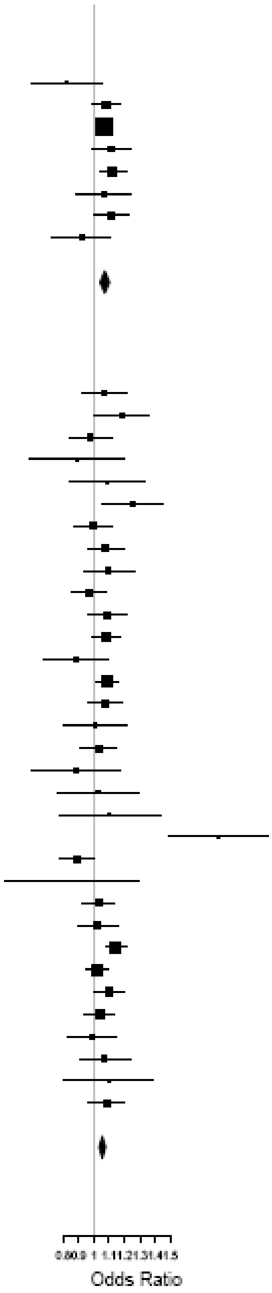

Nature Genetics: doi:10.1038/ng.2480

rs9319428 : A : FLT1

Stage 1 Studies	OR	95% CI
ADVANCE	0.98	0.74,1.22
deCODE	1.04	1.00,1.09
GerMIFS I	0.99	0.84,1.14
GerMIFS II	1.03	0.91,1.16
GerMIFS III	1.13	1.01,1.25
GHS CADomics	1.10	1.01,1.20
LURIC1	1.11	0.85,1.36
MedStar	1.26	1.03,1.48
MIGen	1.09	0.97,1.21
OHGS	1.12	1.00,1.24
PennCATH	1.05	0.85,1.25
WTCCC	1.06	0.96,1.16
**Summary Estimate**	**1.06**	**1.03,1.09**
		**p = 7.88e−05**
**Stage 2 Studies**		
ADVANCE	1.03	0.87,1.19
AMCPAS	0.95	0.76,1.14
AngioLuebKORAF3	1.11	0.96,1.26
Cardiogenics	0.78	0.43,1.13
DILGOM	1.39	1.15,1.64
DUKE	1.08	0.91,1.25
EGCUT GWAS	1.04	0.76,1.31
EGCUT Metabo	1.16	1.03,1.29
EPIC	0.98	0.85,1.10
FGENTCARD	1.04	0.86,1.21
FRISCII GLACIER	1.04	0.92,1.16
GoDARTS	1.03	0.88,1.17
HPS	1.01	0.90,1.11
LOLIPOP	1.06	0.98,1.14
LURIC EMIL	1.02	0.91,1.14
METSIM	0.91	0.70,1.11
MORGAM FIN	1.04	0.93,1.16
MORGAM FRA	0.85	0.55,1.15
MORGAM GER	1.21	0.92,1.50
MORGAM ITA	1.39	1.05,1.73
MORGAM UNK	1.50	1.16,1.85
OHGS	0.92	0.80,1.03
PIVUS	0.83	0.35,1.31
PMB	1.05	0.95,1.16
POPGEN	1.07	0.93,1.22
PROCARDIS	1.11	1.04,1.18
PROMIS GWAS	1.01	0.94,1.09
PROMIS Metabo	1.04	0.94,1.14
SCARF SHEEP	1.17	1.06,1.27
STR	0.99	0.82,1.16
THISEAS	0.97	0.79,1.16
ULSAM	1.47	1.17,1.76
WTCCC	1.12	1.00,1.25
**Summary Estimate**	**1.05**	**1.03,1.08**
		**p = 5.7e−06**
**Combined**		**p = 1.01e−08**

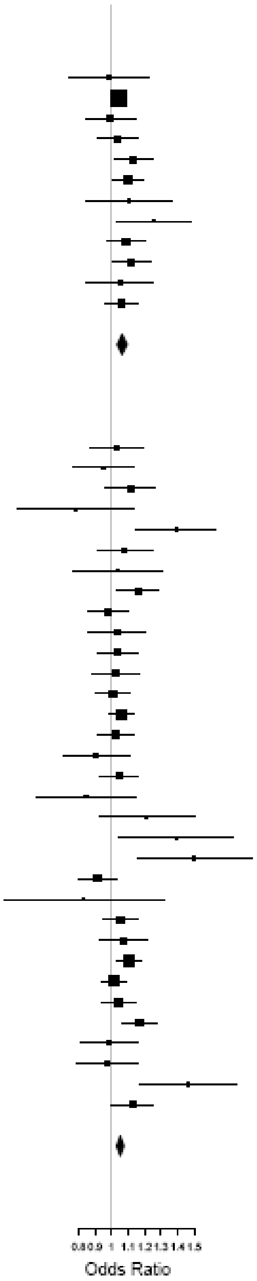

Nature Genetics: doi:10.1038/ng.2480

Supplementary Figure 4: Regional associations plots of the 15 novel CAD loci as well as the locus from the young cases vs. controls subgroup analysis. Each circle represents a SNP from the Stage 1 results; purple diamond is the lead SNP from the Stage 1 and 2 combined analysis; purple circle is the identical lead SNP from the Stage 1 results.

IL6R

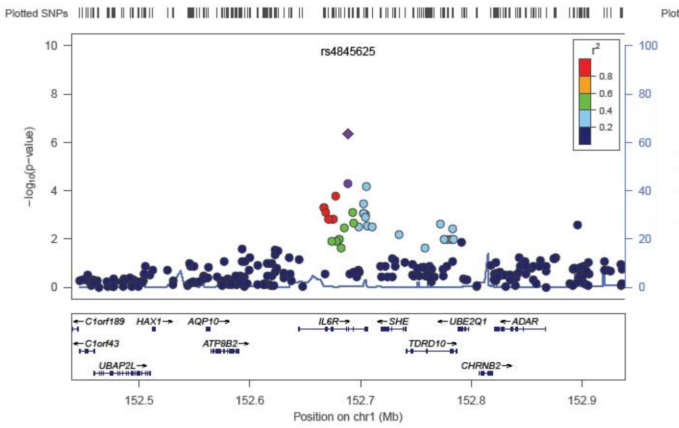

APOB

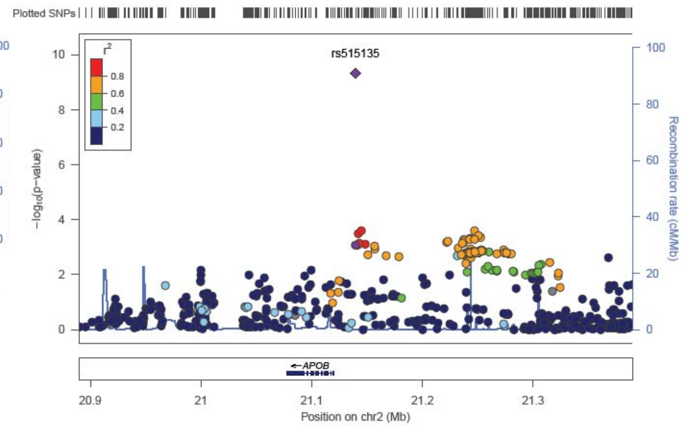

ABCG5/ABCG8

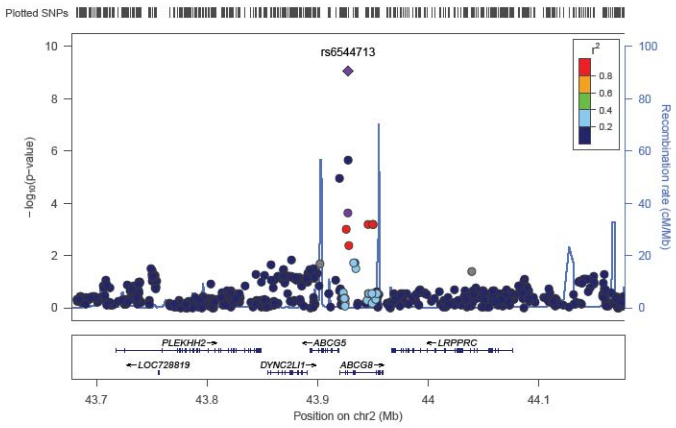

ZEB2-AC074093.1

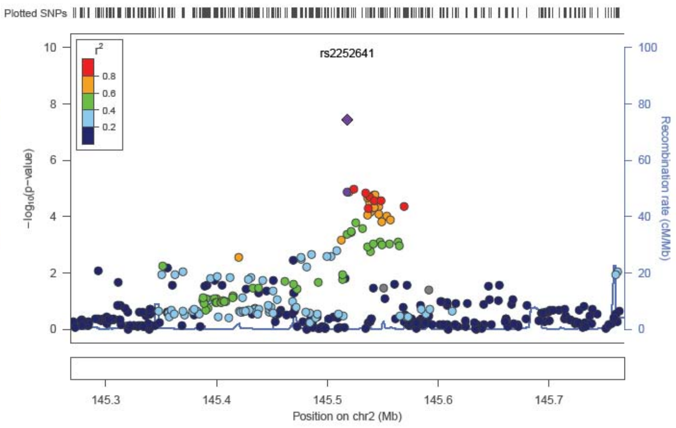

Nature Genetics: doi:10.1038/ng.2480

SLC22A4/SLC22A5

GGCX/VAMP8

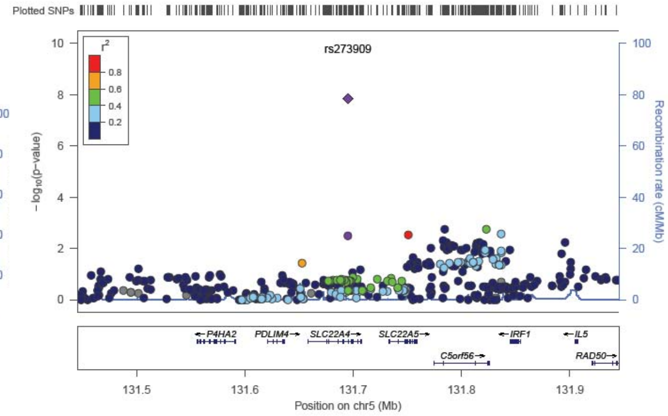

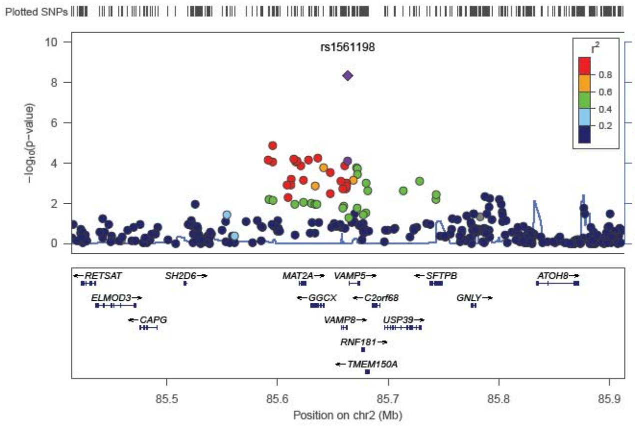

K

CNK5

GUCY1A3

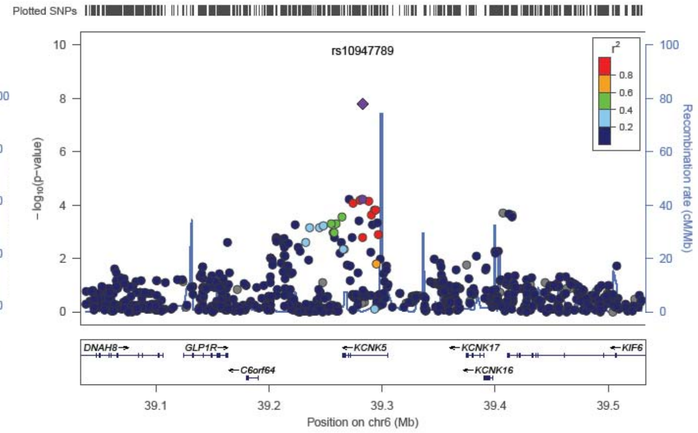

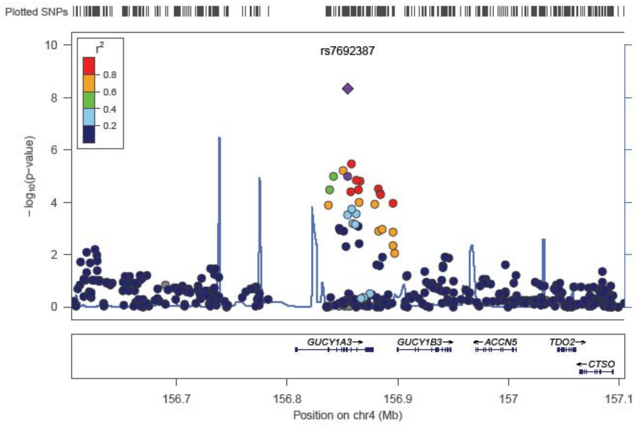

PLG

EDNRA

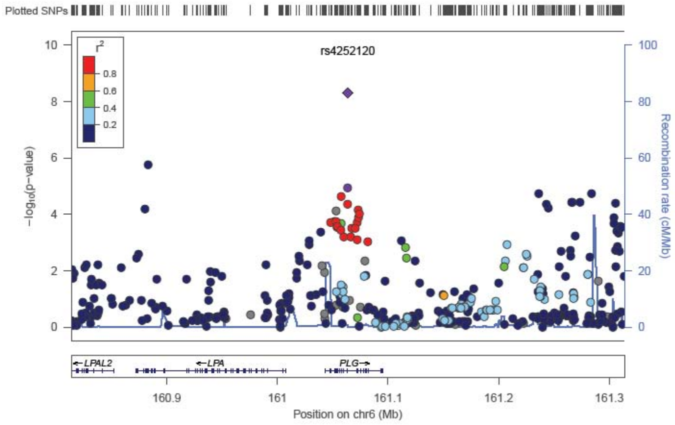

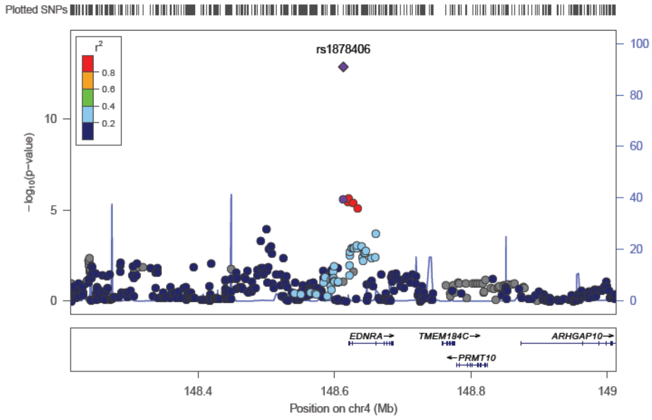

HDAC9

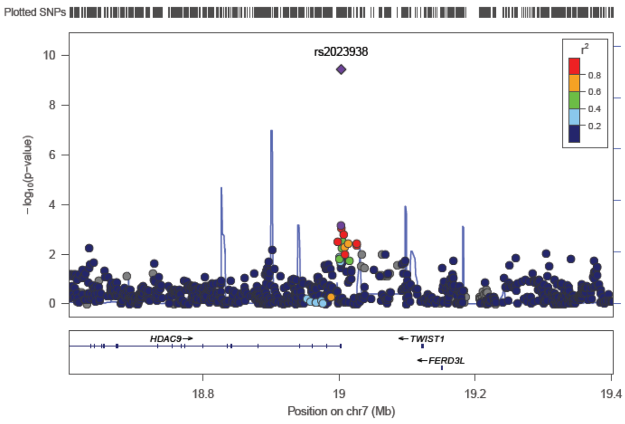

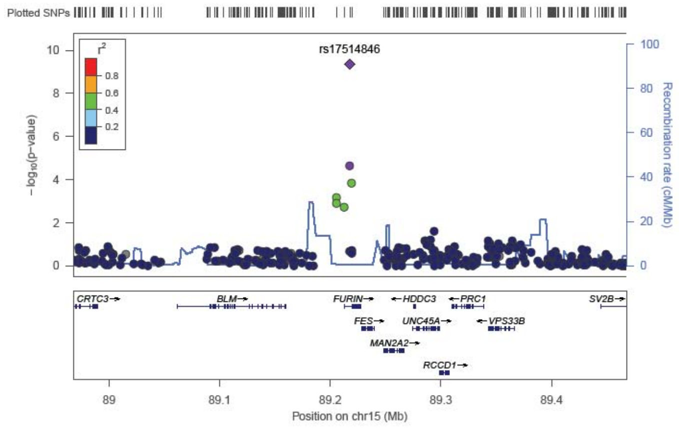

LPL

FLT1

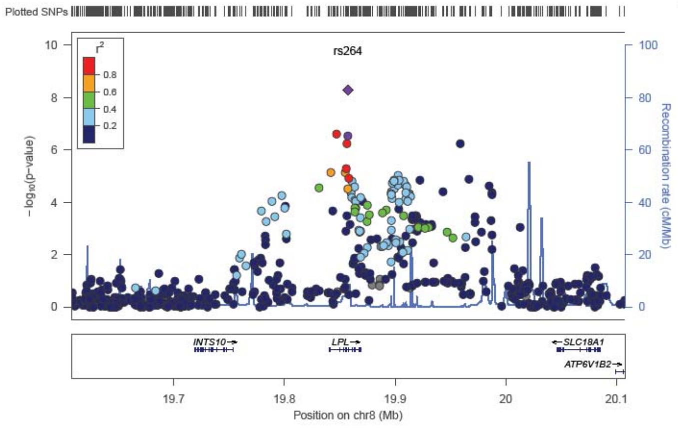

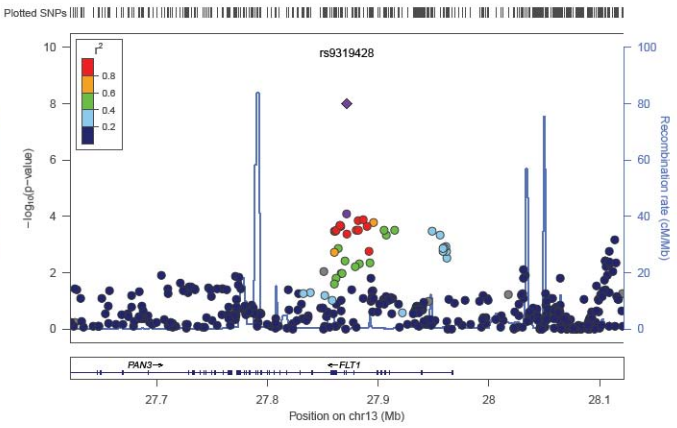

TRIB1

rs16986953

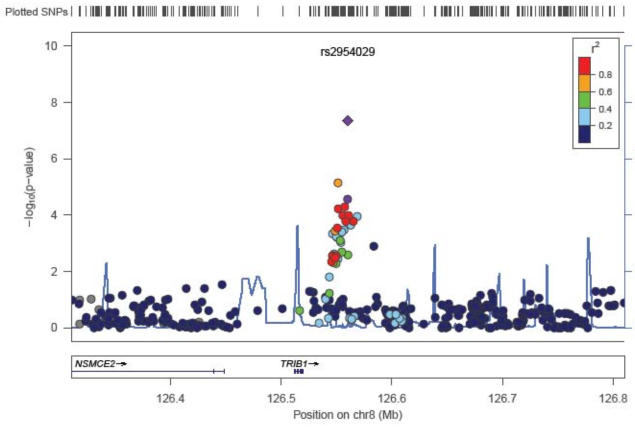

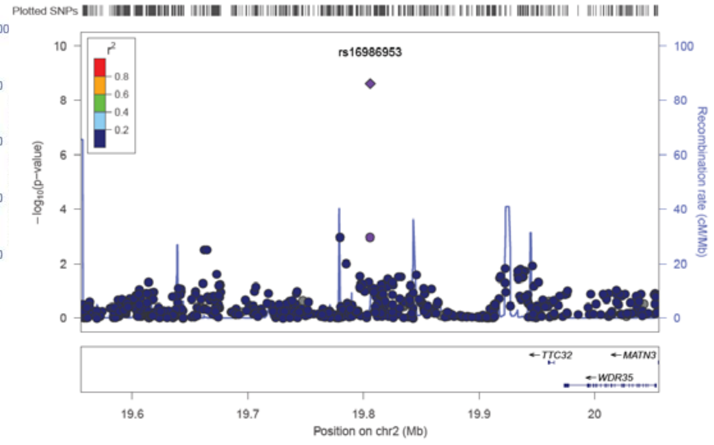

FURIN/FES

Nature Genetics: doi:10.1038/ng.2480

Supplementary Figure 5: Histogram of ORs for the 104 SNPs (LD threshold of $r^2 < 0.2$ and FDR ≤ 5%); SNPs reported in Table 1 and 2 were excluded. The summary descriptive for these ORs are: mean 1.054, SD 0.0197, SEM 0.00187, median 1.047, IQR 0.0199, min 1.031, max 1.126.

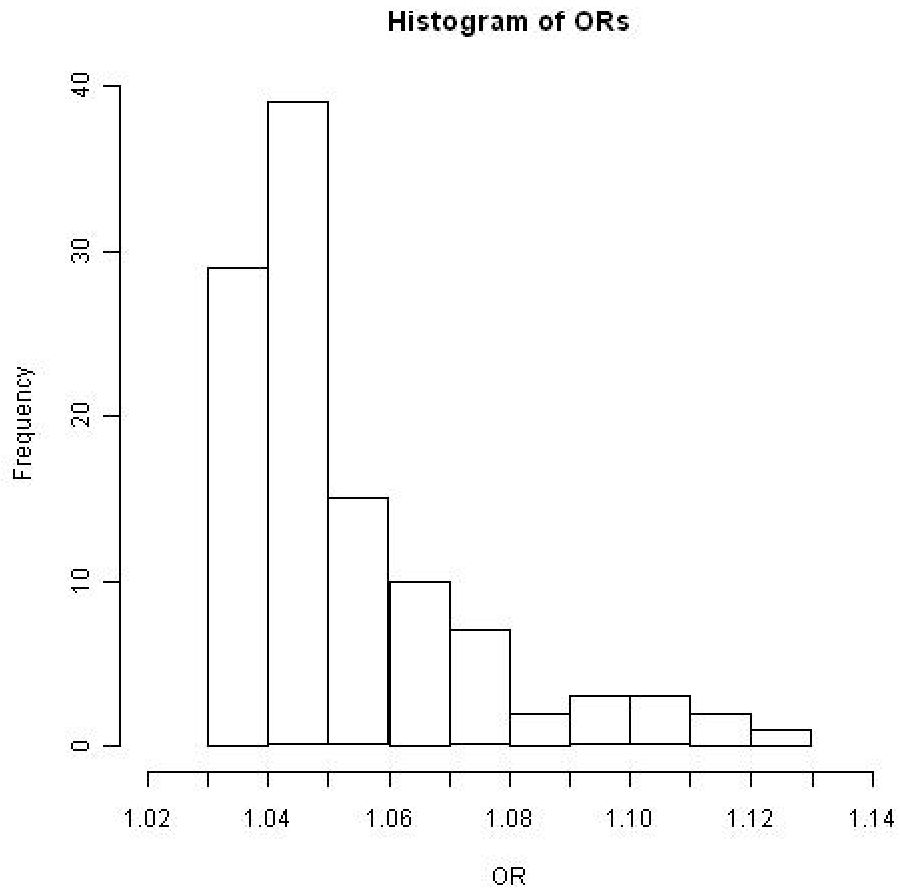

Nature Genetics: doi:10.1038/ng.2480

Supplementary Figure 6: Diagram depicting part of overlapping network ON1 and the interactions recorded in the Ingenuity Knowledge data base between constituent genes. Genes in confirmed CAD loci are marked in red (known) and green (novel) whereas genes selected at FDR 10% are marked in grey. Genes drawn in to the network as neighbours are marked in white. The candidate CAD risk genes shown map to canonical pathways involved in both lipid metabolism and inflammation.

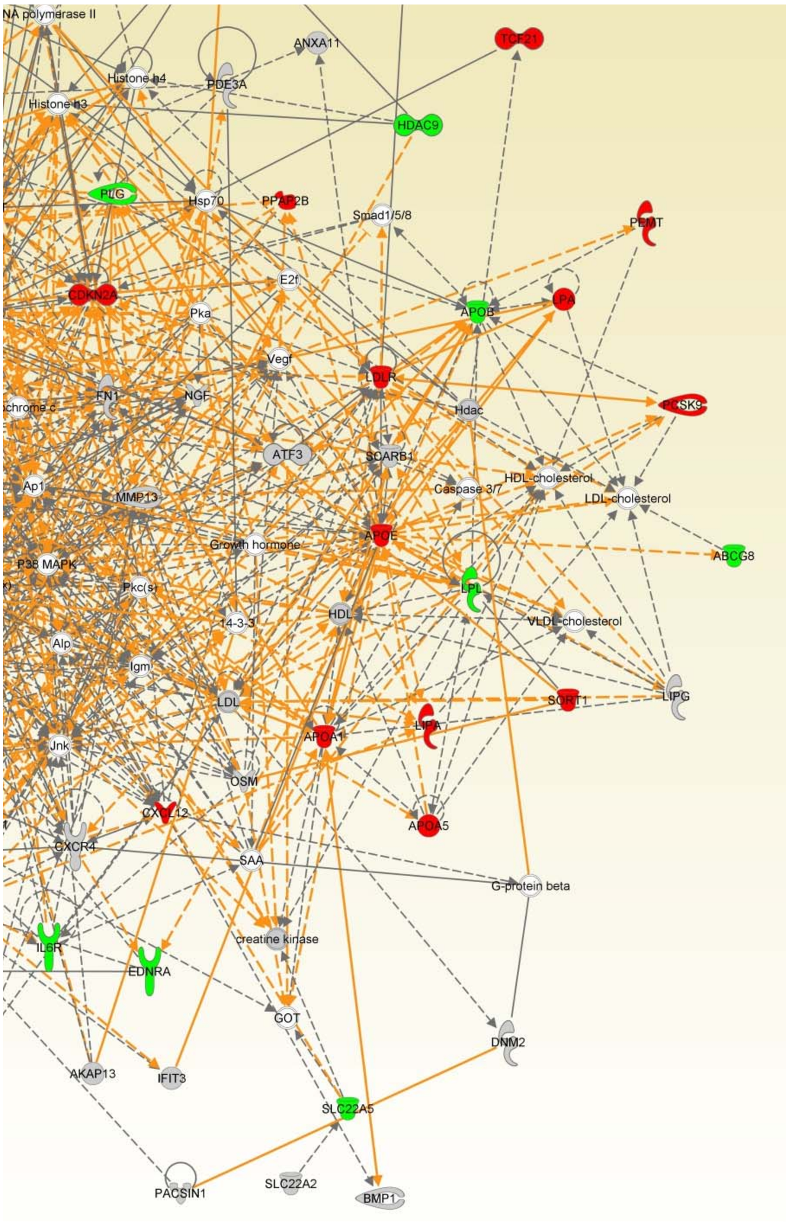

Nature Genetics: doi:10.1038/ng.2480

Supplementary Figure 7: Density histogram of p2_final for the 54,803 SNPs considered in the FDR analysis. The dashed line is the expected distribution if all SNPs were null, the dotted line indicates the $\hat{\pi}_0$ estimate (=0.831).

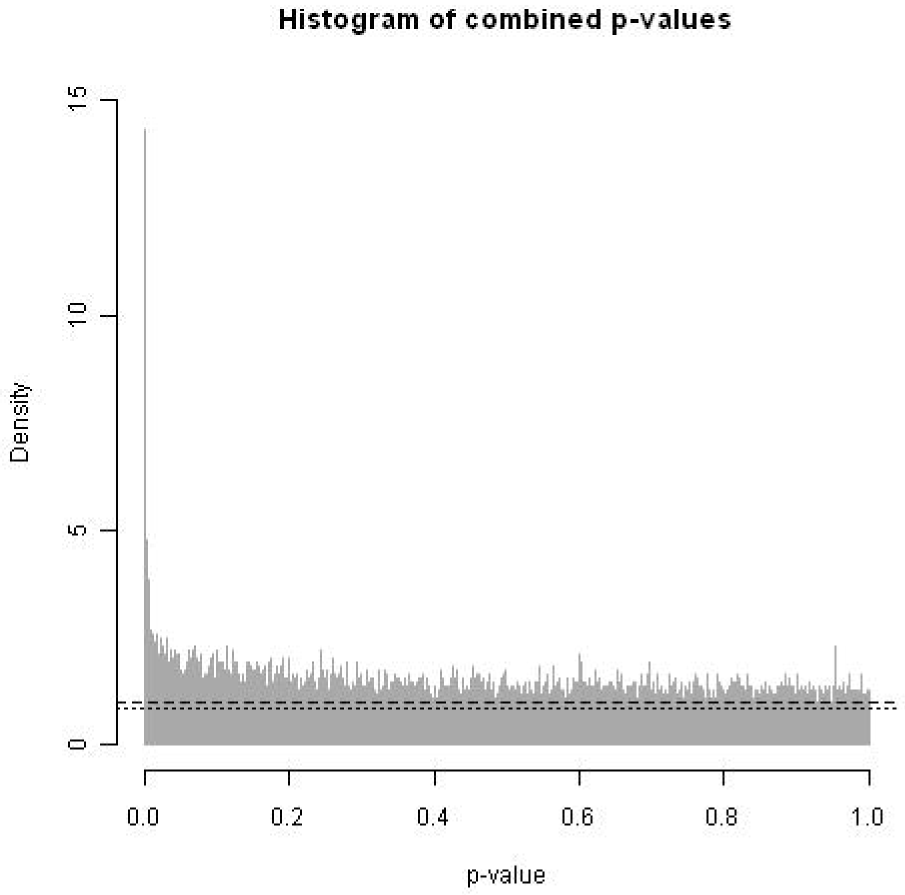

Nature Genetics: doi:10.1038/ng.2480

Tables

Supplementary Table 1a: Description of studies in Stage2

Study	Phenotype	Sample size	Age(years)<br>Mean(SD)	BMI(kg/m^2)<br>Mean(SD)	Sample size<br>MI	Young<br>CAD	Old<br>CAD	Diabetes<br>mellitus	Diagnosed<br>hypertension	Current<br>smokers
		Total study<br>male/female<br>cases/controls				Total study<br>male/female			Total study<br>cases/controls	
ADVANCE	CAD/Mi	1568<br>1099/469<br>900/668	64.0(6.5)<br>63.2(7.0)/66.0(4.8)<br>62.7(8.0)/65.8(2.9)	28.8(5.2)<br>28.9(6.7)/28.5(6.3)<br>29.1(5.2)/28.3(5.2)	600<br>457/143	60<br>59/1	840<br>619/221	237<br>157/80	1094<br>824/270	967<br>569/398
AMC-PAS	CAD/MI	1608<br>1104/504<br>446/1162	48.4(11.4)<br>49.3(10.9)/46.2(12.1)<br>41.6(6.0)/51.0(11.8)	NA	354<br>266/88	440<br>336/104	6<br>4/2	NA	NA	NA
Angio-Lueb<br>/KORAF3	CAD	2658<br>1655/1003<br>1254/1404	53.0(10.6)<br>53.4(9.9)/52.3(11.7)<br>55.0(6.8)/51.1(12.9)	27.7(4.9)<br>28.1(4.4)/26.9(5.5)<br>28.4(5.0)/27.0(4.7)*	NA	252<br>195/57	1003<br>803/200	NA<br>284/NA*	1486<br>947/539*	1533<br>722/811*
Cardiogenics	CAD/MI	776<br>492/284<br>384/392	55.2(8.1)<br>55.8(8.3)/54.3(7.6)<br>57.0(8.8)/53.5(7.0)	27.1(4.3)<br>27.7(4.1)/26.0(4.4)<br>28.4(4.4)/25.7(3.7)	53<br>47/6	76<br>70/6	308<br>262/46	NA	NA	37<br>11/26
DILGOM	CAD/MI	3991<br>1820/2171<br>147/3844	52.1(13.6)<br>53.0(13.5)/51.4(13.6)<br>56.6(9.5)/51.7(13.6)	NA	88<br>69/19	34<br>29/5	113<br>87/26	352<br>51/301	2192<br>112/2080	690<br>20/670
DUKE	CAD/MI	1848<br>1105/743<br>1200/648	5.7(9.9)<br>5.7(9.8)/6.2(9.2)<br>5.7(9.7)/6.3(8.7)	28.9(6.6)<br>29.2(6.4)/28.5(6.9)<br>29.2(6.3)/28.4(7.2)	577<br>457/120	304<br>256/48	896<br>577/319	NA	NA	NA
EGCUT<br>GWAS	CAD/MI	2826<br>1168/1635<br>313/2513	45.9(20.8)<br>39.4(16.2)/50.6(22.4)<br>69.8(13.8)/43.0(19.6)	26.1(6.6)<br>26.1(7.6)/26.1(5.8)<br>28.4(5.0)/25.8(6.7)	39<br>23/16	21<br>15/6	281<br>75/206	139<br>47/92	733<br>213/520	453<br>25/428
EGCUT<br>Metabochip	CAD/MI	2626<br>1039/1587<br>983/1643	58.2(11.8)<br>58.6(11.1)/58.0(12.3)<br>61.7(10.8)/56.2(11.8)	28.2(6.5)<br>28.0(5.7)/28.3(6.9)<br>30.1(6.7)/26.9(6.7)	228<br>148/80	122<br>77/45	861<br>400/461	934<br>301/633	901<br>469/432	617<br>186/431
EPIC	CAD/MI	3935<br>2069/1866<br>1526/2409	64.7(10.5)<br>65.6(10.2)/63.7(10.8)<br>71.8(8.18)/60.3(9.3)	27.0(4.2)<br>27.0(3.6)/26.9(4.8)<br>27.1(3.8)/26.9(4.4)	1526<br>990/536	7<br>6/1	1519<br>984/535	NA	NA	NA
FGENTCAR<br>D	CAD/MI	1988<br>433/1554<br>1435/553	61.9(11.4)<br>60.3(11.6)/63.2(10.7)<br>61.0(11.1)/55.6(11.6)	29.1(4.7)<br>28.6(4.6)/30.5(6.0)<br>29.1(5.0)/29.6(5.5)	796<br>614/182	733<br>548/185	3910<br>2762/1148	1992<br>1787/205	3895<br>3306/589	2610<br>2189/421
FRISCII-<br>GLACIER	CAD/MI	9247<br>4640/4607<br>2937/6310	55.5(12.0)<br>57.5(11.8)/53.5(11.9)<br>66.2(9.8)/50.5(9.3)	NA	1074<br>792/282	191<br>177/14	2746<br>1883/863	NA	NA	NA
GLACIER**<br>*	Control	5875<br>2291/3584	49.4(8.8)<br>49.9(8.4)/49.2(8.2)	25.8(4.0)<br>25.9(3.4)/25.7(4.0)	NA	NA	NA	0	1980	1342
GoDARTS	CAD/MI	2765<br>1634/1131<br>619/2146	61.7(9.8)<br>61.2(9.7)/62.5(9.7)<br>61.5(10.5)/61.8(9.5)	31.8(6.3)<br>31.0(5.5)/33.0(7.1)<br>30.0(5.7)/32.3(6.3)	516<br>366/150	81<br>63/18	538<br>377/161	1188<br>250/938	1227<br>319/908	1789<br>338/1451

Nature Genetics: doi:10.1038/ng.2480

HPS	CAD/MI	5457<br>3069/2388<br>2700/2757	54.8(13.2)<br>55.5(11.4)/53.8(15.2)<br>59.3(8.5)/50.3(15.4)	27.5(4.2)<br>27.3(3.9)/28.3(5.2)**	1754<br>1490/264	370<br>314/56	NA	375**	1171**	376**
ITH	CAD/MI	815<br>562/253<br>388/427	63.8(10.5)<br>63.2(10.6)/65.2(10.1)<br>63.3(10.6)/64.3(10.3)	27.2(10.4)<br>26.7(5.7)/28.3(16.5)<br>26.6(7.1)/27.7(12.6)	388<br>273/115	36<br>30/6	352<br>243/109	116<br>70/46	401<br>207/194	NA
LOLIPOP	CAD/MI	6557<br>5527/1030<br>2797/3760	55.4(10.6)<br>55.1(10.7)/56.8(10.0)<br>59.3(9.7)/52.4(10.2)	27.1(4.3)<br>26.8(4.1)/28.7(5.3)<br>27.6(4.5)/26.8(4.2)	1228<br>1122/106	493<br>427/66	2304<br>1862/442	1782<br>1122/660	4090<br>2186/1904	623<br>236/387
LURIC-<br>EMIL	CAD/MI	4560<br>2769/1791<br>2068/2492	56.4(13.6)<br>57.1(13.1)/55.3(14.3)<br>58.9(13.0)/49.4(12.9)	NA	1230<br>964/266	210<br>172/38	1858<br>1386/472	NA	NA	NA
METSIM	CAD/MI	2119<br>2119/0<br>224/1895	59.4(7.4)<br>59.4(7.4)/NA(NA)<br>64.6(6.3)/58.7(7.3)	28.5(4.8)<br>28.5(4.8)/NA(NA)<br>30.2(6.4)/28.3(4.5)	109<br>109/0	35<br>35/0	168<br>168/0	99<br>18/81	NA	19<br>1/18
MORGAM-<br>FIN	CAD	2484<br>2118/366<br>1242/1242	62.7(7.74)<br>62.6(7.7)/63.0(8.2)<br>64.5(7.3)/60.9(7.8)	27.7(4.4)<br>27.5(4.2)/28.8(5.1)<br>28.1(4.3)/27.3(4.3)	32<br>24/8	NA	1192<br>1015/177	214<br>149/65	1473<br>771/702	1286<br>644/642
MORGAM-<br>FRA	CAD/MI	366<br>366/0<br>183/183	57.0(3.0)<br>57.0(3.0)/NA(NA)<br>57.6(3.0)/56.3(2.7)	27.3(3.3)<br>27.3(3.3)/NA(NA)<br>27.6(3.2)/27.0(3.5)	50<br>50/0	NA	183<br>183/0	24<br>12/12	202<br>116/86	101<br>63/38
MORGAM-<br>GER	CAD/MI	430<br>340/90<br>215/215	61.7(8.5)<br>61.6(8.5)/62.3(8.4)<br>64.5(7.8)/58.9(8.2)	28.0(4.0)<br>27.8(3.6)/28.5(5.0)<br>28.3(4.4)/27.6(3.5)	85<br>67/18	14<br>13/1	201<br>157/44	54<br>37/17	247<br>131/116	159<br>99/60
MORGAM-<br>ITA	CAD/MI	302<br>242/60<br>151/151	58.4(9.2)<br>57.3(9.3)/62.8(7.3)<br>61.3(9.3)/55.5(8.1)	26.4(3.9)<br>26.2(3.4)/27.3(5.5)<br>26.7(4.0)/26.0(3.9)	62<br>52/10	25<br>23/2	126<br>98/28	36<br>23/13	163<br>94/69	114<br>68/46
MORGAM-<br>UNK	CAD/MI	328<br>328/0<br>164/164	57.9(4.0)<br>57.9(4.0)/NA(NA)<br>59.7(4.1)/56.1(3.0)	26.4(3.4)<br>26.4(3.4)/NA(NA)<br>26.9(3.3)/26.0(3.4)	65<br>65/0	NA	164<br>164/0	14<br>9/5	150<br>87/63	129<br>74/55
OHGS	CAD/MI	3419<br>2212/1207<br>1998/1421	59.6(14.2)<br>54.5(13.5)/67.2(12.3)<br>49.0(7.3)/74.5(5.5)	28.1(5.3)<br>28.5(4.9)/27.4(5.8)<br>29.1(5.5)/26.7(4.6)	1200<br>963/237	997<br>877/120	1001<br>637/364	72<br>0/72	1718<br>1147/571	434<br>399/35
PIVUS	CAD	978<br>490/488<br>94/884	69.7(2.7)<br>69.3(3.5)/70.1(1.5)<br>65.0(7.2)/70.2(0.2)	27.1(4.4)<br>27.1(3.7)/27.1(4.9)<br>27.7(4.3)/27.0(4.4)	94<br>74/20	NA	NA	84<br>16/68	701<br>73/628	105<br>15/90
PMB	CAD/MI	5381<br>2920/2461<br>922/4459	57.6(10.7)/58.4(10.1)<br>59.7(10.8)/57.6(10.3)	27.5(5.6)/27.4(5.6)<br>29.1(5.1)/27.1(5.7)	922<br>542/380	738<br>386/352	184<br>156/28	3238<br>922/2316	501<br>238/263	491<br>183/308
PopGEN	CAD	1836<br>1276/560<br>865/971	54.1(11.7)<br>53.8(10.4)/54.9(14.3)<br>53.5(5.7)/54.7(15.2)	NA	NA	198<br>160/38	667<br>554/113	NA	NA	NA
PRO-<br>CARDIS	CAD/MI	12237<br>6967/5270<br>5719/6518	57.5(11.6)<br>56.2(11.1)/59.2(12.1)<br>53.6(8.1)/60.9(13.1)	28.1(4.5)<br>28(4.1)/28.2(5.5)<br>28.5(4.5)/26.4(4.0)	4598<br>3589/1009	1680<br>1398/282	4039<br>2887/1152	925<br>870/55	1319<br>961/358	1102<br>864/238
PROMIS<br>GWAS	CAD/MI	7353<br>5948/1405<br>3729/3624	53.8(10.3)<br>53.4(10.4)/55.3(9.6)<br>54.2(10.6)/53.5(10.0)	25.3(4.4)<br>25.1(4.2)/25.9(4.8)<br>25.2(4.4)/25.4(4.4)	3729<br>3099/630	1171<br>1036/135	2558<br>2063/495	1839<br>1080/759	2108<br>1080/1028	2096<br>1166/930
PROMIS<br>Metabochip	CAD/MI	3625<br>2965/660<br>1852/1773	52.5(9.9)<br>52.1(10.1)/54.0(9.0)<br>52.9(10.5)/51.9(9.3)	26.1(4.3)<br>25.9(4.2)/26.7(4.7)<br>26.0(4.1)/26.1(4.4)	1851<br>1569/282	648<br>590/58	1204<br>979/225	574<br>363/211	NA	NA
SCARF-<br>SHEEP	CAD/MI	3417<br>2458/959<br>1525/1892	58.1(7.3)<br>57.2(7.1)/60.5(7.2)<br>57.6(7.3)/50.5(7.0)	26.1(4.8)<br>26.1(4.5)/25.9(5.4)<br>26.7(5.1)/25.6(4.4)	1525<br>1122/403	302<br>256/52	1223<br>872/351	341<br>237/104	1467<br>658/809	1133<br>620/513

Nature Genetics: doi:10.1038/ng.2480

STR	CAD	1719<br>760/959<br>447/1272	75.6(10.9)<br>74.4(10.4)/76.5(11.2)<br>78.9(9.7)/73.1(11.0)	25.0(3.9)<br>25.0(3.3)/25.0(4.2)<br>25.5(3.78)/24.9(3.9)	447<br>253/194	NA	NA	77<br>30/47	764<br>249/515	122<br>36/86
THISEAS	CAD/MI	1506<br>871/635<br>508/998	58.7(13.1)<br>57.2(12.3)/60.8(13.9)<br>61.1(10.4)/57.5(14.2)	28.7(6.7)<br>28.7(7.6)/28.5(5.3)<br>28.3(9.6)/28.8(5.3)	262<br>216/46	88<br>79/9	420<br>347/73	310<br>140/170	643<br>256/387	360<br>161/199
ULSAM	CAD	1175<br>1175/0<br>242/933	71.8(5.6)<br>71.8(5.6)/NA(NA)<br>72.5(8.7)/71.2(0.4)	26.0(3.2)<br>26.0(3.2)/NA(NA)<br>26.7(3.8)/25.9(3.0)	242<br>242/0	NA	NA	45<br>18/27	338<br>75/263	222<br>50/172
WTCCC CAD2	CAD/MI	5429<br>3347/2082<br>1213/4246	46.7(6.7)/45.2(4.6)<br>53.5(9.6)/44(0)	28.1(4.2)/27.0(5.6)<br>28.7(4.9)/27.4(4.7)	1124<br>914/210	435<br>366/69	778<br>613/165	301<br>116/185	1560<br>473/1087	1373<br>407/966

* Descriptive information only available for some individuals within the cohort
** Descriptive information only available for the cases within the cohort

***Results for the control cohort GLACIER presented separately, Total and Male/Female data descriptives.

Supplementary Table 1b: Description of studies in Stage3

Study	Phenotype	Sample size	Age(years) Mean(SD)	BMI(kg/m^2) Mean(SD)	Sample size: MI	Young CAD	Old CAD	Diabetes mellitus	Diagnosed hypertension	Current smokers
		Total study<br>male/female<br>cases/controls			Total study<br>male/female			Total study<br>cases/controls		
COROGENE	CAD	3208<br>2079/1129<br>2000/1208	62.72 (12.48)<br>61.25(12.53)/65.44 (11.93)<br>66.0 (11.8) / 56.74 (11.29)	27.32 (4.59)<br>27.49(4.25)/27.02(5.14)<br>27.51(4.72)/27.02(4.34)	200<br>172/28	178<br>155/23	1822<br>1244/578	468<br>397/71	2075<br>1374/701	978<br>659/319
FINCAVAS	CAD/MI	1489<br>997/492<br>798/691	58.1 (10.7)<br>57.8 (10.6)/58.6 (10.9)<br>60.0 (10.2)/55.8 (10.8)	27.7 (4.6)<br>27.7 (4.3)/27.6 (5.0)<br>27.9 (4.5)/27.5 (4.6)	99<br>85/14	128<br>107/21	670<br>518/152	184<br>116/68	NA	373<br>222/151
GenRIC	CAD/MI	4789<br>2935,1854<br>2099,2690	52.8 (8.1)<br>51.8 (8.4)/ 54.4 (7.5)<br>51.7 (7.5)/ 53.7 (8.5)	24.6 (3.0)<br>24.7 (2.9)/ 24.3 (3.1)<br>25.3 (3.0)/ 24.0 (2.8)]	680<br>521/ 159	792<br>716/ 76	1,307<br>568/ 739	653<br>449/ 204	None	1,154<br>653/ 501

[2] Illumina Cardio-Metabochip

Nature Genetics: doi:10.1038/ng.2480

Supplementary Table 2a: General Study Characteristics for Stage 2 studies

Study	Full Study name	Reference	Ethnicity	Region of recruitment	Phenotype	Phenotype definition	Control definition
ADVANCE	Atherosclerotic Disease, VAscular FuNction, & GenetiC Epidemiology study	16490908 16840522 17084253 18443000	European	Northern California, USA	CAD/MI	Kaiser Permanente of Northern California (KPNC) Medical Care Program members aged 45 years or older for males and 55 years or older for females at the time of their incident clinical coronary artery disease event between 28 October 2001 and 31 December 2003. For MI, patients had to have positive cardiac enzymes in the electronic databases as well as a primary discharge diagnosis of myocardial infarction (ICD 9 code 410). For stable angina, patients had to have diagnosis of stable angina (ICD9 code 413.x) in the electronic outpatient databases followed by confirmation from both the primary care physician and the patient of the recent onset of incident stable and typical angina. Two cases from the early onset CAD cohort (age of onset of CAD < 45 years for men and < 55 years for women) not included in prior ADVANCE GWAS were also genotyped.	Members of the KPNC Medical Care Program aged 60 to 69 as of January 6, 2001, with no history of cardiovascular disease, cancer (other than nonmelanoma skin cancer), renal failure, liver cirrhosis, dementia, or human immunodeficiency virus/ acquired immunodeficiency syndrome or with a source of care greater than 50 miles (80.47 km) from the clinic used for data collection were recruited.
AMC-PAS	The Academic Medical Center Amsterdam Premature Atherosclerosis Study	19164808	European	The Netherlands	CAD/MI	Symptomatic CAD before the age of 51 years, defined as MI, coronary revascularization, or evidence of at least 70% stenosis in a major epicardial artery	Blood donors from the north-west region of the Netherlands; recruited at routine Sanquin Blood Bank donation sessions. More than 95% of the controls are from the same region as the cases of the AMC-PAS cohort.
Angio-Lueb/ KORA F3	Lübeck Registry of Structural Heart Disease /KORA (Kooperative Gesundheitsforschung	21378990 16648850	European	Germany	CAD/MI	Consecutive patients referred for coronary angiography, classified as CAD/MI cases based on the coronary angiogram; CAD < 65 y in males, CAD < 70 y in females	Population based controls conducted between 1994 and 2004

Nature Genetics: doi:10.1038/ng.2480

	in der Region Augsburg) survey S3/F3						
Cardiogenics	Cardiogenics Study	17634449	European	France, Germany, England	CAD/MI	Patients from Germany and England were under the age of 65 with a confirmed primary MI within the preceding 3-36 months. Exclusion criteria were (*i*) a history of diabetes mellitus based on plasma glucose >7.0 mmol/l or HbA1C > 7.0 (*ii*) renal insufficiency, (*iii*) patients not on statin therapy, (*iv*) CRP level >10mg/dl, (v) patients not fasting at the time of blood sampling or (*vi*) current smokers. The Paris cohort comprised patients aged 33 to 87, recruited within the BAAAC (Banque d'ADN et d'ARN de patients présentant une Athérosclérose Coronarienne) study. with symptoms of acute coronary syndrome who had one stenosis >50% diagnosed in at least one major coronary artery.	Healthy individuals (aged 32 to 65 years) recruited in Cambridge who were blood donors recruited as part of the Cambridge Bioresource.
DILGOM	The Dietary, Lifestyle, and Genetic determinants of Obesity and Metabolic syndrome study	21179014	European	Finland	CAD/MI	All CAD: Incident definite or possible MI or coronary death, or unstable angina during follow-up, Coronary revascularization during follow-up, Documented MI at baseline, or an unclassifiable coronary death during follow-up. MI:Definite myocardial infarction.	Non-cases from the same population-based longitudinal cohort study
DUKE	The Duke Cathgen Study	20173117	European	United States	CAD/MI	Cases had at least one epicardial coronary vessel with at least 50% blockage. Age of onset was no older than 65 for women and 55 for men. Subjects (case and control), were excluded if they had severe pulmonary hypertension or congenital heart disease or were diabetic.	Controls were required to have no epicardial coronary vessel with greater than 30% blockage. Controls with a history of ICC/PCI, CABG, MI or transplant were excluded. Controls were required to be at least 50 years old.
EGCUT*	Estonian Genome Center of University of Tartu	19424496	European	Estonia	CAD/MI	Cases were study participants who reported following cardiovascular disease events (ICD10 I20-I26) when recruited.	Controls were study participants who didn't report following cardiovascular disease events (ICD10 I20-

Nature Genetics: doi:10.1038/ng.2480

							I26) when recruited.
EPIC	The European Prospective Investigation into Cancer		European	England	CAD/MI	Cases were individuals who developed a fatal or non-fatal CAD during an average follow-up of 11 years, until June 2006. Participants were identified if they had a hospital admission and/or died with CAD as the underlying cause. CAD was defined as cause of death codes ICD9 410-414 or ICD10 I20-I25, and hospital discharge codes ICD10 I20.0, I21, I22 or I23 according to the International Classification of Diseases, 9th and 10th revisions.	Controls were study participants who remained free of any cardiovascular disease during follow-up (defined as ICD9 401-448 and ICD10 I10-I79). Controls were matched to each case by sex, age (within 5 years), and time of enrolment (within 3 months).
FGENTCARD	Functional Genomic Diagnostic Tools for Coronary Artery Disease	In preparation	European	Lebanon	CAD/MI	The study subjects consisted of 6517 individuals who underwent cardiac catheterization following a single consistent and stringent recruitment protocol between August, 2007 and March 2011 at several hospitals in Lebanon. Catheterization was prompted for myocardial infarction (MI) (12.5%) as diagnosed by electrocardiogram and high troponin levels, unstable angina (27.5%), or other reasons, such as stable angina, or heart failure, or reversible ischemia by stress testing (59.9%). All patients underwent coronary catheterization by Judkins technique. The four main coronary arteries: the left main artery (LMCA), the left anterior descending artery (LAD), the left circumflex artery (LCx), and the right coronary artery (RCA) were visualized from different angles by angiography. The extent of stenosis in these vessels was assessed and recorded by percentage. Cases were defined as follows: Mildly diseased if at least one of the four vessels has less than 50% stenosis, severly diseased if any of the four vessels has ≥50% setnosis.	Controls are subjects with no stenosis in the 4 main vessels .
FRISCII	Fragmin and Fast Revascularization	10475181	European	Sweden, Norway and	CAD/MI	FRISCII patients were eligible for inclusion if they had symptoms of	The GLACIER cohort was used in these analyses as a

Nature Genetics: doi:10.1038/ng.2480

GLACIER	during Instability in Coronary Artery Disease (FRISCII) Gene x Lifestyle interactions And Complex traits Involved in Elevated disease Risk (GLACIER)	20870969	European	Denmark Sweden	Control	ischaemia that were increasing or occurring at rest, or that warranted the suspicion of acute myocardial infarction, with the last episode within 48 h before the start of dalteparin or standard heparin treatment. Myocardial ischaemia had to be verified by electrocardiography (ST depression ≥0·1 mV or T-wave inversion ≥0·1 mV) or by raised biochemical markers (creatine kinase [CK]-MB >6 ug/L, troponin-T >0·10 ug/L, qualitative troponin-T test positive, or catalytic activity of CK, CK-B, or CK MB higher than the local diagnostic limit for myocardial infarction). Exclusion criteria were raised risk of bleeding episodes, anaemia, or indication for or treatment in the past 24 h with thrombolysis, angioplasty in the past 6 months, being on a waiting list for coronary revascularisation, other acute or severe cardiac disease, renal or hepatic insufficiency, known clinically relevant osteoporosis, other severe illness, hypersensitivity to randomised drugs, anticipated difficulties with cooperation or participation in this or another clinical trial. Patients with previous open-heart surgery, advanced age (eg, >75 years), or other disorders that made randomisation to early revascularisation inappropriate.	comparison cohort for FRISCII. GLACIER is a subset of the Västerbottens Intervention Project, a population-based cohort from northern Sweden. The GLACIER cohort is comparable to the full VIP cohort in demographic, anthropometric, and lifestyle characteristics.
GoDARTS	The Genetics of Diabetes Audit and Research in Tayside Scotland	9329309	European	Scotland	CAD/MI	First-ever CAD event. Defined as fatal and non-fatal myocardial infarction, unstable angina or coronary revascularisation	Controls were free of coronary artery disease, stroke and peripheral vascular disease.
HPS	MRC/BHF Heart Protection Study	15016485	European	UK	CAD/MI	History of MI, unstable or stable angina, coronary artery bypass grafting, or angioplasty	Population controls were used from the UK Twins Study and WTCCC2 National Blood Service collections
ITH	The INTERHEART Study	20031563	European	Worldwide	CAD/MI	Incident acute MI, presenting to a hospital within 24 hours of symptom	Age and sex matched hospital and community based, with no

Nature Genetics: doi:10.1038/ng.2480

						onset	previous diagnosis of heart disease or history of exertional chest pain
LOLIPOP	London Life Sciences Population study	18193046, 18454146, 19820698	South Asian	UK West London	CAD/MI	CAD was defined as a history of MI or coronary artery revascularization (CABG or PCI), or angiographically confirmed coronary artery stenosis greater than 50%. Clinical diagnosis of MI is based on two out of three of: 1. Chest pain, 2. Raised cardiac enzymes, 3. ECG changes.	Indian Asian men and women from the same cohort (aged 35 - 75 yrs), without diagnosis or history of CAD.
LURIC-EMIL	LUdwigshafen RIsk and Cardiovascular health study and Echinococcus Multilocularis and Internal Diseases in Leutkirch study	20065167 16981990	European	Germany	CAD/MI	Angiographically confirmed CAD (at least one coronary vessel with a stenosis > 50%) were included	GerBS control series that consists of healthy, unrelated blood donors recruited between May-July 2004 from the southwestern area of Germany / EMIL controls include population-based non-cases subjects
METSIM	METabolic Syndrome In Men	19223598	European	Kuopio, Finland	CAD/MI	CAD cases: Angiography-confirmed CAD, myocardial infarction, balloon angioplasty or coronary bypass	Controls were selected from the same population-based sample and were free from MI, coronary angiography, balloon angioplasty, cerebral infarction, cerebral hemorrhage, or any leg operation
MORGAM-FIN<br><br>MORGAM-FRA<br><br>MORGAM-GER<br><br>MORGAM-ITA<br>MORGAM-	MONICA, Risk, Genetics, Archiving, and Monograph	15561751 ref1	European	Finland: Southern Finland, North Karelia, Kuopio Province, Oulu Province, Turku/Loimaa, Helsinki. France: Lille, Strasbourg, Toulouse. Germany: Augsburg. Italy: Brianza. United Kingdom:	CAD/MI	All CAD: Incident definite or possible MI or coronary death, or unstable angina during follow-up, Coronary revascularization during follow-up, Documented MI at baseline, or an unclassifiable coronary death during follow-up. MI:Definite myocardial infarction.	Controls are 1:1 matched for cases (by age, sex, and region). They are participants who remained free of any cardiovascular disease at the age when the matched case had the first event.

Nature Genetics: doi:10.1038/ng.2480

UNK				Belfast.			
OHGS	The Ottawa Heart Genomics Study	17478681	European	Canada	CAD/MI	Cases had at least one of myocardial infarction, coronary artery bypass graft, percutenaeous intervention or a stenosis of at least 50% in at least one epicardial vessel. Diabetic cases and cases aged greater than 55 for men or 65 for women were excluded.	Controls were either asymptomatic for cardiovasular disease or had had a CTA or angiogram demonstrating no stenosis of greater than 50%. Controls were required to be at least 65 years old for men and 70 years old for women at the time of recruitment.
PIVUS	Prospective Investigation of the Vasculature in Uppsala Seniors	18489581	European	Sweden	CAD	Individuals within this cohort study who developed a fatal or non-fatal myocardial infarction or unstable angina during follow-up. Participants were identified as having CHD if they had a hospital admission with CHD as the primary cause of hospitalization and/or died with CAD as the underlying cause. CHD was defined as acute myocardial infarction (ICD-8 and ICD-9 code 410, ICD-10 codes I21-I22) or unstable angina (ICD-8 code 411, ICD-9 code 411B, ICD-10 code I20.0). The positive predictive values (i.e. validity) of the CHD diagnosis in the Swedish hospital discharge register has been demonstrated to be at least 95% when only primary diagnoses are considered.	Non-cases from the same longitudinal, community-based cohort study
PMB	Pfizer-MGH-Broad		European	Finland, Sweden	CAD/MI		
PopGen	PopGen	18362232	European	Germany	CAD	Unrelated German CAD patients recruited in Schleswig-Holstein, through the population-based PopGen biobank with significant CAD (at least a 70% stenosis in one major coronary vessel); age of onset < 55 y	population based controls
PROCARDIS	European collaborative study of the genetics of precocious coronary artery disease	18048406	European	Germany, Italy, Sweden, UK	CAD/MI	Symptomatic CAD before age 66 years and 80% of cases also had a sibling in whom CAD had been diagnosed before age 66 years. CAD was defined as clinically documented evidence of myocardial infarction (MI) (80%),	PROCARDIS controls had no personal or sibling history of CAD before age 66 years. PoBI and UK Twin study are population-based controls that were not screened for CAD.

Nature Genetics: doi:10.1038/ng.2480

						coronary artery bypass graft (CABG) (10%), acute coronary syndrome (ACS) (6%), coronary angioplasty (CA) (1%) or stable angina (hospitalization for angina or documented obstructive coronary disease) (3%). The cases included 2,136 cases who were half or full siblings.	
PROMIS*	The Pakistan Risk Of Myocardial Infarction Study	19404752	South Asian	Pakistan	CAD/MI	Acute myocardial infarction (MI) with typical ECG characteristics, a positive troponin test, and MI symptoms within the previous 24 hours	Controls have been recruited in the following order of priority: (i) visitors of patients attending the out-patient department; (ii) patients attending the out-patient department for routine non-cardiac complaints, or (iii) non-blood related visitors of index MI cases.
SCARF-SHEEP			European	Sweden	CAD/MI	First confirmed myocardial infarction	No history, symptoms or signs of cardiovascular disease
STR	Swedish Twin Registry	8981957	European	Sweden	CAD	Individuals within this cohort study who developed a fatal or non-fatal myocardial infarction or unstable angina during follow-up. Participants were identified as having CHD if they had a hospital admission with CHD as the primary cause of hospitalization and/or died with CAD as the underlying cause. CHD was defined as acute myocardial infarction (ICD-8 and ICD-9 code 410, ICD-10 codes I21-I22) or unstable angina (ICD-8 code 411, ICD-9 code 411B, ICD-10 code I20.0). The positive predictive values (i.e. validity) of the CHD diagnosis in the Swedish hospital discharge register has been demonstrated to be at least 95% when only primary diagnoses are considered.	Non-cases from the same longitudinal, community-based cohort study
THISEAS	The Hellenic Study of Interactions between Snps and Eating in Atherosclerosis Susceptibility	20167083	European	Greece	CAD/MI	First-ever mi before age of 70 yrs; first CAD (>50% stenosis in one of the three main coronary vessels assessed by coronary artery angiography - no history of ACS)	<30% stenosis assessed by coronary artery angiography or negative stress test; age matched without MI/CAD history

Nature Genetics: doi:10.1038/ng.2480

	(THISEAS) study						
ULSAM	Uppsala Longitudinal Study of Adult Men	12637978	European	Sweden	CAD/MI	Individuals within this cohort study who developed a fatal or non-fatal myocardial infarction or unstable angina during follow-up. Participants were identified as having CHD if they had a hospital admission with CHD as the primary cause of hospitalization and/or died with CAD as the underlying cause. CHD was defined as acute myocardial infarction (ICD-8 and ICD-9 code 410, ICD-10 codes I21-I22) or unstable angina (ICD-8 code 411, ICD-9 code 411B, ICD-10 code I20.0). The positive predictive values (i.e. validity) of the CHD diagnosis in the Swedish hospital discharge register has been demonstrated to be at least 95% when only primary diagnoses are considered.	Non-cases from the same longitudinal, community-based cohort study
WTCCC CAD2	The Wellcome Trust Case Control Consortium CAD2		European	UK	CAD/MI		1958 Birth Cohort

* submitted datasets with independent samples either directly genotyped on the Metabochip or results from GWAS
ref1 Tunstall-Pedoe H, editor. Prepared by Tunstall-Pedoe H, Kuulasmaa K, Tolonen H, Davidson M, Mendis S with 64 other contributors for The WHO MONICA Project. MONICA Monograph and Multimedia Sourcebook. Geneva: World Health Organization; 2003. ISBN 92 4 156223 4. Also available from http://www.ktl.fi/monica/public/monograph.html.

Nature Genetics: doi:10.1038/ng.2480

Supplementary Table 2b: General Study Characteristics for Stage 3 studies

Study	Full Study name	Reference	Ethnicity	Region of recruitment	Phenotype	Phenotype definition	Control definition
COROGENE	Corogene	21642350	European	Helsinki, Finland	Acute coronary syndrome patients	Acute coronary syndrome cases with coronary artery obstruction >50% at least in one coronary artery.	Area matched, healthy FINRISK population controls
FINCAVAS	The Finnish Cardiovascular Study	16515696	European	Finland	CAD/MI	Cases defined as >50% stenosis in one or more coronary arteries in coronary angiography, or strong Bayesian posterior probability for CAD after exercise test using a bicycle ergometer, or hospital verified myocardial infarction in history. Patients were recruited during 2001-2007, and the follow-up data was gathered at 2, 5 and 10 years.	Controls defined as <50% stenosis in coronary arteries, or low Bayesian posterior probability for CAD after exercise test, and no myocardial infarction in medical history.
GenRIC	Genomics Research in Cardiovascular disease	NA	East Asian	Seoul, Korea	CAD/MI	Angiographic definition: Significant reduction in luminal diameter due to coronary atheromatous disease (i.e. with stenosis greater than 50%)<br><br>Clinical definitions:<br>Stable angina: chest or arm discomfort that may not be described as pain but is reproducibly associated with physical exertion or stress and is relieved within 5-10 minutes by rest and/or sublingual nitroglycerin.<br>Unstable angina: Angina pectoris or equivalent ischemic discomfort with at least one of three features 1) it occurs at rest, usually resting > 10 minutes, 2) it is severe and of new onset (≤4-6 weeks), 3) it occurs with a crescendo pattern<br>Myocardial infarction: Spontaneous or secondary myocardial infarction according to the "universal definition of myocardial infarction"	Participants without CAD from a population based cohort

Nature Genetics: doi:10.1038/ng.2480

Supplementary Table 3a: Genotyping, Quality control and Statistical analysis for Stage 2 studies

Study	Genotyping Array	Genotyping Centre	Calling algorithm	Ethnicity	PCA/Additional QC	Imputation software/ Reference panel	Total SNPs submitted in ALL analysis [genotyped/imputed]	SNPs passing QC[1]	Analysis software	λ_{QT}
ADVANCE	Illumina CM[2]	HudsonAlpha	GenCall	European	Ethnic outliers, gender mismatch,	NA	193881/0	134,901	Plink	1.00
AMC-PAS	Illumina CM[2]	Sanger	GenoSNP	European	Heterozygosity, ethnic outliers, duplicates	NA	117088/0	117,088	Plink	1.08
Angio-Lueb/ KORA F3	Illumina CM[2]	Helmholtz Zentrum München	GenCall	European	Gender check, MDS adjustment, IBS/IBD check	NA	130610/0	130,609	Plink	1.08
Cardiogenics	Illumina CM[2]	Sanger	GenoSNP	European	Heterozygosity, ethnic outliers, duplicates<br>Center site study specific adjustment	NA	122839/0	122,839	Plink	1.07
DILGOM	Illumina CM[2]	Helsinki	Illuminus	European	NA	NA	181174/0	121,295	Plink	1.00
DUKE	Affymetrix Axiom	Canadian Cardiovascular Genetics Centre	BRLMM-P	European	Ethnic outliers, duplicates<br>SNP call rate ≥ 95%, MAF ≥ 5%, HWE $P > 10^{-6}$	IMPUTE v2.1.0<br>1kG first generation CEU + TSI	22455/74180	84,212	R	1.01
EGCUT GWAS	Illumina Human370CNV & OmniExpress	Estonian Genome Center of University of Tartu	Beadstudio	European	Ethnic outliers, gender check, cryptic relatedness<br>PCA<br>SNP call rate ≥ 95%, HWE $P > 10^{-6}$	IMPUTE v1.0<br>CEU build36 rel22	178087/2239235	85,852	SNPTEST	1.10
EGCUT Metabochip	Illumina CM[2]	Estonian Genome Center of University of Tartu	Beadstudio	European	Ethnic outliers, gender check, cryptic relatedness	NA	181174/0	128,757	Plink	1.06
EPIC	Illumina CM[2]	Sanger	GenoSNP	European	Heterozygosity, ethnic outliers	NA	122022/0	122,022	Plink	1.11
FGENTCARD	Illumina Human610-Quad, Human 660W-Quad	CNG, IntegraGen	GenCall	European	Phenotyping, ethnicity, heterozygosity, Beadchip PC<br>SNP call rate ≥ 98%, HWE $P > 10^{-7}$	Impute2<br>Hapmap2 rel22 CEU	513079/2100829	94,198	R/SNPTEST2	1.02
FRISCII GLACIER	Illumina CM[2]	Uppsala SNP&SEQ Technology Platform / Sanger	GenTrain 2.0 Illuminus	European	Heterozygosity, ethnic outliers, duplicates, missingness, HWE, gender mismatch, PCA<br>FRISCII<br>SNP call rate ≥ 98%, HWE $P > 10^{-6}$<br>GLACIER<br>SNP call rate ≥ 95%	NA	178783/0<br>150451/0	128,200	GenABEL	1.24
GoDARTS	Illumina CM[2]	Sanger	GenoSNP	European	Heterozygosity, ethnic outliers, duplicates, SNP call rate ≥ 98%,, MAF>0.01	NA	107402/0	118,658	SNPTEST	1.03
HPS	Illumina 610-Quad (cases and UK Twins controls)<br>Illumina 1M Duo (WTCCC2 NBS controls,	CNG, Evry, France	Beadstudio and Illuminus	European	Non-European ancestry, duplicates<br>SNP call rate ≥97.5%, HWE $P > 10^{-6}$	MACH<br>HapMap2 rel22	95610	92,881	Plink	1.05

Nature Genetics: doi:10.1038/ng.2480

	subset of 610k SNPs used for imputation)									
ITH	Affymetrix 6.0	Canadian Cardiovascular Genetics Centre	Birdseed - v2	European	Ethnic outliers, duplicates<br>SNP call rate ≥ 95%, HWE $P > 10^{-6}$	IMPUTE v2.1.0<br>1kG first generation CEU + TSI	27073/69976	86,750	R	1.00
LOLIPOP	Illumina Human610	DeCode	Beadstudio	South Asian	Ethnic outliers, duplicates, wrong gender, relatedness<br>Principal components, cohort<br>SNP call rate ≥95%, HWE $P > 10^{-6}$<br>MAF ≥ 1%	MACH<br>HapMap2 rel 21 combined data	544390/1710717	82,193	Plink/ MACH2qtl	1.05
LURIC-EMIL	Illumina CM^2			European	Heterozygosity, ethnic outliers<br>Principal components	NA	128596/0	128,596	Plink	1.13
METSIM	Illumina CM^2	CIDR	Beadstudio	European	Dropped SNP if Cluster Separation score < 0.2 (based on re-clustering using all samples) or which had more than 1<br>Replicate error as defined with the HapMap control samples.<br>Hand editing for X, Y and Mitochondrial loci.<br>Call rate >= .95, based on genotypes with quality score threshold 0.15.<br>Duplicate and gender check	NA	138992/0	129,131	Plink	1.02
MORGAM-FIN	Illumina CM^2	Sanger	GenCall	European	Heterozygosity, ethnic outliers, duplicates, gender check, relatedness<br>SNP call rate ≥ 99%	NA	128920/0	123,461	Plink	1.00
MORGAM-FRA	Illumina CM^2	Sanger	GenCall	European	Heterozygosity, ethnic outliers, duplicates, gender check, relatedness<br>SNP call rate ≥ 99%	NA	127793/0	120,156	Plink	1.04
MORGAM-GER	Illumina CM^2	Sanger	GenCall	European	Heterozygosity, ethnic outliers, duplicates, gender check, relatedness<br>SNP call rate ≥ 99%	NA	125969/0	120,099	Plink	0.90
MORGAM-ITA	Illumina CM^2	Sanger	GenCall	European	Heterozygosity, ethnic outliers, duplicates, gender check, relatedness<br>SNP call rate ≥ 99%	NA	129634/0	121,514	Plink	1.01
MORGAM-UNK	Illumina CM^2	Sanger	GenCall	European	Heterozygosity, ethnic outliers, duplicates, gender check, relatedness<br>SNP call rate ≥ 99%	NA	124600/0	120,268	Plink	1.04
OHGS	Affymetrix 500K and 6.0	Canadian Cardiovascular Genetics Centre	BRLMM and Birdseed-v2	European	Ethnic outliers, duplicates<br>SNP call rate ≥ 95%, HWE $P > 10^{-6}$	IMPUTE v2.1.0<br>1kG first generation CEU + TSI	26907/ 69887	86,151	R	1.16
PIVUS	Illumina CM^2	Uppsala SNP&SEQ Technology Platform	GenCall	European	SNP call rate ≥ 90%, HWE $P > 10^{-6}$	NA	133388/0	126,040	Plink	0.95
PMB	Illumina CM^2	Broad Institute	Birdseed	European	batch excluded based on heterozygosity, missingness	NA	179195/0	121,812	Plink	1.01

Nature Genetics: doi:10.1038/ng.2480

					outliers					
PopGEN	Illumina CM[2]	Helmholtz Zentrum München	GenCall	European	Gender check, IBS/IBD check	NA	125243/0	125,243	Plink	1.04
PROCARDIS	Illumina Infinium Human 1M, HumanHap 610 and Human 1.2M (WTCCC2 bespoke)	CNG, Evry, France and Sanger, UK	Beadstudio and Illuminus	European	Non-European ancestry outliers, duplicates<br>Country of origin robust(Huber-White/sandwich) standard error estimates to allow for relatedness<br>SNP call rate ≥ 97.5%, HWE P > 10-^{6}	MACH<br>HapMap2 r22	29957/65653	94,381	STATA	1.06
PROMIS GWAS	Illumina Quad 660	Sanger	GenoSNP	South Asian	Heterozygosity, ethnic outliers, duplicates, PCA<br>SNP call rate ≥97.5%, HWE P > 10^{-6}	Impute v2<br>Hapmap2 + Hapmap 3 GIH	529030/2670976	97,920	SNPTESTv2	1.05
PROMIS Metabochip	Illumina CM[2]	Sanger	GenoSNP	South Asian	Heterozygosity, ethnic outliers, duplicates	NA	127574/0	127,574	Plink	1.05
SCARF-SHEEP	Illumina CM[2]	Uppsala, Sweden	GenCall	European		NA	180475/0	125,894	Plink	1.07
STR	Illumina CM[2]	Uppsala SNP&SEQ Technology Platform	GenCall	European	SNP call rate >90%, HWE p>10^{-6}	NA	133430/0	127,479	Plink	1.03
THISEAS	Illumina CM[2]	Sanger	GenoSNP	European	Heterozygosity, ethnic outliers, gender mismatch	NA	121533/0	121,533	Plink	0.95
ULSAM	Illumina CM[2]	Uppsala SNP&SEQ Technology Platform	GenCall	European	SNP call rate >90%, HWE p>10^{-6}	NA	129336/0	123,677	Plink	1.10
WTCCC CAD2	Illumina CM[2]	Sanger	GenoSNP	European	Duplicates	NA	171730/0	120,383	Plink	0.89

MAF = Minor allele frequency, OR = Odds Ratio, HWE = Hardy Weinberg E…

[1]QC was applied to all studies centrally, Sample Call Rate > 0.98, MAF (in Cases and Controls) ≥ 0.001, HWE (Controls) ≥ 0.0001

[2] Illumina Cardio-Metabochip

Nature Genetics: doi:10.1038/ng.2480

Supplementary Table 3b: Genotyping, quality control and Statistical analysis for Stage 3 studies

Study	Genotyping Array	Genotyping Centre	Calling algorithm	Ethnicity	PCA/Additional QC	Imputation software/ Reference panel	Total SNPs submitted in ALL analysis [genotyped/imputed]	SNPs passing QC[1]	Analysis software	λ_{QT}
COROGENE	Illumina 610K	Sanger	Illuminus	European	heterozygosity, gender check and relatedness checks have been performed and any discrebansies have been removed. 8 individuals have been removed due to cryptic relatedness.	MACH, Hapmap 2	5/54 (replication snps)	59	Probabel	
FINCAVAS	Illumina CM[2]	Helmholtz Zentrum München	GenCall	European	Heterozygosity, relatedness, gender mismatch	NA	161183/0	123522	SNPTEST v2.2.0	0.99
GenRIC	Affymetrix Human SNP array 6.0	Korea National Institute of Health	BirdSeed	East Asian	Gender, Heterozygosity, cryptic first degree relatives	IMPUTE (Ver1.0)	599,226/ 1,631,947	52724	R package	1.12

MAF = Minor allele frequency, OR = Odds Ratio, HWE = Hardy Weinberg E…

[1]QC was applied to all studies centrally, Sample Call Rate > 0.98, MAF (in Cases and Controls) ≥ 0.001, HWE (Controls) ≥ 0.0001

Nature Genetics: doi:10.1038/ng.2480

Supplementary Table 4: Loci not reaching genome-wide significance in Stage 3

				Stage 1		Stage 2		Combined (Stage 1,2)	Stage 3		Combined (Stage 1,2,3)
**SNP**	**Chr**	**Nearest Gene(s)**	**Effect/Non Effect allele (frequency)**	**OR**	***P***	**OR**	***P***	***P***	**OR**	***P***	***P***
rs246600	5	ARHGAP26/KIAA0621	C/T (0.46)	1.06	1.16E-04	1.04	7.55E-05	1.71E-07	1.01	8.05E-01	2.36E-07
rs11057841	12	SCARB1	T/C (0.15)	1.11	7.22E-05	1.06	1.10E-04	1.56E-07	1.02	7.45E-01	1.78E-07
rs7219320	17	TOM1L2/LRRC48/ATPAF2	A/G (0.40)	1.07	1.37E-05	1.04	6.62E-04	1.77E-07	1.01	7.23E-01	4.95E-07
rs867186	20	PROCR	A/G (0.88)	1.08	1.56E-03	1.07	1.23E-05	3.59E-07	1.08	1.51E-01	5.12E-08

The combination of Stage 1, 2and 3 was performed using a sample size weighted meta-analysis.

Nature Genetics: doi:10.1038/ng.2480

Supplementary Table 5: Subgroup analyses results

			Males		Females		Young		Old		MI	
**Known Loci**	**SNP**	**Effect/ Non Effect allele**	**Stage 2 OR**	**Stage 1 and Stage 2 *P***	**Stage 2 OR**	**Stage 1 and Stage 2 *P***	**Stage 2 OR**	**Stage 1 and Stage 2 *P***	**Stage 2 OR**	**Stage 1 and Stage 2 *P***	**Stage 2 OR**	**Stage 1 and Stage 2 *P***
SORT1	rs602633	G/T	1.14	1.32E-18	1.06	3.42E-05	1.21	2.77E-20	1.10	4.97E-08	1.11	2.23E-16
PCSK9	rs11206510	T/C	1.05	1.55E-02[1]	1.02	4.07E-01[1]	1.07	2.04E-02[1]	1.04	7.12E-04[1]	1.05	7.03E-05
WDR12	rs6725887	C/T	1.09	1.88E-07	1.11	6.55E-08	1.04	1.70E-04	1.10	1.48E-07	1.09	5.51E-10
MRAS	rs9818870	T/C	1.07	2.97E-07	1.01	1.84E-02	1.03	1.06E-05	1.05	3.26E-07	1.06	6.44E-11
TCF21	rs12190287	C/G	1.06	3.51E-09	1.04	7.54E-03	1.04	7.78E-04	1.05	5.34E-09	1.05	3.05E-10
SLC22A3/LPAL2/LPA	rs3798220	C/T	1.25	3.62E-03[1]	1.39	2.14E-03[1]	n/a	n/a	1.22	2.13E-03[1]	1.26	2.09E-03[1]
	rs2048327	C/T	1.05	9.55E-07	1.05	3.75E-05	1.06	1.51E-07	1.05	8.31E-08	1.04	2.59E-06
ZC3HC1	rs11556924	C/T	1.08	4.83E-12	1.08	4.70E-05	1.08	5.73E-10	1.06	4.68E-07	1.07	3.18E-12
CDKN2BAS	rs1333049	C/G	1.23	3.93E-88	1.17	4.42E-24	1.23	3.01E-61	1.20	4.03E-41	1.21	2.57E-80
	rs3217992	T/ C	1.16	4.66E-48	1.09	1.57E-08	1.15	5.43E-31	1.14	6.01E-36	1.14	5.69E-41
ABO	rs579459	C/T	1.04	2.82E-08	1.04	2.20E-03	1.04	1.22E-04	1.03	2.15E-04	1.03	2.17E-07
CYP17A1/CNNM2/NT5C2	rs12413409	G/A	1.07	1.11E-05	1.11	2.20E-06	1.06	1.67E-06	1.08	1.36E-07	1.09	5.33E-08
KIAA1462	rs2505083	C/T	1.06	4.16E-08	1.04	3.36E-03	1.07	5.74E-06	1.05	1.91E-06	1.05	2.14E-04
PDGFD	rs974819	T/C	1.09	5.41E-10	1.04	1.43E-02	1.13	3.44E-09	1.07	1.89E-08	1.07	6.99E-09
SH2B3	rs3184504	T/C	1.06	3.23E-06	1.08	8.11E-05	n/a	n/a	1.07	1.58E-10	n/a	n/a
COL4A1/COL4A2	rs4773144	G/A	1.07	2.76E-09	1.03	7.37E-03	1.05	2.54E-05	1.06	1.32E-07	1.05	7.08E-07
	rs9515203	T/C	1.08	5.39E-09	1.04	6.98E-03	1.10	3.08E-09	1.06	3.90E-04	1.05	1.40E-06
HHIPL1	rs2895811	C/T	1.04	1.35E-03[1]	1.03	1.03E-01[1]	1.05	2.10E-02[1]	1.04	3.04E-03[1]	1.04	7.47E-03[1]
RAI1/PEMT/RASD1	rs12936587	G/A	1.04	5.97E-08	1.04	1.06E-02	1.05	1.34E-05	1.03	2.27E-06	1.04	5.50E-08
LDLR	rs1122608	G/T	1.07	4.52E-10	1.05	2.87E-02	1.07	2.70E-09	1.06	7.00E-07	1.06	5.39E-08
gene_desert/KCNE2	rs9982601	T/C	1.09	3.81E-09	1.09	1.72E-03	1.13	1.90E-12	1.09	7.18E-08	1.07	1.74E-10
PPAP2B	rs17114036	A/G	1.08	8.07E-08	1.10	2.66E-04	1.08	8.23E-04	1.10	1.43E-09	1.09	2.16E-08
ANKS1A	rs12205331	C/ T	1.02	1.43E-04	1.01	4.95E-02	0.99	1.69E-03	1.02	5.73E-03	1.02	6.44E-04
PHACTR1	rs9369640	A/ C	1.10	2.96E-18	1.05	2.29E-06	1.10	1.17E-08	1.09	1.49E-10	1.10	8.55E-20
CXCL12	rs501120	T/ C	1.06	1.25E-05	1.06	1.39E-04	1.09	6.49E-08	1.04	3.18E-07	1.08	8.56E-11
	rs2047009	G/ T	1.06	9.41E-11	1.02	9.89E-02	1.07	3.50E-08	1.04	3.43E-06	1.04	8.24E-07
LIPA	rs2246833	T/ C	1.06	2.39E-06	1.02	2.25E-01	1.08	6.86E-07	1.03	2.46E-04	1.05	2.29E-08
	rs11203042	T/ C	1.03	4.23E-04	1.02	5.19E-02	1.04	3.86E-06	1.02	8.25E-05	1.02	3.09E-07
UBE2Z	rs15563	G/A	1.01	5.82E-05	1.02	6.39E-03	1.01	3.86E-04	1.01	2.93E-03	1.01	1.68E-03
SMG6	rs2281727	G/A	1.04	1.33E-05	1.01	4.98E-03	1.05	2.94E-10	1.03	6.46E-05	1.04	2.63E-07
MIA3	rs17464857	T/ G	1.03	9.80E-03	1.01	5.88E-02	1.02	2.61E-02	1.04	1.14E-03	1.03	8.77E-04
ZNF259/APOA5/APOA1	rs9326246	C/G	1.03	1.92E-04	1.09	1.03E-03	1.02	1.16E-01	1.05	2.27E-04	1.04	1.69E-04
ADAMTS7	rs7173743	T/C	1.05	2.78E-08	1.06	2.23E-05	1.06	1.96E-06	1.06	1.92E-09	1.06	1.57E-10
ApoE/ApoC1	rs2075650	G/A	1.11	6.72E-08[1]	1.12	1.08E-04[1]	1.17	4.56E-08[1]	1.09	7.10E-07[1]	1.12	6.54E-09[1]
	rs445925	G/A	1.14	2.01E-07[1]	1.10	9.52E-03[1]	1.15	1.99E-04[1]	1.11	3.26E-03[1]	1.13	2.50E-07[1]
**Novel loci**												
IL6R	rs4845625	T/C	1.03	6.55E-05	1.07	3.74E-04	1.05	2.01E-04	1.04	3.67E-05	1.05	4.30E-07
APOB	rs515135	C/T	1.08	2.93E-09	1.07	2.47E-02	1.07	2.57E-04	1.08	5.42E-08	1.08	1.96E-09
ZEB2-AC074093.1	rs2252641	C/T	1.04	4.19E-06	1.05	9.80E-04	1.04	9.88E-04	1.04	6.44E-05	1.03	5.03E-04
GGCX/VAMP8	rs1561198	T/C	1.06	1.66E-07	1.02	1.00E-01	1.08	1.43E-09	1.04	1.60E-03	1.06	1.77E-07
GUCY1A3	rs7692387	G/A	1.05	1.12E-06	1.06	2.55E-03	1.03	1.34E-02	1.06	4.06E-06	1.04	8.87E-05

Nature Genetics: doi:10.1038/ng.2480

SLC22A4/SLC22A5	rs273909	G/A	1.09	9.14E-07	1.09	9.16E-03	1.08	1.34E-03	1.08	5.58E-05	1.07	9.68E-04
KCNK5	rs10947789	T/C	1.07	6.86E-06	1.02	9.64E-02	1.08	2.05E-04	1.05	1.14E-04	1.07	3.65E-08
PLG	rs4252120	T/C	1.04	7.93E-06	1.10	1.48E-05	1.07	7.44E-05	1.06	3.54E-05	1.06	1.56E-05
LPL	rs264[2]	G/A	1.06	5.39E-07	1.05	1.88E-03	1.06	6.04E-06	1.07	3.45E-07	1.04	7.38E-08
	rs894210[2]	G/A	1.04	1.41E-06	1.02	6.45E-02	1.03	6.17E-04	1.04	1.47E-07	1.04	2.99E-06
	rs1569209[2]	T/G	1.12	8.30E-07	1.03	5.65E-02	1.11	1.67E-07	1.13	2.41E-07	1.08	1.19E-06
FLT1	rs9319428	A/G	1.05	1.79E-07	1.06	5.41E-03	1.06	4.49E-04	1.05	6.74E-07	1.05	4.16E-05
FURIN/FES	rs17514846[3]	A/C	1.05	2.06E-07	1.06	6.47E-04	1.03	3.21E-03	1.06	1.27E-08	1.07	1.52E-09
	rs4932370[3]	A/G	1.06	1.03E-07	1.07	5.51E-04	1.05	1.60E-02	1.07	8.44E-08	1.06	2.81E-05
TRIB1	rs2954029	A/T	1.05	4.22E-06	1.02	2.45E-04	1.06	5.49E-07	1.05	6.26E-05	1.04	1.03E-05
ABCG5/ABCG8	rs6544713	T/C	1.07	1.21E-10	1.06	4.60E-03	1.07	1.55E-04	1.05	1.53E-06	1.05	4.43E-05
EDNRA	rs1878406	T/C	1.06	6.23E-08	1.06	1.37E-03	1.07	1.43E-04	1.07	5.32E-06	1.05	3.96E-03
HDAC9	rs2023938	C/T	1.07	2.30E-05	1.11	1.39E-03	1.07	3.02E-03	1.07	1.71E-04	1.09	1.24E-03
AK097927	rs16986953	A/G	1.11	2.00E-08	1.03	5.06E-01	1.17	1.68E-08	1.11	5.52E-04	1.10	4.06E-06

Males: Logistic regression of all male cases versus all male controls, adjusted for age; Females: Logistic regression of all female cases versus all female controls, adjusted for age; Young: Logistic regression of all cases with early age of onset (<=50 years) versus all controls, adjusted for sex; Old: Logistic regression of all cases with late age of onset (>50 years) versus all controls, adjusted for sex; MI: Logistic regression of all MI cases versus all controls, adjusted for age and sex

[1]Stage 2 p-value

[2]Pair-wise R^2: rs264-rs894210 0.13; rs264-rs1569209 0.43; rs894210-rs1569209 0.12

[3] Pair-wise R^2: rs17514846-rs4932370 0.41

Nature Genetics: doi:10.1038/ng.2480

Supplementary Table 6: Expression Analyses

Locus	SNP ID	CAD SNP	r^2 with lead SNP (CEU)	Transcript	Tissue	*p*-value	*cis*-eSNP	r^2 with lead SNP (CEU)	*p*-value	*Conditional p*-value
**cis- Expression QTL Analysis**							**Strongest eQTLassociation**			
VAMP5-VAMP8-GGCX	rs1561198	Lead		*VAMP8*	LCL	1.13E-19	rs3770098	0.87	3.50E-23	0.95
	rs1561198	Lead		*VAMP8*	Skin	1.38E-13	rs6757263	0.90	2.48E-17	0.0778
	rs1561198	Lead		*GGCX*	mammary artery		rs12714147	0.18	3.92E-05	0.273
	rs1561198	Lead		*GGCX*	Liver	0.00053	rs11680227	0.30	0.000637	0.058
	rs1561198	Lead		*GGCX*	subqutaneous fat	9.88E-16	rs6705971	0.94	5.94E-23	0.473
	rs1561198	Lead		*GGCX*	omentum	1.97E-07	rs6738645	0.94	2.45E-12	0.172
PLG	rs4252120	Lead		*PLG*	LCL	1.29E-14	rs4252165	0.96	9.85E-16	0.09
FURIN- FES	rs17514846	Lead		*FES*	LCL	5.74E-23	rs6227	0.46	4.50E-33	0.013
	rs4932178	proxy	0.611*	*FES*	Fibroblasts	3.4E-04	rs4932178	1	n/a	n/a
	rs17514846	Lead		*FES*	omentum	1.9E-04	rs17514846	1	n/a	n/a
	rs17514846	Lead		*FURIN*	omentum	4.81E-13	rs4702	0.57	3.92E-30	1.1E-04
	rs17514846	Lead		*FES*	subqutaneous fat	1.10E-08	rs17514846	1	n/a	n/a
	rs17514846	Lead		*FURIN*	subqutaneous fat	7.44E-08	rs6227	0.46	9.41E-21	0.0034
**Allelic Imbalance Expression Analysis**										
VAMP5-VAMP8-GGCX	rs1561198	Lead		*GGCX*	LCL (HapMap CEU)	1.35E-08	rs6739015	0.87	4.64E-11	
	rs1561198	Lead		*GGCX*	Fibroblasts	8.37E-10	rs7591175	0.87	1.29E-10	
	rs1561198	Lead		*GGCX*	monocytes	1.43E-25	rs699664	0.667	1.57E-31	2.48E-05
LPL	rs264	Lead		*LPL*	monocytes	1.72E-13	rs269	0.725	1.23E-15	
FURIN- FES	rs4932179	proxy	0.656	*FES*	monocytes	7.00E-14	rs4932179	1	n/a	
	rs2071410	proxy	0.86*	*FES*	LCL (HapMap CEU)	1.23E-05	rs1573643	0.8	1.08E-05	

* r^2 to rs2521501 which is independent signal to rs17514846

Nature Genetics: doi:10.1038/ng.2480

Supplementary Table 7a: Mouse Model Details for Novel Loci

SNP	Chr	Nearest Gene(s)	Homozygous null Phenotype	Affected Anatomical Systems	If Cardiovascular or Metabolic genotype: Strain/Stock Designation	Reference (PubMed)	Affected systems
rs4845625	1	IL6R	Defective T helper 17 cell development. Abnormal inflammatory response and abnormal wound healing.	Ho, Im, A, L, En	NA		
rs6544713	2	ABCG5	Hyperabsorption of dietary plant sterols. Sitosterolemia, anemia, leukopenia, macrothrombocytopenia, other hematologic defects, cardiomyopathy, high plasma phytosterol levels and premature death.	C, Ho, L, Mo, Rp, He, B, G, Im, Mu	$Abcg5^{trac}$<br>$Abcg5/Abcg8^{tm1Hobb}$	19846887<br>12444248	Cardiac fibrosis, Cardiomyopathy<br>Abnormal intestinal lipid absorption<br>abnormal circulating lipid level, decreased cholesterol<br>abnormal cholesterol homeostasis
		ABCG8	Fail to secrete cholesterol into bile and exhibit increased plasma and tissue plant sterol levels	D, Ho, L, He	$Abcg8^{tm1Elk}$	15040800	Increased circulating plant sterol and triglyceride, decreased circulating cholesterol
rs515135	2	APOB	Usually die by midgestation; longer survivors exhibit exencephaly. Heterozygotes show reduced plasma cholesterol and apolipoprotein levels.	C, Em, G, Ho, L, Mo, N	$Apob^{tm4Sgy}$<br>*$Apob^{tm2Sgy}$*<br>$Apob^{tm1.1Zc}$<br>$Apob^{tm1Mae}$	9502790<br>8692825<br>10893242	Hemorrhage<br>Atherosclerotic lesions<br>decreased circulating triglyceride, HDL, LDL, VLDL
rs2252641	2	ZEB2	No Mouse model<br>Variety of defects at embryonic day 8.5 and die between E9.5 and 10.5.	NA<br>Mo, Ey, Rp, G, N, Em	NA<br>NA		
		ACVR2A	Most appear normal, a few display skeletal and facial abnormalities. As adults, follicle-stimulating hormone is suppressed, affecting reproduction.	C, Mo, G, Ey, Rp, N, Em, En, S	$Acvr2a^{tm1Hsch}$	10452853	Transposition of great arteries<br>Abnormal heart development
rs1561198	2	GGCX	50% of embryos die between E9.5 and E18, those surviving to term die of massive intra-abdominal hemorrhage shortly after birth with no evidence of ectopic calcification	C, Ho, Mo, S	$Ggcx^{tm1Dgi}$	17327402	Internal hemorrhage
		VAMP8	Postnatal lethality, hydronephrosis, and reduced amylase secretion, type I hypesensitivity reaction, and platelet activation	Ho, Rn, B, Mo, En, Im, D, G, He	NA		
rs7692387	4	GUCY1A3	Mild elevation of systolic blood pressure, abnormal blood vessel and platelet responses to NO	C, Rs, Mo, He, Ho, Mu	$Gucy1a3^{tm1.1Brou}$<br>$Gucy1a3^{tm1.1Dko}$	16886062<br>16614755	Abnormal right ventricle pressure

Nature Genetics: doi:10.1038/ng.2480

							Increased systemic arterial blood pressure<br>Abnormal vasodilation<br>Abnormal platelet aggregation
rs1878406	4	EDNRA	Perinatal death with cardiac and craniofacial malformations	C, Ho, Mo, N, R, Mu, En, Em, D, I, Rs, S, He	$Ednra^{tm2.1Hku}$<br>$Ednra^{tm5Hku}$<br>$Ednra^{tm1Ywa}$	18199583<br>20929948<br>9449664	Abnormal heart morphology
rs273909	5	SLC22A4<br>SLC22A5	No Mouse model<br>Systemic carnitine deficiency, cardiac hypertrophy, impaired Na-dependent carnitine transport, fatty liver, hypoglycemia, high postnatal mortality, and male infertility	NA<br>C, Mo, G, Ho, L, Rn, Rp	NA<br>$Slc22a5^{jvs}$	 Mouse Genome 86,Hayakawa *et al*	 Cardiac hypertrophy<br>abnormal glucse homeostasis/ hypoglycemia
rs10947789	6	KCNK5	Smaller than normal and prenatal lethality depending on genetic background.	Rs, N, Mo, G, Rn	NA		
rs4252120	6	PLG	Retarded growth, variable rectal prolapse, impaired fertility and lactation in females, early mortality, and widespread fibrin deposition and thrombotic lesions in liver, lung, stomach and other tissues.	C, D, En, G, Ho, Im, Int, L, Mo, N, Rn, Rp, Rs, T, Ey	Plg^{tm1Jld}	7705657	Decreased angiogenesis<br>Venoocclusion<br>Atherosclerotic lesions<br>Abnormal heart morphology<br>Abnormal blood coagulation and wound healing<br>Decreased circulating HDL
rs2023938	7	HDAC9	Mice with disruptions in this gene display age dependent cardiac hypertrophy	C, Mo, G, Mu	$Hdac9^{tm1Eno}$	12202037	Cardiac hypertrophy<br>Ventricular septal defect<br>hemorrhage<br>abnormal myocardium layer morphology
rs264	8	LPL	Cyanotic and die within 2 days of birth due to chylomicron engorgement of capillaries. Mutants show hypertriglyceridemia and reduced fat stores..	C, Ho, Mu, Mo	Lpl^{tm1Ijg}<br>Lpl^{tm1Sem}<br>$Lpl^{tm1Bres}$	15028738<br>7759497<br>8675619	Cardiac fibrosis<br>Abnormal cardiovascular system physiology<br>Decreased cardiac muscle contractility<br>Increased left ventricle diastolic and systolic pressure<br>Decreased circulating HDL, LDL<br>Increased circulating VLDL and triglyceride level<br>Abnormal glucose homeostasis<br>Thrombosis
rs2954029	8	TRIB1	Macrophages exhibit impaired IL12 response to LPS, MALP-1, or CpG DNA.	G, Im	NA		
rs9319428	13	FLT1	exhibit an excess of hemangioblasts resulting in an overgrowth of endothelial cells, abnormalities of vascular	C, Ey, Em, He, Mo, Ho, Mu, G	$Flt1^{tm1Jrt}$	7596436	Abnormal heart development and blood vessel morphology

Nature Genetics: doi:10.1038/ng.2480

			channels and blood islands,				Increased angiogenesis<br>Abnormal blood circulation<br>Increased response of heart to induced stress
rs17514846	15	FURIN	Die at E10.5-E11.5. multiple tissue abnormalities including abnormal yolk sac vasculature and chorioallantoic fusion, failure of axial rotation, a kinked neural tube, exencephaly and severe ventral closure and cardiac defects.	C, Mo, Em, G, N	$Furin^{tm1Ajmr}$	9811571	Absent vitelline blood vessels<br>Cardia bifida
		FES	NA	C, T, Mo, He, G, Im, Ey, Ho, N, D, Mu, Rp	Fes^{tm2Mcs}	11977979	Abnormal vasculogenesis and heart morphology<br>Hemorrhage

Data from International Knockout Mouse Consortium (IKMC) database accessed 12/9/2011, PMID 21677750 Skarnes *et al*., Nature 2011, Jun 15;474(7351):337-42
E = Embryonic day, NA = Not Available
Affected Anatomical systems key: Adipose^{A}, Behaviour^{B}, Cardiovascular^{C}, Cellular^{Ce}, Digestive/alimentary^{D}, Embryogenesis^{Em}, Endocrine/exocrine^{En}, Eye^{Ey}, Growth/size^{G}, Haemapoetic^{He}, Homeostasis/metabolic^{Ho}, Immune^{Im}, Integument^{Int}, Liver/biliary system^{L}, Mortality/aging^{Mo}, Muscle^{Mu}, Nervous system^{N}, Renal/urinary^{Rn}, Reproductive^{Rp}, Respiratory^{Rs}, Skeleton^{S}, Tumorigenesis^{T,}

Nature Genetics: doi:10.1038/ng.2480

Table 7b: Mouse Model Details for Known Loci

Lead SNP	Chr	Nearest Gene(s)	Homozygous null Phenotype	Affected Anatomical Systems	If Cardiovascular or Metabolic genotype: Strain/Stock Designation	Reference (PubMed)	Affected systems
rs17465637	1	MIA3	No mouse model	NA	NA		
rs11206510	1	PCSK9	Increased clearance of circulating cholesterol and decreased plasma cholesterol levels	Ho, L	$Pcsk9^{tm1.1Prat}$<br>$Pcsk9^{tm1.2Prat}$<br>$Pcsk9^{tm1Jdh}$	18666258<br>18666258<br>15805190	Decreased circulating HDL and LDL
rs17114036	1	PPAP2B	No survival past E10.5, defects in extraembryonic vasculogenesis and axis patterning	C, Mo, G, E, Ho, N	$Ppap2b^{tm1Stw}$<br>$Ppap2b^{tm2Stw}$<br>$Ppap2b^{tm3.1Stw}$	12925589<br>17610274	abnormal vasculogenesis<br>hemorrhage<br>abnormal phospholipid level
rs599839	1	PSRC1<br>SORT1	No phenotype<br>Increased protection from age- and injury-related neuron lose	NA<br>Ce, Ey, Ho, Im, N	NA<br>NA		
rs6725887	2	WDR12	No phenotype	NA			
rs98188708	3	MRAS	Homozygous for insertional mutation that inactivates gene are grossly normal, no morphological or neurological defects; mutant astrocytes	No phenotype			
rs17609940	6	ANKS1A	No mouse model	NA			
rs12526453	6	PHACTR1	No phenotype	NA			
rs2048327	6	SLC22A3<br>LPAL2<br>LPA	Normal phenotype<br>No mouse model<br>No mouse model	NA<br>NA<br>NA			
rs12190287	6	TCF21	Hypoplastic lungs and kidneys with abnormal vasculature of these organs and hemopericardium. Die at birth due to respiratory failure. Some mutations are also asplenic. Some alleles cause sex reversal in XY mice.	C, Mo, Ho, Rs, He, R, Im, In, D, M, En	$Tcf21^{tm1Jrt}$	10572052	abnormal lung and kidney vasculature morphology<br>hemopericardium
rs11556924	7	ZC3HC1	No phenotype	NA			
rs579459	9	ABO	No phenotype	NA			
rs1333049	9	CDKN2A	Null mutants of p16INK4a or p19ARF proteins each show increased tumor susceptibility and sensitivity to carcinogens. Loss of both gives very early onset. p19ARF nulls also show thymic hyperplasia and the eye's hyaloid vascular system fails to regress.	T, Int, Ey, Ho, Mo, Em, Rs, N, En, B	NA		

Nature Genetics: doi:10.1038/ng.2480

		CDKN2B	Increased tumor incidence, lymphoid hyperplasia, and extramedullary hematopoiesis	T, Int, Mo, He, Im, En, Rp, Ho	NA		
rs501120	10	CXCL12	Late embryonic lethality, impaired myelopoiesis, abnormal cerebellum development, abnormal germ cell migration, abnormal angiogenesis around the stomach, and ventricular septal defects.	C, N, L, Im, Rp, He, Mo	$Cxcl12^{tm1Tng}$<br>$Cxcl12^{tm3.1(HBEGF/EGFP)Tng}$	8757135<br>20850355	Abnormal angiogenesis<br>Perimembraneous ventricular septal defect<br>Liver hemorrhage
rs12413409	10	CYP17A1<br>CNNM2<br>NT5C2	Early embryonic lethality<br>No phenotype<br>No phenotype	Ho, B, N, Rp, Mo			
rs10953541	10	KIAA1462	No mouse model	NA			
rs1412444	10	LIPA	No phenotype	C, Mo, L, Im, Ho, A, D, En, R, G, He, B, Rs, N, Int	$Lipa^{tm1Ggb}$	9700186	abnormal liver sinusoid morphology<br>increased circulating insulin level, insulin resistance<br>decreased circulating HDL, increased LDL, free fatty acid
rs974819	11	PDGFD	No phenotype	NA			
rs964184	11	ZNF259	No mouse model	NA			
		APOA5	Increased triglyceride and VLDL cholesterol levels	Ho	$Apoa5^{tm1Hgc}$	11588264	increased circulating VLDL, triglyceride
		APOA1	Reduced HDL, non-HDL cholesterol, and cholesterol ester levels, increased plasma triglyceride and free cholesterol levels, impaired corticosteroid synthesis	C, Ho, He, Im, Ce, L, En	$Apoa1/Apoc3/Apoa4^{tm1Hmez}$<br>$Apoa1^{tm1Unc}$	16497661<br>1496008	increased susceptibility to atherosclerosis<br>abnormal blood vessel healing<br>decreased cholesterol efflux<br>decreased circulating HDL, LDL, VLDL,<br>increased circulating triglyceride
rs2258916	12	HNF1A	Die at 3-6 weeks from progressive wasting syndrome, liver and renal dysfunction and type II diabetes. Mutants have little or no phenylalanine hydroxylase, albumin, alpha 1-antitrypsin and secreted insulin	Mo, G, Ho, L	$Hnf1a^{tm1.1Ylee}$<br>$Hnf1a^{tm1Mya}$<br>$Hnf1a^{tm2Mya}$<br>$Hnf1a^{tm2Ylee}$	9566924<br>8598044<br>8598044<br>12529398	Increased cholesterol level<br>decreased circulating insulin/ hyperglycaemia
		C12orf43	No mouse model				
rs3184504	12	SH2B3	No phenotype	He, Im, Ce			
rs4773144	13	COL4A1	Various eye and vision defects. Newborn mutants may also exhibit bruises	C, V, Mo, G, Em, N, Ho, R	$Col4a1/Col4a2^{tm1Epo}$<br>$Col4a1^{Bru}$	14998921	dilated vasculature<br>hemopericardium
		COL4A2	Variable phenotype affecting the eye, brain and vascular stability	C, N, Ho, G, Em, V, He, Mo	$Col4a1^{deltaex40}$<br>$Col4a1^{ENU4004}$<br>$Col4a1^{ENU911}$	15905400<br>10886015<br>3724777	bruising<br>hemorrhage<br>corneal vascularization

Nature Genetics: doi:10.1038/ng.2480

					Col4a1^{Raw}<br>Col4a1^{Svc}<br>Col4a2^{ENU4003}<br>Col4a2^{ENU415}	11929848<br>11929848<br>10886015<br>6877261	
rs2895811	14	HHIPL1	No phenotype	NA			
rs3825807	15	ADAMTS7	No phenotype	NA			
rs12936587	17	RAI1	Usually die as embryos. Survivors have shortened life spans and show severe craniofacial and axial skeleton defects	Mo, S, N, A, Ho, G, B, Rs			
		PEMT	Normal phenotype on normal diets but display liver abnormalities on choline deficient diets or high fat and cholesterol diets.	N, L, Ho, G, En, B, Mo	Pemt^{tm1J}	9371769	decreased circulating HDL
		RASD1	Reduced ability to entrain to low intensity light with resulting abnormalities in circadian rhythm.	B			
rs216172	17	SMG6	No phenotype	NA			
rs46522	17	UBE2Z	No phenotype	NA			
rs1122608	19	LDLR	2X higher total plasma cholesterol and 7-9X higher IDL and LDL levels on a normal diet compared to controls. On a high cholesterol diet, mutant effects dramatically increase and mice develop xanthomatosis and atherosclerosis.	C, Ho, N, A, G, Im, L, B, He, En, In, Ey, Mo, Mu, Ce	Ldlr^{tm1Her}<br>Ldlr^{Hlb301}<br>Ldlr$^{tm1(LDLR)Mae}$	8349823<br><br>11076954	Multiple different abnormal morphologies<br>Arteriosclerosis<br>Aortic aneurysm<br>Artery occlusion<br>Altered response to myocardial infarction<br>increased and decreased circulating HDL, VLDL, triglyceride<br>decreased circulating LDL
rs2075650	19	APOE	Mutations at this locus cause diet-induced hypercholesterolemia and atherosclerosis. Mutants also develop foam-cell rich deposits in proximal aorta, impaired blood-nerve and blood-brain barriers, and many xanthomatous lesions.	C, Ho, N, Rn, Mu, L, A, G, En, D, Ce,B, Im, Rs, Rp, S, Ey, He, Mo, Int	Apoe$^{tm1(APOE)Kyan}$<br>Apoe$^{tm1(APOE)Sfu}$<br>Apoe$^{tm1(APOE*2)Mae}$<br>Apoe^{shl}<br>Apoe$^{Tg(rtTA)1Gaga}$<br>Apoe^{tm1Khw}<br>Apoe^{tm1Lmh}	11930145<br>10655544<br>9649566<br>10087291<br>18464897<br>11792702<br>7840811	Abnormal circulating cholesterol levels (increased and decreased LDL, HDL, VLDL and triglyceride levels)<br><br><br><br><br>atherosclerotic lesions
rs9982601	21	gene_desert<br>KCNE2	No phenotype<br>Stomach hyperplasia and achlorhydria	NA<br>D, Ho			

Data from International Knockout Mouse Consortium (IKMC) database accessed 25/10/2011, PMID 21677750 Skarnes *et al*., Nature 2011, Jun 15;474(7351):337-42
E = Embryonic day, NA = Not Available
Affected Anatomical systems key: Adipose^{A}, Behaviour^{B}, Cardiovascular^{C}, Cellular^{Ce}, Digestive/alimentary^{D}, Embryogenesis^{Em}, Endocrine/exocrine^{En}, Eye^{Ey}, Growth/size^{G}, Haemapoetic^{He}, Homeostasis/metabolic^{Ho}, Immune^{Im}, Integument^{Int}, Liver/biliary system^{L}, Mortality/aging^{Mo}, Muscle^{Mu}, Nervous system^{N}, Renal/urinary^{Rn}, Reproductive^{Rp}, Respiratory^{Rs}, Skeleton^{S}, Tumorigenesis^{T,}

Supplementary Table 8: Risk Factors

Locus	RSID	SNP	CAD P-value	Risk factor summary	Global Lipids Genetics Consortium P-value and Effect Direction								ICBP P-value		MAGIC P-value and Effect Direction										DIAGRAM P-value and Effect Direction		GIANT P-value	
				Trait	TC		LDL-C		HDL-C		TG		SBP	DBP	FG		FI		2hG		H-B		H-IR		T2D		BMI	WHR
				n	97743		93070		97527		94244		69395	69395	46186		38238		15234		36466		37037		NA		122483	76191
Additional Loci																												
IL6R	rs4845625	T/C	3.55 E-08	None	5.79 E-01	+	5.51 E-01	+	5.61 E-01	+	5.74 E-01	+	2.68 E-01	2.01 E-01	7.84 E-01	+	9.14 E-01	-	2.25 E-01	+	9.29 E-01	-	7.25 E-01	+	6.36 E-01	+	9.33 E-01	3.40 E-01
APOB	rs515135	C/T	4.80 E-10	LDL-C, TC	8.38 E-92	+	3.14 E-109	+	9.13 E-03	-	6.66 E-04	+	6.43 E-01	1.00 E-01	3.54 E-01	-	9.68 E-01	+	6.63 E-01	+	9.74 E-01	-	8.84 E-01	-	1.50 E-01	-	9.95 E-01	3.20 E-01
ABCG5/ ABCG8	rs6544713	T/C	8.72 E-10	LDL-C, TC	1.17 E-44	+	2.37 E-47	+	1.61 E-01	-	4.22 E-03	+	8.99 E-01	8.98 E-01	3.73 E-01	-	4.50 E-01	+	7.91 E-02	+	2.00 E-01	+	5.32 E-01	+	2.27 E-01	-	1.24 E-01	4.40 E-01
GGCX/ VAMP10/ VAMP8	rs1561198	T/C	4.48 E-09	None	7.48 E-02	-	1.30 E-01	-	4.63 E-01	-	8.19 E-01	+	9.85 E-01	9.52 E-01	6.27 E-02	-	8.46 E-01	-	9.67 E-01	+	1.71 E-01	+	9.84 E-01	-	5.55 E-01	+	1.25 E-01	3.50 E-01
ZEB2-AC074093.1	rs2252641	C/T	3.66 E-08	None	7.29 E-01	+	2.31 E-01	+	1.31 E-01	+	1.96 E-01	-	1.90 E-02	3.14 E-02	8.17 E-01	+	5.61 E-01	-	6.58 E-01	+	3.84 E-01	-	6.86 E-01	-	3.84 E-03	-	8.41 E-01	9.00 E-01
EDNRA	rs1878406	T/C	1.32 E-13*	None	3.76 E-01	+	4.93 E-01	+	8.87 E-01	+	7.21 E-01	-	8.82 E-02	8.08 E-01	1.04 E-01	-	3.98 E-01	-	1.08 E-01	-	3.87 E-01	+	5.32 E-01	-	NA	NA	6.22 E-01	3.50 E-01
GUCY1A3	rs7692387	G/A	4.57 E-09	DBP	2.94 E-01	+	8.27 E-01	+	7.63 E-01	+	2.47 E-01	+	6.05 E-03	3.35 E-05	1.85 E-01	+	6.67 E-01	-	8.27 E-01	-	6.24 E-01	-	9.67 E-01	-	5.20 E-02	+	6.21 E-01	9.50 E-01
SLC22A4/ SLC22A5	rs273909	G/A	1.43 E-08	None	3.04 E-03	+	3.60 E-04	+	2.89 E-02	-	1.94 E-02	+	8.80 E-01	4.71 E-01	2.45 E-01	-	8.27 E-01	+	9.70 E-01	+	2.70 E-01	+	8.15 E-01	+	6.87 E-01	+	4.50 E-01	2.30 E-01
KCNK5	rs10947789	T/C	1.63 E-08	None	1.76 E-01	-	5.05 E-01	-	2.55 E-02	-	8.80 E-01	+	6.24 E-01	1.70 E-02	6.99 E-01	-	3.60 E-01	+	7.70 E-02	-	5.54 E-01	+	4.94 E-01	+	3.92 E-01	-	1.92 E-01	2.70 E-02
PLG	rs4252120	T/C	5.00 E-09	None	5.78 E-01	+	3.39 E-01	+	6.65 E-01	-	9.10 E-01	+	7.35 E-01	7.32 E-01	2.03 E-01	+	6.91 E-01	+	6.89 E-01	-	9.01 E-01	+	3.94 E-01	+	9.31 E-01	-	6.75 E-02	6.80 E-02
HDAC9	rs2023938	T/C	3.59 E-10*	None	5.17 E-01	+	5.23 E-01	+	9.77 E-01	-	6.24 E-01	-	1.41 E-02	7.32 E-01	8.44 E-01	-	8.05 E-01	-	7.82 E-02	-	5.24 E-01	-	8.19 E-01	-	7.05 E-01	+	9.29 E-01	7.00 E-01
LPL	rs264	G/A	5.06 E-09	HDL-C, TG	6.73 E-01	+	4.55 E-01	+	7.02 E-48	-	5.66 E-46	+	9.84 E-01	7.94 E-01	1.29 E-01	+	6.60 E-01	+	6.23 E-01	+	5.30 E-01	-	5.02 E-01	+	5.54 E-02	+	2.10 E-02	9.80 E-01
TRIB1	rs2954029	A/T	4.53 E-08	TG, TC, LDL-C, HDL-C	1.17 E-35	+	5.36 E-29	+	4.85 E-18	-	3.29 E-55	+	3.75 E-01	7.53 E-01	6.37 E-01	-	6.40 E-01	+	1.34 E-02	+	9.50 E-01	+	8.71 E-01	+	9.57 E-01	+	3.59 E-01	3.50 E-01
FLT1	rs9319428	A/G	1.01 E-08	None	4.77 E-01	+	6.46 E-01	-	1.02 E-01	+	5.87 E-01	+	8.39 E-01	1.05 E-01	6.69 E-01	-	9.35 E-01	+	7.80 E-01	-	4.22 E-01	+	8.52 E-01	+	9.26 E-01	+	3.76 E-01	3.20 E-03
FURIN/ FES	rs17514846	A/C	4.49 E-10	SBP	7.19 E-01	+	1.37 E-01	+	3.14 E-01	+	1.02 E-01	-	1.17 E-05	4.32 E-03	5.02 E-01	+	5.64 E-01	-	8.52 E-01	+	6.04 E-01	-	5.26 E-01	-	1.33 E-01	+	1.16 E-01	5.70 E-02
Established Loci																												
MIA3	rs17465637	C/G	n/a	None	2.38 E-01	-	1.99 E-01	-	5.12 E-01	+	7.72 E-02	-	5.90 E-01	9.82 E-01	3.33 E-01	-	3.27 E-01	+	NA	NA	3.36 E-01	+	4.47 E-01	+	3.24 E-01	+	2.50 E-01	2.10 E-01

Nature Genetics: doi:10.1038/ng.2480

PCSK9	rs11206510	T/C	1.79 E-05	TC, LDL-C	3.54 E-20	+	5.23 E-20	+	1.90 E-01	-	2.07 E-04	+	8.63 E-01	2.78 E-01	5.93 E-01	-	1.45 E-01	-	1.98 E-01	-	3.19 E-01	-	1.07 E-01	-	4.51 E-02	+	5.53 E-01	1.50 E-01
PPAP2B	rs17114036	C/T	5.80 E-12	None	8.09 E-01	+	7.47 E-01	-	2.54 E-02	+	2.92 E-01	-	4.01 E-01	7.19 E-01	9.40 E-01	-	5.64 E-01	-	8.13 E-01	-	6.84 E-01	-	5.81 E-01	-	NA	NA	5.08 E-01	8.60 E-01
SORT1	rs599839	A/G	3.85 E-15	LDL-C, TC, HDL-C	4.12 E-130	+	2.94 E-168	+	5.56 E-07	-	4.43 E-02	+	8.91 E-01	6.59 E-01	1.25 E-01	-	2.65 E-01	-	1.11 E-01	-	8.68 E-01	+	2.15 E-01	-	6.11 E-02	-	4.71 E-02	1.70 E-01
WDR12	rs2351524	T/C	1.88 E-17	None	4.51 E-04	-	2.49 E-03	-	4.55 E-01	-	4.82 E-02	-	9.49 E-01	1.08 E-01	6.85 E-02	-	1.90 E-01	+	5.50 E-01	+	1.03 E-01	+	2.92 E-01	+	9.60 E-02	-	1.99 E-02	3.10 E-01
MRAS	rs9818870	T/C	2.62 E-09	None	1.25 E-01	+	3.04 E-01	+	6.58 E-01	-	3.50 E-01	+	3.92 E-02	2.19 E-02	5.46 E-01	+	1.65 E-01	+	1.54 E-01	+	3.25 E-01	+	1.83 E-01	+	5.96 E-01	+	3.85 E-01	3.00 E-01
ANKS1A	rs12205331	C/T	4.18 E-05	None	8.00 E-01	+	6.43 E-01	+	8.84 E-04	-	5.29 E-03	+	2.85 E-01	9.20 E-02	3.28 E-01	+	7.37 E-01	+	8.98 E-01	+	8.12 E-01	+	5.09 E-01	+	2.36 E-01	+	1.28 E-01	7.60 E-01
ANKS1A	rs3822921	A/G	6.17 E-05	TC, Height, HDL-C	2.61 E-06	-	2.75 E-04	-	1.65 E-05	-	7.68 E-01	-	6.85 E-01	4.09 E-01	7.97 E-01	+	6.07 E-02	+	2.56 E-01	-	1.57 E-01	+	1.39 E-01	+	3.15 E-01	+	1.13 E-02	3.90 E-01
ANKS1A	rs12525532	T/C	4.76 E-04	FI, H-IR	1.32 E-03	-	1.52 E-02	-	1.02 E-03	-	2.14 E-02	+	6.01 E-01	7.64 E-01	2.41 E-03	+	1.20 E-05	+	4.45 E-01	-	1.21 E-02	+	1.83 E-05	+	1.95 E-01	+	1.34 E-01	4.80 E-01
PHACTR1	rs9369640	A/C	7.53 E-22	None	1.55 E-01	-	1.75 E-01	-	4.78 E-01	+	1.02 E-01	-	1.72 E-02	2.45 E-02	2.76 E-01	+	8.44 E-01	-	5.01 E-04	-	5.38 E-01	-	8.78 E-01	-	6.38 E-01	+	4.78 E-02	7.10 E-01
SLC22A3/ LPAL2/ LPA	rs3120139	A/G	2.23 E-07	TC, LDL-C	5.24 E-07	+	4.03 E-05	+	6.39 E-01	-	4.29 E-04	+	9.21 E-01	3.40 E-01	5.45 E-02	+	9.22 E-01	+	7.82 E-01	-	1.48 E-01	-	8.67 E-01	+	3.88 E-01	-	2.08 E-01	4.40 E-02
SLC22A3/ LPAL2/ LPA	rs2048327	C/T	6.86 E-11	None	5.95 E-04	+	4.07 E-03	+	4.89 E-01	-	1.49 E-02	+	4.72 E-01	4.94 E-01	3.97 E-02	+	4.32 E-01	-	4.12 E-01	-	6.41 E-02	-	9.15 E-01	-	7.24 E-01	-	1.18 E-02	3.40 E-01
TCF21	rs2327429	T/C	3.04 E-11	None	2.38 E-03	-	7.18 E-03	-	2.99 E-01	-	8.57 E-01	-	2.76 E-01	1.55 E-01	7.81 E-01	+	1.57 E-01	-	5.54 E-01	+	4.48 E-02	-	1.59 E-01	-	9.79 E-01	-	1.45 E-01	1.60 E-01
ZC3HC1	rs11556924	C/T	6.74 E-17	DBP	1.58 E-01	+	2.93 E-02	+	3.77 E-03	-	1.81 E-01	+	1.27 E-04	1.79 E-05	8.92 E-02	+	3.78 E-01	+	8.54 E-02	+	5.63 E-01	-	4.16 E-01	+	3.52 E-01	+	6.28 E-02	8.30 E-01
ABO	rs579459	C/T	2.66 E-08	LDL-C, TC	4.03 E-20	+	2.36 E-21	+	8.39 E-02	+	1.49 E-02	-	1.91 E-01	1.03 E-03	4.54 E-03	+	5.86 E-01	+	6.30 E-01	+	1.07 E-01	-	5.27 E-01	+	3.40 E-01	+	7.75 E-01	3.40 E-01
CDKN2BAS	rs1333049	C/G	1.39 E-52	None	8.13 E-03	-	1.56 E-01	-	2.62 E-01	-	7.11 E-01	-	9.93 E-01	1.75 E-01	4.16 E-02	+	6.61 E-01	+	3.97 E-02	+	3.40 E-01	+	2.77 E-01	+	5.17 E-02	+	6.36 E-01	1.30 E-01
CXCL12	rs2047009	G/T	1.59 E-09	None	8.90 E-01	+	6.75 E-01	+	6.78 E-01	+	6.58 E-01	-	6.74 E-01	2.13 E-02	8.50 E-01	+	3.90 E-01	+	6.68 E-01	+	6.70 E-01	+	3.82 E-01	+	5.39 E-01	+	7.22 E-01	1.80 E-01
CXCL12	rs687175	T/C	2.31 E-09	None	1.94 E-01	-	5.23 E-01	-	7.38 E-01	-	1.41 E-01	-	5.12 E-01	1.23 E-01	7.53 E-02	-	1.56 E-01	-	9.93 E-01	+	5.22 E-01	-	1.29 E-01	-	6.88 E-01	-	5.08 E-01	4.20 E-02
CYP17A1/ CNNM2/ NT5C2	rs12413409	G/A	6.26 E-08	SBP, DBP, BMI	1.24 E-01	+	7.64 E-01	+	1.76 E-01	+	4.37 E-01	+	2.00 E-09	8.81 E-06	8.68 E-01	-	9.78 E-01	-	3.55 E-01	+	7.36 E-01	-	9.56 E-01	-	7.35 E-01	-	4.75 E-05	5.00 E-02
KIAA1462	rs2505083	C/T	1.35 E-11	None	3.09 E-01	+	6.03 E-01	+	5.46 E-01	-	6.27 E-02	+	3.93 E-01	1.73 E-01	4.44 E-01	+	3.63 E-01	+	7.50 E-01	+	8.33 E-01	+	3.28 E-01	+	3.17 E-01	+	6.96 E-01	8.70 E-01

LIPA	rs11203042	T/C	6.08 E-06	None	6.24 E-01	-	7.14 E-01	-	6.34 E-01	+	5.22 E-02	-	6.88 E-01	5.70 E-01	4.95 E-01	+	2.60 E-01	-	3.08 E-01	-	1.04 E-01	-	1.74 E-01	-	3.72 E-01	-	9.58E-01	3.80 E-01
LIPA	rs2246833	T/C	9.49 E-06	None	8.70 E-01	-	6.59 E-01	-	1.58 E-01	+	5.22 E-01	-	3.91 E-01	6.08 E-01	3.29 E-01	+	2.81 E-01	-	1.70 E-01	-	2.61 E-01	-	4.83 E-01	-	6.33 E-01	-	1.77 E-01	2.90 E-01
PDGFD	rs974819	T/C	3.55 E-11	None	3.42 E-01	+	2.71 E-01	+	3.83 E-01	-	3.19 E-01	+	9.91 E-01	7.57 E-01	2.32 E-02	+	4.97 E-01	+	5.29 E-01	+	3.43 E-01	-	4.88 E-01	+	4.04 E-01	+	7.23 E-01	8.10 E-01
ZNF259/ APO5A/ APOA1	rs9326246	C/G	1.51 E-07	TG, TC, HDL-C, LDL-C	7.09 E-32	+	9.99 E-15	+	1.79 E-19	-	4.7 E-124	+	3.18 E-01	6.71 E-01	3.24 E-02	+	7.90 E-01	+	3.35 E-01	+	8.00 E-01	-	4.69 E-01	+	2.31 E-01	+	2.96 E-01	5.20 E-01
SH2B3	rs3184504	T/C	5.44 E-11	DBP, TC, SBP, LDL-C, HDL-C	2.69 E-11	-	1.73 E-09	-	4.97 E-06	-	1.85 E-01	+	1.69 E-09	2.33 E-14	4.09 E-01	+	7.51 E-01	+	1.12 E-01	+	8.57 E-01	+	5.20 E-01	+	3.37 E-01	+	1.30 E-04	9.00 E-01
COL4A1/ COL4A2	rs9515203	T/C	5.85 E-12	None	2.14 E-01	-	4.57 E-01	-	6.31 E-01	+	1.17 E-01	-	1.45 E-03	1.12 E-02	3.42 E-01	-	2.58 E-01	+	6.70 E-02	-	1.14 E-01	+	4.16 E-01	+	7.55 E-02	-	3.64 E-02	2.20 E-01
COL4A1/ COL4A2	rs3809346	A/G	2.56 E-12	None	1.55 E-02	-	1.26 E-02	-	5.79 E-01	-	3.61 E-01	+	6.01 E-01	7.54 E-01	1.04 E-01	-	4.64 E-01	+	4.73 E-02	+	5.30 E-02	+	4.69 E-01	+	3.87 E-01	-	1.71 E-01	2.60 E-01
HHIPL1	rs2895811	C/T	4.08 E-10	None	7.95 E-02	+	5.41 E-02	+	7.47 E-01	+	8.84 E-01	+	1.78 E-01	7.62 E-01	2.12 E-01	-	8.65 E-01	+	9.53 E-01	+	6.27 E-01	+	9.76 E-01	-	1.40 E-01	-	7.73 E-01	2.20 E-01
ADAMTS7	rs3825807	A/G	n/a	None	6.92 E-01	+	7.98 E-01	-	5.96 E-01	+	8.38 E-01	-	6.89 E-01	2.46 E-01	4.77 E-01	-	5.20 E-01	-	4.65 E-01	-	4.65 E-01	-	4.85 E-01	-	NA	NA	2.40 E-01	1.90 E-01
ADAMTS7	rs11072794	T/C	1.10 E-12	None	9.55 E-01	-	3.31 E-01	-	3.41 E-01	+	6.82 E-01	-	2.79 E-01	1.69 E-03	5.07 E-01	+	8.80 E-01	-	2.51 E-01	+	7.21 E-01	-	9.89 E-01	+	4.86 E-02	+	2.91 E-01	5.50 E-01
RAI1/ PEMPT/ RASD1	rs12936587	G/A	1.24 E-09	WHR	2.26 E-01	+	3.23 E-01	+	1.69 E-02	-	9.59 E-03	+	8.70 E-01	6.13 E-01	4.50 E-01	-	8.20 E-01	-	5.21 E-01	+	4.09 E-01	+	7.11 E-01	-	5.80 E-01	+	3.09 E-01	2.86 E-05
SMG6	rs2281727	G/A	7.83 E-09	None	9.72 E-03	-	9.18 E-02	-	1.24 E-02	-	8.60 E-01	-	3.94 E-01	6.75 E-01	3.60 E-01	-	3.33 E-01	-	6.34 E-01	-	5.39 E-01	-	3.02 E-01	-	7.21 E-01	-	2.16 E-02	1.40 E-01
UBE2Z	rs15563	G/A	9.37 E-06	Height	8.69 E-01	+	3.26 E-01	+	1.15 E-01	-	7.13 E-01	-	7.22 E-01	1.41 E-01	9.56 E-01	+	1.13 E-01	-	3.84 E-01	+	7.59 E-02	-	2.31 E-01	-	1.31 E-03	+	6.22 E-01	8.10 E-02
LDLR	rs1122608	G/T	6.33 E-14	LDL-C, TC	4.59 E-26	+	1.24 E-33	+	2.87 E-01	-	3.64 E-01	+	9.46 E-01	6.65 E-01	6.90 E-01	-	4.61 E-01	-	4.10 E-02	+	8.44 E-01	-	5.19 E-01	-	7.23 E-01	-	1.28 E-01	3.70 E-01
APOE	rs2075650	G/A	5.86 E-11**	LDL-C, TC, TG, HDL-C	1.33 E-84	+	3.76 E-110	+	1.10 E-16	-	1.31 E-19	+	4.86 E-01	9.52 E-01	6.35 E-01	-	9.49 E-01	-	1.24 E-02	-	7.73 E-01	-	9.35 E-01	-	8.54 E-01	+	2.37 E-02	2.50 E-02
KCNE2	rs9982601	T/C	7.67 E-17	None	8.64 E-01	+	4.13 E-01	-	8.55 E-02	+	2.94 E-01	+	1.11 E-01	2.59 E-01	8.75 E-01	+	5.07 E-01	-	9.80 E-01	-	6.96 E-01	-	9.71 E-01	-	2.00 E-01	-	4.27 E-01	2.10 E-01

SNP : Effect/ Non Effect Allele, CAD P-value (for Stage 1 and 2 analysis, unless * Stage 1, 2 and 3 combined P, ** Stage 2 results only, n/a Not on Metabochip), Risk factor summary : Summary of significant risk factors (in order of significance), TC : Total Cholesterol level, LDL-C : Low Density Lipoprotein Cholesterol level, HDL-C : High Density Lipoprotein Cholesterol level, TG : Triglyceride level, SBP : Systolic Blood Pressure, DBP : Diastolic Blood Pressure, FG : Fasting glucose level, FI : Fasting Insulin level, 2hG : 2 hour glucose level, H-B : HOMA-B level, H-IR : HOMA-IR level, T2D : Type 2 Diabetes, BMI : Body Mass Index, WHR : Waist Hip Ratio (BMI adjusted), n values quoted for each trait are the average of the 51 SNPs within the table. Red shaded squares highlight P-values less than the Bonferroni significance level of 6.5E-05 which was based on 51 SNPs and 15 traits.

Nature Genetics: doi:10.1038/ng.2480

Supplementary Table 9: SNPs at an FDR≤5% and LD threshold of $r^2 < 0.2$ used in estimating heritability
Provided as separate attachment

Supplementary Table 10: Network molecules

Provided as separate attachment

Nature Genetics: doi:10.1038/ng.2480

1. Study characteristics

Descriptions of all studies in stage 1 (CARDIoGRAM discovery) are given in ref 1[1]. **Supplementary Table 2a/b** provides an overview of the Stage 2 and 3 studies. A more detailed description of each study is given below:

Stage 2 Studies

ADVANCE

The ADVANCE (Atherosclerotic Disease, Vascular Function, and Genetic Epidemiology) study is a case-control investigation of genetic and nongenetic determinants of CAD and mode of CAD presentation. All study participants have been identified and recruited from the membership of Kaiser Permanente of Northern California (KPNC), a large integrated health care delivery system in the San Francisco Bay area and surrounding counties The ADVANCE Study was approved by the Institutional Review Board at Stanford and the Kaiser Foundation Research Institute. All subjects gave written informed consent
The phenotype for cases was program members aged 45 years or older for males and 55 years or older for females at the time of their incident clinical coronary artery disease event between 28 October 2001 and 31 December 2003. For MI, patients had to have positive cardiac enzymes in the electronic databases as well as a primary discharge diagnosis of myocardial infarction (ICD 9 code 410). For stable angina, patients had to have diagnosis of stable angina (ICD9 code 413.x) in the electronic outpatient databases followed by confirmation from both the primary care physician and the patient of the recent onset of incident stable and typical angina. Two cases from the early onset CAD cohort (age of onset of CAD < 45 years for men and < 55 years for women) not included in prior ADVANCE GWAS were also genotyped. The control phenotype was defned as Program members aged 60 to 69 as of January 6, 2001, with no history of cardiovascular disease, cancer (other than nonmelanoma skin cancer), renal failure, liver cirrhosis, dementia, or human immunodeficiency virus/ acquired immunodeficiency syndrome or with a source of care greater than 50 miles (80.47 km) from the clinic used for data collection were recruited[2-5].

AMC-PAS/ SANGUIN

The AMC-PAS(Academic Medical Center Amsterdam Premature Atherosclerosis Study) study consists of patients referred to the Academic Medical Center in Amsterdam, which is specialised in premature CAD, for investigation of symptomatic coronary and/or peripheral arterial disease. Case definition was symptomatic CAD before the age of 51 years, defined as MI, coronary revascularization, or evidence of at least 70% stenosis in a major epicardial artery
Controls were blood donors from the north-west region of the Netherlands; recruited at routine Sanquin Blood Bank donation sessions. More than 95% of the controls are from the same region as the cases of the AMC-PAS cohort[6].

Angio-Lueb/KORA F3

The Lübeck Registry of Structural Heart Disease /KORA (Kooperative Gesundheitsforschung in der Region Augsburg) survey S3/F3 datasets included cases selected from consecutive patients referred for coronary angiography, which were classified as CAD/MI based on the coronary angiogram; cases were < 65 y in males or < 70 y in females. The KORA survey contains representative samples from the general population living in or near Augsburg, Germany and were conducted between 1994 and 2004[1,7].

Cardiogenics

The Cardiogenics Study recruited patients from five European centres. Patients from Germany(Lubeck and Regensburg) and the UK (Leicester) were under the age of 65 with a confirmed primary MI within the

Nature Genetics: doi:10.1038/ng.2480

preceding 3-36 months. Exclusion criteria were (*i*) a history of diabetes mellitus based on plasma glucose >7.0 mmol/l or HbA1C > 7.0 (*ii*) renal insufficiency, (*iii*) patients not on statin therapy, (*iv*) CRP level >10mg/dl, (v) patients not fasting at the time of blood sampling or (*vi*) current smokers. The French (Paris) cohort comprised patients aged 33 to 87, recruited within the BAAAC (Banque d'ADN et d'ARN de patients présentant une Athérosclérose Coronarienne) study, with symptoms of acute coronary syndrome who had one stenosis >50% diagnosed in at least one major coronary artery.
Controls comprised healthy individuals (aged 32 to 65 years) recruited in the UK(Cambridge) who were blood donors recruited as part of the Cambridge Bioresource[8].

DILGOM

Samples were collected as part of the Dietary, Lifestyle, and Genetic determinants of Obesity and Metabolic syndrome (DILGOM) study. Study participants were aged 25–74 years and were drawn from five geographical areas of Finland. All CAD cases are incident definite or possible MI or coronary death, or unstable angina during follow-up, coronary revascularization during follow-up, documented MI at baseline, or an unclassifiable coronary death during follow-up. MI cases had definite myocardial infarction. Non-cases were selected from the same population-based longitudinal cohort study[9].

DUKE

The Duke CATHGEN biorepository consists of subjects recruited sequentially through the cardiac catheterization laboratories at Duke University Medical Center (Durham, NC, USA). After informed consent was obtained, blood was drawn from the femoral artery at the time of arterial access for catheterization. Clinical data were provided by the Duke Databank for Cardiovascular Disease (DDCD), a database of patients undergoing catheterization at Duke University since 1969. Follow-up data, including occurrence of myocardial infarction (MI) and death, were collected at 6 months after catheterization and annually thereafter. Vital status was confirmed through the National Death Index. The Duke Institutional Review Board approved the protocols for CATHGEN. Subjects (case and control), were excluded if they had severe pulmonary hypertension or congenital heart disease or were diabetic.Cases had at least one epicardial coronary vessel with at least 50% blockage, age of onset was no older than 65 for women and 55 for men. Controls were required to be at least 50 years old and have no epicardial coronary vessel with greater than 30% blockage. Controls with a history of ICC/PCI, CABG, MI or transplant were excluded[10].

EGCUT

The EGCUT (Estonian Genome Center of University of Tartu) study recruited samples from Estonia. Cases were study participants who reported following cardiovascular disease events (ICD10 I20-I26) when recruited. Controls were participants in the same study that did not those cardiovascular disease events[11].

EPIC

The EPIC (European Prospective Study into Cancer and Nutrition) study sub-cohorts from the UK were used, subjects were collected in collaboration with general practicioners, mainly in Cambridgeshire and Norfolk. Cases were individuals who developed a fatal or non-fatal CAD during an average follow-up of 11 years, until June 2006. Participants were identified if they had a hospital admission and/or died with CAD as the underlying cause. CAD was defined as cause of death codes ICD9 410-414 or ICD10 I20-I25, and hospital discharge codes ICD10 I20.0, I21, I22 or I23 according to the International Classification of Diseases, 9th and 10th revisions. Controls were study participants who remained free of any cardiovascular disease during follow-up (defined as ICD9 401-448 and ICD10 I10-I79). Controls were matched to each case by sex, age (within 5 years), and time of enrolment (within 3 months).

FGENTCARD

Nature Genetics: doi:10.1038/ng.2480

The FGENTCARD (Functional Genomic Diagnostic Tools for Coronary Artery Disease) study subjects consisted of 6517 individuals who underwent cardiac catheterization following a single consistent and stringent recruitment protocol between August, 2007 and March 2011 at several hospitals in Lebanon. Catheterization was prompted for myocardial infarction (MI) (12.5%) as diagnosed by electrocardiogram and high troponin levels, unstable angina (27.5%), or other reasons, such as stable angina, or heart failure, or reversible ischemia by stress testing (59.9%). All patients underwent coronary catheterization by Judkins technique. The four main coronary arteries: the left main artery (LMCA), the left anterior descending artery (LAD), the left circumflex artery (LCx), and the right coronary artery (RCA) were visualized from different angles by angiography. The extent of stenosis in these vessels was assessed and recorded by percentage. Cases were defined as follows: Mildly diseased if at least one of the four vessels has less than 50% stenosis, severly diseased if any of the four vessels has ≥50% stenosis. Controls are subjects with no stenosis in the 4 main vessels

FRISCII/GLACIER

The FRISC II, (Fragmin and Fast Revascularization during Instability in Coronary Artery Disease) study was a prospective, randomised, multicentre trial (58 hospitals in Sweden, Norway and Denmark) with parallel groups to compare invasive and non-invasive treatments. FRISCII patients were eligible for inclusion if they had symptoms of ischaemia that were increasing or occurring at rest, or that warranted the suspicion of acute myocardial infarction, with the last episode within 48 h before the start of dalteparin or standard heparin treatment. Myocardial ischaemia had to be verified by electrocardiography (ST depression ≥0·1 mV or T-wave inversion ≥0·1 mV) or by raised biochemical markers (creatine kinase [CK]-MB >6 ug/L, troponin-T >0·10 ug/L, qualitative troponin-T test positive, or catalytic activity of CK, CK-B, or CK MB higher than the local diagnostic limit for myocardial infarction). Exclusion criteria were raised risk of bleeding episodes, anaemia, or indication for or treatment in the past 24 h with thrombolysis, angioplasty in the past 6 months, being on a waiting list for coronary revascularisation, other acute or severe cardiac disease, renal or hepatic insufficiency, known clinically relevant osteoporosis, other severe illness, hypersensitivity to randomised drugs, anticipated difficulties with cooperation or participation in this or another clinical trial[12]. The GLACIER (Gene x Lifestyle interactions And Complex traits Involved in Elevated disease Risk) cohort was used in these analyses as a comparison cohort for FRISCII. GLACIER is a subset of the Västerbottens Intervention Project, a population-based cohort from northern Sweden. The GLACIER cohort is comparable to the full VIP cohort in demographic, anthropometric, and lifestyle characteristics[13].

GoDARTS

The GoDARTS (Genetics of Diabetes Audit and Research in Tayside Scotland) study is a joint initiative of the Department of Medicine and the Medicines Monitoring Unit (MEMO) at the University of Dundee, the diabetes units at three Tayside healthcare trusts (Ninewells Hospital and Medical School, Dundee; Perth Royal Infirmary; and Stracathro Hospital, Brechin), and a large group of Tayside general practitioners with an interest in diabetes care. Cases were a first-ever CAD event, defined as fatal and non-fatal myocardial infarction, unstable angina or coronary revascularisation. Controls were free of coronary artery disease, stroke and peripheral vascular disease[14].

HPS

The MRC/BHF Heart Protection Study (HPS) was a large UK-based cholesterol-lowering trial involving participants with a history of MI, unstable or stable angina, coronary artery bypass grafting, or angioplasty (as well as patients with prior history of stroke or hypertension). HPS CAD cases were compared with population controls from the UK Twins Study and the WTCCC2 National Blood Service Collections[15].

ITH

Nature Genetics: doi:10.1038/ng.2480

The INTERHEART Study (ITH) used worldwide cases and controls of European ethnicity. Cases were incident acute MI, presenting to a hospital within 24 hours of symptom onset, controls were age and sex matched hospital and community based, with no previous diagnosis of heart disease or history of exertional chest pain[16].

LOLIPOP

The LOLIPOP CHD-GEN (London Life Sciences Population) study includes individuals of Indian Asian descent. CHD cases were recruited from the Ealing and Hammersmith hospitals. CAD was defined as a history of MI or coronary artery revascularization (CABG or percutaneous coronary intervention (PCI)), or angiographically confirmed coronary artery stenosis greater than 50%. Clinical diagnosis of MI is based on two out of three of: 1. Chest pain, 2. Raised cardiac enzymes, 3. ECG changes. Controls were Indian Asian men and women, aged 35 to 75 years, without diagnosis or history of CAD and recruited from the lists of 58 General Practitioners in West London. All participants gave written consent for participation in genetic studies and the protocol was approved by the local Research Ethics Committee[17-19].

LURIC/EMIL

The LURIC (LUdwigshafen RIsk and Cardiovascular Health) study inclusion criteria were: German ancestry, clinical stability except for acute coronary syndromes, and the availability of a coronary angiogram. Cases were included with angiographically confirmed CAD (at least one coronary vessel with a stenosis > 50%) The study was approved by the Ethics Committee at the “Aerztekammer Rheinland-Pfalz”. Informed written consent was obtained from all participants[20].
The EMIL (Echinococcus Multilocularis and Internal Diseases in Leutkirch study) study is a GerBS control series that consists of healthy, unrelated blood donors recruited between May-July 2004 from the southwestern area of Germany / EMIL controls include population-based non-cases subjects[21].

METSIM

METSIM (METabolic Syndrome In Men) is a population-based cross-sectional study that includes subjects, aged from 45 to 70 years which were randomly selected from the population register of the town of Kuopio in eastern Finland (population 95,000). CAD cases were Angiography-confirmed CAD, myocardial infarction, balloon angioplasty or coronary bypass. Controls were selected from the same population-based sample and were free from MI, coronary angiography, balloon angioplasty, cerebral infarction, cerebral hemorrhage, or any leg operation. The study was approved by the ethics committee of the University of Kuopio and Kuopio University Hospital, and it was in accordance with the Helsinki Declaration[22].

MORGAM

The MORGAM (MONICA, Risk, Genetics, Archiving, and Monograph)Study, has harmonized data from the prospective follow-up of population cohorts in several countries. Finland (FIN): Southern Finland, North Karelia, Kuopio Province, Oulu Province, Turku/Loimaa, Helsinki, France (FRA): Lille, Strasbourg, Toulouse, Germany(GER): Augsburg, Italy(ITA): Brianza and the United Kingdom (UNK): Belfast. All CAD cases are incident definite or possible MI or coronary death, or unstable angina during follow-up, Coronary revascularization during follow-up, Documented MI at baseline, or an unclassifiable coronary death during follow-up. MI cases were:definite myocardial infarction. Controls are 1:1 matched for cases (by age, sex, and region). They are participants who remained free of any cardiovascular disease at the age when the matched case had the first event[23,24].

OHGS

The OHGS (Ottawa Heart Genomics Study) cases had at least one of myocardial infarction, coronary artery bypass graft, percutenaeous intervention or a stenosis of at least 50% in at least one epicardial vessel. Diabetic cases and cases aged greater than 55 for men or 65 for women were excluded. Controls were

Nature Genetics: doi:10.1038/ng.2480

either asymptomatic for cardiovasular disease or had had a CTA or angiogram demonstrating no stenosis of greater than 50%. Controls were required to be at least 65 years old for men and 70 years old for women at the time of recruitment[25].

PIVUS

Individuals within the PIVUS (Prospective Investigation of the Vasculature in Uppsala Seniors) cohort study who developed a fatal or non-fatal myocardial infarction or unstable angina during follow-up were included as cases. Participants were identified as having CHD if they had a hospital admission with CHD as the primary cause of hospitalization and/or died with CAD as the underlying cause. CHD was defined as acute myocardial infarction (ICD-8 and ICD-9 code 410, ICD-10 codes I21-I22) or unstable angina (ICD-8 code 411, ICD-9 code 411B, ICD-10 code I20.0). The positive predictive values (i.e. validity) of the CHD diagnosis in the Swedish hospital discharge register has been demonstrated to be at least 95% when only primary diagnoses are considered. Non-cases were selected from the same longitudinal, community-based cohort study[26].

PopGen

The PopGen Population-based northern German cross-sectional study comprises unrelated CAD patients recruited in Schleswig-Holstein, through the population-based PopGen biobank. Cases had significant CAD (at least a 70% stenosis in one major coronary vessel) with age of onset < 55 y. Population based controls are participants who remained free of any cardiovascular disease[27].

PROCARDIS

The PROCARDIS (European collaborative study of the genetics of precocious coronary artery disease) study is a multi-centre case-control study in which CAD cases and controls were recruited from United Kingdom, Italy, Sweden and Germany. Cases were defined as symptomatic CAD before age 66 years and 80% of cases also had a sibling in whom CAD had been diagnosed before age 66 years. CAD was defined as clinically documented evidence of myocardial infarction (MI) (80%), coronary artery bypass graft (CABG) (10%), acute coronary syndrome (ACS) (6%), coronary angioplasty (CA) (1%) or stable angina (hospitalization for angina or documented obstructive coronary disease) (3%). The cases included 2,136 cases who were half or full siblings. PROCARDIS controls had no personal or sibling history of CAD before age 66 years. PoBI and UK Twin study are population-based controls that were not screened for CAD[28].

PROMIS

PROMIS (The Pakistan Risk Of Myocardial Infarction Study) is an ongoing case-control study of acute myocardial infarction (MI) in urban Pakistan, which by mid-2009 included 5,500 MI cases and 5,500 controls. Cases have typical ECG characteristics, a positive troponin test, and MI symptoms within the previous 24 hours. Controls are individuals frequency-matched to cases by sex and age (in 5 year bands) and identified in the same hospitals as the index cases. Controls have been recruited in the following order of priority: (i) visitors of patients attending the out-patient department; (ii) patients attending the out-patient department for routine non-cardiac complaints, or (iii) non-blood related visitors of index MI cases. A locally-piloted and validated epidemiological questionnaire has been administered to participants by medically qualified research officers that seeks >200 items of information in relation to: ethnicity (eg, personal and paternal ethnicity, spoken language, place of birth and any known consanguinity); demographic characteristics; lifestyle factors (eg, tobacco and alcohol consumption, dietary intake and physical activity); personal and family history of cardiovascular disease; and medication usage. PROMIS has received approval by the relevant research ethics committee of each of the institutions involved in participant recruitment. Informed consent has been obtained from each participant recruited into the study, including consent to use the samples in genetic, biochemical and other analyses[29].

SCARF/SHEEP

Nature Genetics: doi:10.1038/ng.2480

SHEEP/SCARF are Swedish population based case-control studies. Cases are patients with a first confirmed myocardial infarction, controls have no history of myocardial infarction.

STR

The STR (Swedish Twin Registry) registry includes nearly 25,000 pairs of twins of the same sex born in Sweden between 1886 and 1958. Individuals were included within this cohort study who developed a fatal or non-fatal myocardial infarction or unstable angina during follow-up. Participants were identified as having CHD if they had a hospital admission with CHD as the primary cause of hospitalization and/or died with CAD as the underlying cause. CHD was defined as acute myocardial infarction (ICD-8 and ICD-9 code 410, ICD-10 codes I21-I22) or unstable angina (ICD-8 code 411, ICD-9 code 411B, ICD-10 code I20.0). The positive predictive values (i.e. validity) of the CHD diagnosis in the Swedish hospital discharge register has been demonstrated to be at least 95% when only primary diagnoses are considered. Non-cases were selected from the same longitudinal, community-based cohort study. This study was approved by the Ethics Committee of the Karolinska Institute and the Swedish National Data Inspection Authority. All subjects gave informed consent[30].

THISEAS

The THISEAS (The Hellenic Study of Interactions between Snps and Eating in Atherosclerosis Susceptibility) study participants were recruited from 3 hospitals found in the area of Athens. Cases were subjects with a first-ever MI before age of 70 yrs presenting with either ACS or stable CAD defined as >50% stenosis in at least one of the three main coronary vessels assessed by coronary angiography. ACS was defined as acute MI or unstable angina corresponding to class III of the Braunwald classification. ACS patients have also undergone coronary angiography examination that verified the presence of significant stenosis. Controls were subjects age matched without MI/CAD history with negative coronary angiography findings (<30% stenosis), or negative stress test, or subjects without symptoms of disease that were admitted at the same hospitals as cases and were free of any cardiovascular disease, cancer, or inflammatory diseases. Subjects with renal or hepatic disease were excluded from both study groups. The bioethics committee of Harokopio University approved the study and all participants gave their informed consent[31].

ULSAM

The ULSAM (Uppsala Longitudinal Study of Adult Men) is a population-based cohort study of diabetes and cardiovascular disease in men. In 1970, all men born between 1920 and 1924 and residing in Uppsala, Sweden were invited to a health survey at age 50 years. In all, 2322 of 2841 invited men participated (82%). Participants were identified as having CHD if they had a hospital admission with CHD as the primary cause of hospitalization and/or died with CAD as the underlying cause. CHD was defined as acute myocardial infarction (ICD-8 and ICD-9 code 410, ICD-10 codes I21-I22) or unstable angina (ICD-8 code 411, ICD-9 code 411B, ICD-10 code I20.0). The positive predictive values (i.e. validity) of the CHD diagnosis in the Swedish hospital discharge register has been demonstrated to be at least 95% when only primary diagnoses are considered. Non-cases were selected from the same longitudinal, community-based cohort study[32].

WTCCC-CAD2

WTCCC-CAD2 cases comprise patients from four studies: (i) young MI cases with an event below the age of 50 years recruited into the Premature Acute Myocardial Infarction Study (PRAMIS) study[33] (n=214, 85.5% males, mean age at event 42.4±5.8 yrs); (ii) MI cases recruited into The Secondary Prevention of Acute Coronary Events – Reduction of Cholesterol to Key European Targets (SPACE ROCKET) Trial[34] (n=499, 84.0% males, mean age at event 57.7±8.4 years); (iii) MI cases recruited in the Outcomes from Percutaneous coronary intervention by Evaluation of Risk Attributes (OPERA) Trial (n=337, 75.5% males, mean age at event 55.8±8.4 years) and (iv) cases (n= 253, 73.5% males, mean age at event 49.6±7.6 years; 66% MI, 34% PTCA/CABG) from the British Heart Foundation Family Heart Study (BHF-FHS)[35] additional to those used in

Nature Genetics: doi:10.1038/ng.2480

the initial WTCCC Study. Case status in all studies was validated by direct review of clinical notes. All cases were of White European origin. Controls comprised subjects from the United Kingdom 1958 Birth Cohort (n=4246, 56.1% males, 44 yrs mean age)

Stage 3 Studies

Corogene

The aim of this study is to collect 5000 patients assigned for coronary angiogram in Helsinki University Central Hospital. A further aim is to record echocardiograms of the aortic valves in 3500 consecutive patients undergoing coronary angiography. All patients that give informed consent will be included into this prospective study. Peripheral blood leukocyte DNA will be collected, and the patients will be genotyped for a number of candidate genes related to inflammation, immunologic responses, and regulation of lipid and bone metabolisms. The echocardiographic changes of the aortic valve, stiffening, thickening, calcification and flow obstruction will be assessed[36].

FINCAVAS

The Finnish Cardiovascular Study (FINCAVAS) participant pool consists of patients who were recruited during 2001-2007 who underwent exercise stress tests at Tampere University Hospital, follow-up data was gathered at 2, 5 and 10 years. All the consecutive patients coming in for an exercise stress test and willing to participate in the study are recruited between with the aim to recruit roughly 5,000 patients. The study protocol was approved by the Ethical Committee of the Hospital District of Pirkanmaa, Finland, and all patients have given informed consent prior to the interview and measurements as stipulated in the Declaration of Helsinki. Cases are defined as >50% stenosis in one or more coronary arteries in coronary angiography, or strong Bayesian posterior probability for CAD after exercise test using a bicycle ergometer, or hospital verified myocardial infarction in medical history. Controls were defined as <50% stenosis in coronary arteries, or low Bayesian posterior probability for CAD after exercise test, and no myocardial infarction in medical history[37].

GenRIC

The Genomics Research in Cardiovascular disease (GenRIC) is an East Asian study were participants were recruited in Seoul, South Korea. Cases were selected from hospital admissions meeting the following phenotype criteria. Angiographic definition: significant reduction in luminal diameter due to coronary atheromatous disease (i.e. with stenosis greater than 50%). Clinical definitions: Stable angina: chest or arm discomfort that may not be described as pain but is reproducibly associated with physical exertion or stress and is relieved within 5-10 minutes by rest and/or sublingual nitroglycerin (Ref. Harrison's Principles of Internal Medicine; Longo et al), Unstable angina: Angina pectoris or equivalent ischemic discomfort with at least one of three features
1) it occurs at rest, usually resting > 10 minutes, 2) it is severe and of noew onset (≤4-6 weeks), 3) it occurs with a crescendo pattern (Ref. Harrison's Principles of Internal Medicine; Longo et al), Myocardial infarction: Spontaneous or secondary myocardial infarction according to the "universal definition of myocardial infarction"[38]. Controls are participants of a population based study that do not present the CAD phenotype and were matched to cases by sex and age.

Nature Genetics: doi:10.1038/ng.2480

2. Background information on novel coronary artery disease risk loci

IL6R

This gene encodes a subunit of the interleukin 6 (IL6) receptor complex. Interleukin 6 is a potent pleiotropic cytokine that regulates cell growth and differentiation and activation may lead to the regulation of the immune response, acute-phase reactions and hematopoiesis. The IL6 receptor is a protein complex consisting of this protein and interleukin 6 signal transducer (IL6ST/GP130/IL6-beta), a receptor subunit also shared by many other cytokines. A pseudogene of this gene is found on chromosome 9, alternatively spliced transcript variants encoding distinct isoforms have been reported and a short soluble form may also be released from the membrane by proteolysis. Low concentration of the soluble form acts as an agonist of IL6 activity. Dysregulated production of IL6 and this receptor are implicated in the pathogenesis of many diseases, such as multiple myeloma, autoimmune diseases and prostate cancer. Common variants in *IL6R,* effect allele rs4129267-T (r^2 of 0.542 with lead CAD SNP rs4845625 in **Table 2**), have been associated with asthma[39] and decrease in levels of C-reactive protein[40].

ABCG5 - ABCG8

The ABCG5 and ABCG8 genes are tandemly arrayed on chromosome 2, in a head-to-head orientation, the proteins are members of the superfamily of ATP-binding cassette (ABC) transporters. ABC proteins transport various molecules across extra- and intra-cellular membranes. The proteins form heterodimers that function as a transporter that appears to play a role in the selective transport of the dietary cholesterol in and out of the enterocytes and in the selective sterol excretion by the liver into bile. It is expressed in a tissue-specific manner in the liver, colon, and intestine.. Mutations in these genes may contribute to sterol accumulation and atheroschlerosis, and have been observed in patients with sitosterolemia. Sitosterolemia patients have hypercholesterolemia, very high levels of plant sterols in the plasma, and frequently develop tendon and tuberous xanthomas, accelerated atherosclerosis and premature coronary artery disease. In a meta-analysis of 16 population-based cohorts, ABCG5 has been associated to serum lipid levels (total cholesterol, P = 1.5×10^{-11}; LDL, P = 2.6×10^{-10})[41]. Common variants in ABCG8 and ABO (also a CAD risk locus) have been associated with serum phytosterol levels[42]. Effects in ABCG8 were independently related to SNPs rs4245791 (r^2 of 1 with the lead CAD SNP rs6544713) and rs41360247 r^2 of 0.047 with the lead CAD SNP rs6544713) which showed combined P-values of 1.6×10^{-50} and 6.2×10^{-25}, respectively (n=4412). Serum campesterol was elevated 12% for each rs4245791 T-allele also associated with 40% decreased hepatic ABCG8 mRNA expression[42] (P=0.009).

APOB

Apolipoprotein B (APOB) is the primary apolipoprotein of low-density lipoproteins (LDL) which is responsible for carrying cholesterol to tissues. The protein occurs in the plasma in 2 main isoforms, APOB48 and APOB100. The first is synthesized exclusively by the small intestine, the second by the liver, both isoforms are coded by a single mRNA transcript. APOB48 is generated when a stop codon (UAA) at residue 2153 is created by RNA editing, a tissue-specific splicing gene determines which isoform is ultimately produced. APOB48 lacks APOB100's C-terminal LDL receptor binding region. Apo B-100 functions as a recognition signal for the cellular binding and internalization of LDL particles by the apoB/E complex. High levels of APOB can lead to plaques that cause vascular disease (atherosclerosis), leading to heart disease. APOB100 levels are a better indicator of cardiovascular disease risk than total cholesterol or LDL. Hypobetalipoproteinemia is a genetic disorder that can be caused by a mutation in the APOB gene. Mutations in this gene or its regulatory region cause hypobetalipoproteinemia, normotriglyceridemic hypobetalipoproteinemia, and hypercholesterolemia due to ligand-defective apoB, diseases affecting plasma cholesterol and apoB levels. Mice overexpressing mApoB have increased levels of LDL and decreased levels of HDL Mice containing only one functional copy of the mApoB gene show the opposite effect, being resistant to hypercholesterolemia. Mice containing no functional copies of the gene are not

Nature Genetics: doi:10.1038/ng.2480

viable. APOB has been associated to serum lipid levels: rs1367117-A increases total cholesterol (4.16 [3.73-4.59] mg/dL) and LDL-cholesterol (4.05 [3.68-4.42] mg/dL) whereas rs1042034-C increases HDL-cholesterol (0.9 [0.72-1.08] mg/dL) and decreases triglycerides (5.99 [5.11-6.87] mg/dL)[43]. However, our lead CAD SNP rs515135 has low LD with the above two SNPs (r^2 of 0.135 with rs1367117 and 0.033 with 1042034). As reported in **Supplementary Table 5** rs515135 was strongly asociated for total and LDL chrolesterol in that same study.

ZEB2-AC074093.1

Zinc finger E-box-binding homeobox 2 (*ZEB2)* is a transcriptional inhibitor that binds to DNA sequence 5'-CACCT-3' in different promoters, represses transcription of E-cadherin. Mutations in the ZEB2 gene are associated with the Mowat-Wilson syndrome, a complex developmental disorder characterized by mental retardation, delayed motor development, epilepsy, microcephaly and a wide spectrum of clinically heterogeneous features suggestive of neurocristopathies at the cephalic, cardiac, and vagal levels.
Activin A type II receptor (ACVR2A). Activins are dimeric growth and differentiation factors which belong to the transforming growth factor-beta (TGF-beta) superfamily of structurally related signaling proteins. Activins signal through a heteromeric complex of receptor serine kinases. On ligand binding, forms a receptor complex consisting of two type II and two type I transmembrane serine/threonine kinases. Type II receptors phosphorylate and activate type I receptors which autophosphorylate, then bind and activate SMAD transcriptional regulators. The protein is a receptor for activin A, activin B and inhibin A

VAMP5 - VAMP8 - GGCX

Vesicle-associated membrane protein 8 (VAMP8) is involved in the targeting and/or fusion of transport vesicles to their target membrane. Involved for dense-granule secretion in platelets. Plays a role in regulated enzyme secretion in pancreatic acinar cells. Involved in the abscission of the midbody during cell division, which leads to completely separate daughter cells. Involved in the homotypic fusion of early and late endosomes.

Gamma-glutamyl carboxylase (GGCX) is an enzyme that catalyzes the posttranslational modification of vitamin K-dependent proteins. Many of these vitamin K-dependent proteins are involved in coagulation so the function of the encoded enzyme is essential for hemostasis. Most gla domain-containing proteins depend on this carboxylation reaction for posttranslational modification. Defects in GGCX are a cause of combined deficiency of vitamin K-dependent clotting factors type 1 (VKCFD1). In humans, the gamma-glutamyl carboxylase enzyme is most highly expressed in the liver

GUCY1A3

Guanylate cyclase soluble subunit alpha-3 is an enzyme that in humans is encoded by the *GUCY1A3* gene. Soluble guanylate cyclase (sGC), a heterodimeric protein consisting of an alpha and a beta subunit, catalyzes the conversion of guanosine-5'-triphosphate (GTP) into 3',5'-guanosine monophosphate (cGMP) and pyrophosphate and functions as the main receptor for nitric oxide. Nitric oxide affects IL-6, which is the ligand for IL6R (see above), expression in human peripheral blood mononuclear cells involving cGMP-dependent modulation of NF-κB activity[44]. Key physiological roles for guanylyl cyclases include regulation of cell hyperplasia, hypertrophy, migration, extracellular matrix production, cell differentiation and tumor progression. In addition, guanylyl cyclases mediate important communication between the heart, intestine and kidney to regulate blood volume and Na+ balance.

EDNRA

Nature Genetics: doi:10.1038/ng.2480

Endothelin receptor type A, is a human G protein-coupled receptor that has been shown to interact with HDAC7A and HTATIP. This family of receptors are located primarily in the vascular smooth muscle where they play a role in vasoconstriction and cell proliferation. This gene encodes the receptor for endothelin-1, a peptide that plays a role in potent and long-lasting vasoconstriction, Isoform 1, isoform 3 and isoform 4 are expressed in a variety of tissues, with highest levels in the aorta and cerebellum. This receptor associates with guanine-nucleotide-binding (G) proteins. Polymorphisms in this gene have been linked to migraine headache resistance. Alternative splicing results in multiple transcript variants. In a recent study rs1878406-T has been associated with a .0087 [0.01-0.01] per unit increase in carotid intima media thickness and .1993 [0.14-0.26] per unit increase in plaque[45].

SLC22A4 - SLC22A5

Solute carrier family 22, member 4 (SLC22A4), the encoded protein is an organic cation transporter and plasma integral membrane protein containing eleven putative transmembrane domains as well as a nucleotide-binding site motif, the protein is responsible for the cotransport of sodium ions and ergothioneine, which is an antioxidant, into cells. Widely expressed. Highly expressed in whole blood, bone marrow, trachea and fetal liver, highly expressed in intestinal cell types affected by Crohn disease, including epithelial cells. Genetic variations in SLC22A4 are a cause of susceptibility to Crohn‘s disease[46].

Solute carrier family 22, member 4 (SLC22A5), is a membrane transport protein associated with primary carnitine deficiency. Polyspecific organic cation transporters in the liver, kidney, intestine, and other organs are critical for elimination of many endogenous small organic cations as well as a wide array of drugs and environmental toxins. The encoded protein is involved in the active cellular uptake of carnitine. Mutations in this gene are the cause of systemic primary carnitine deficiency (CDSP), an autosomal recessive disorder manifested early in life by hypoketotic hypoglycemia and acute metabolic decompensation, and later in life by skeletal myopathy or cardiomyopathy. Strongly expressed in kidney, skeletal muscle, heart and placenta.

Both SLC22A4 and SLC22A5 are located in the same recombination interval with IL5 at 5q31.1. The 5q31.1 region has been associated with fibrinogen levels (rs2522056), eosonophil numbers (rs4143832; near *IL5*), C-reactive proteinlevels (rs4705952; near *IRF1*) and Crohn's disease (rs12521868; near *C5orf56*). We found no evidence of association with CAD risk for rs4143832, rs4705952 and rs12521868; rs2522056 was not tested in our study.

KCNK5

The potassium channel subfamily K member 5 (*KCNK5*) gene encodes $K_{2P}5.1$, one of the members of the superfamily of potassium channel proteins containing two pore-forming P domains. The gene is mainly expressed in the cortical distal tubules and collecting ducts of the kidney. The protein is highly sensitive to external pH and this, in combination with its expression pattern, suggests it may play an important role in renal potassium transport.

PLG

Plasmin is released as a zymogen called plasminogen (PLG) from the liver into the systemic circulation. Plasminogen is converted into active plasmin by a variety of enzymes, including tissue plasminogen activator (tPA), urokinase plasminogen activator (uPA), kallikrein, and factor XII (Hageman factor). Plasmin is a serine protease that acts to dissolve fibrin blood clots. The conversion of plasminogen to plasmin involves the cleavage of the peptide bond between Arg-560 and Val-561, two transcript variants encoding different isoforms have been found for this gene. Deficiency in plasmin may lead to thrombosis, as clots are not degraded adequately. Its role in tissue remodeling and tumor invasion may be modulated by CSPG4.

Nature Genetics: doi:10.1038/ng.2480

HDAC9

Histone acetylation/deacetylation alters chromosome structure and affects transcription factor access to DNA. The Histone deacetylase 9 gene has sequence homology to members of the histone deacetylase family, histone deacetylation gives a tag for epigenetic repression and plays an important role in transcriptional regulation, cell cycle progression and developmental events. Multiple alternatively spliced transcripts have been described for this gene. This encoded protein may play a role in hematopoiesis, inhibition of skeletal myogenesis, involvement in heart development and protection of neurons from apoptosis, both by inhibiting JUN phosphorylation by MAPK10 and by repressing JUN transcription via HDAC1 recruitment to JUN promoter. HDAC9 has been associated to large vessel ischemic stroke[47].

LPL

Lipoprotein lipase (LPL) is a water soluble enzyme that hydrolyzes triglycerides in lipoproteins, such as those found in chylomicrons and very low-density lipoproteins (VLDL), into two free fatty acids and one monoacylglycerol molecule. It is also involved in promoting the cellular uptake of chylomicron remnants, cholesterol-rich lipoproteins, and free fatty acids. LPL requires APOC2 acts as a coactivator of LPL activity in the presence of lipids on the luminal surface of vascular endothelium. LPL is attached to the luminal surface of endothelial cells in capillaries. It is most widely distributed in adipose, heart, and skeletal muscle tissue. Defects in LPL are the cause of lipoprotein lipase deficiency also known as familial chylomicronemia or hyperlipoproteinemia type I. Women with LPL deficiency have been reported to have a significantly higher risk of coronary artery disease ($P = 0.013$)[48]. The S447X variant of *LPL* has been inversely associated with severity of coronary artery disease suggesting a protective role[49].

TRIB1

Tribbles homolog 1 belongs to the protein kinase superfamilyand interacts with MAPK kinases and regulates activation of MAP kinases. Expressed in most human tissues with the highest levels in skeletal muscle, thyroid gland, pancreas, peripheral blood leukocytes, and bone marrow.

FURIN- FES

Furin is in the upstream region of the oncogene FES, the gene was known as FUR (FES Upstream Region) and therefore the protein was named furin. Furin is enriched in the Golgi apparatus, where it functions to cleave other proteins into their mature/active forms downstream of a basic amino acid target sequence (RX(K/R)R consensus motif). This gene is thought to play a role in tumor progression, expression of furin in T-cells is required for maintenance of peripheral immune tolerance. Furin is also utilized by a number of pathogens, the envelope proteins of HIV, influenza and dengue fever viruses must be cleaved by furin or furin-like proteases to become fully functional. Anthrax toxin, pseudomonas exotoxin, and papillomaviruses must be processed by furin during their initial entry into host cells.

The FES gene encodes the human cellular counterpart of a feline sarcoma retrovirus protein with transforming capabilities. The gene product has tyrosine-specific protein kinase activity and that activity is required for maintenance of cellular transformation. Its chromosomal location has linked it to a specific translocation event identified in patients with acute promyelocytic leukemia but it is also involved in normal hematopoiesis as well as growth factor and cytokine receptor signaling. Alternative splicing results in multiple variants encoding different isoforms. Can act as proto-oncogene in some types of cancer, possibly due to abnormal activation of the kinase. but as tumor suppressor in other types of cancer.

FLT1

Oncogene FLT belongs to the src gene family and is related to oncogene ROS, like other members of this family, it shows tyrosine protein kinase activity that is important for the control of cell proliferation and

Nature Genetics: doi:10.1038/ng.2480

differentiation. This gene encodes a member of the vascular endothelial growth factor receptor (VEGFR) family. VEGFR family members are receptor tyrosine kinases (RTKs) which contain an extracellular ligand-binding region with seven immunoglobulin (Ig)-like domains, a transmembrane segment, and a tyrosine kinase (TK) domain within the cytoplasmic domain. Multiple transcript variants encoding different isoforms have been found for this gene, isoforms include a full-length transmembrane receptor isoform and shortened, soluble isoforms. The soluble isoforms are associated with the onset of pre-eclampsia. This protein binds to VEGFR-A, VEGFR-B and placental growth factor and the VEGF-kinase ligand/receptor signaling system plays a key role in vascular development and regulation of vascular permeability. Isoform SFlt1 may have an inhibitory role in angiogenesis. Detected in normal lung, but also in placenta, liver, kidney, heart and brain tissues. Specifically expressed in most of the vascular endothelial cells, and also expressed in peripheral blood monocytes and placental trophoblast cells.

3. Network analysis in genes not associated to CAD

In a control experiment for the network analysis we tested the bottom (least significant) 1,885 SNPs of the FDR analysis. We assigned 369 genes to this SNP set based on physical proximity and subjected them to network analysis with the Ingenuity Pathway Analysis software under the same conditions used for the CAD set (FDR <10%). We note that SNP content on the Metabochip array is not random as it has been selected for association to a number of different traits. In addition, the Metabochip includes the NHGRI GWA catalogue of significantly associated SNPs (July 2009). Therefore, we expect some level of connectivity between genes when assessing the non CAD associated set.
IPA analysis generated ten networks and two overlapping networks (ON). One ON comprised networks1, 5-6) and the other networks 3 and 7. Connectivity in the CAD set was significantly higher (4 networks, one with 6 modules, one with two and two singletons). The genes in the seven networks are enriched for genes known to be involved in cell-to-cell signaling and interaction ($P = 1.32 \times 10^{-4}$), cellular growth and proliferation ($P= 1.71 \times 10^{-4}$) and carbohydrate metabolism ($P = 2.27 \times 10^{-4}$). No significant enrichment was observed for lipid metabolism (P= 2.27E-04) which we found in the CAD associated set ($P= 7.11 \times 10^{-10}$). Looking at enrichment under the Physiological System Development and Function terms the top one was behavior ($P = 8.39 \times 10^{-6}$) as opposed to tissue morphology (size and area of atherosclerotic lesion, quantity of leukocytes, macrophages and smooth muscle cells; $P = 9 \times 10^{-10}$) in the CAD set. No enrichment was found for immune cell trafficking (migration and adhesion) which reached $P = 9.38 \times 10^{-8}$ in the CAD set. Based on disease terms the molecules in the control set are enriched for Immunological disease (1.79 x 10E-13) as opposed to the CAD set which is as expected enriched for Cardiovascular Disease (7.89 x 10E-10).

Finally, when looking at the canonical pathways mapping to the seven networks the most significant were

**Name**	**p-value**	**Ratio**
Hepatic Cholestasis	2.79E-04 9/146	(0.062)
GNRH Signaling	5.92E-04 8/136	(0.059)
Molecular Mechanisms of Cancer	6.93E-04 14/367	(0.038)
Nicotinate and Nicotinamide Metabolism	8.5E-04 7/101	(0.069)
TGF-b Signaling	1.6E-03 6/89	(0.067)

In addition to an overall weaker representation of query molecules in the top five pathways compared to the CAD set (**Figure 2A** main text) the top two pathways, Hepatic Cholestasis and GNRH Signaling, which include 146 and 136 members respectively, do not contain any gene in the 47 CAD genome-wide significant loci (Table **1** and **2** main text).

Nature Genetics: doi:10.1038/ng.2480

4. Sources of Funding

P.D., I. B., and W.H.O. are supported by the Wellcome Trust Grant 098051 which also covered genotyping costs for THISEAS, PROMIS, AMC-PAS, EPIC-Norfolk, GLACIER, and MORGAM. **Ulm (EMIL)** Deutsche Forschungsgemeinschaft (GrK 1041); **FINRISK 2007/ DILGOM** DILGOM-study was supported by the Academy of Finland, grant # 118065. VS was supported by grants #139635 and 129494 from the Academy of Finland. S.R was supported by the Academy of Finland Center of Excellence in Complex Disease Genetics (213506 and 129680), Academy of Finland (251217), the Finnish foundation for Cardiovascular Research and the Sigrid Juselius Foundation; **Estonian Biobank** EGCUT received financing by FP7 grants (201413, 245536), also received targeted financing from Estonian Government SF0180142s08 and from the European Union through the European Regional Development Fund, in the frame of Centre of Excellence in Genomics and Estonian Research Infrastructure's Roadmap; **SHEEP-SCARF** European Commission (LSHM-CT- 2007-037273), the Swedish Heart-Lung Foundation, the Swedish Research Council (8691), the Knut and Alice Wallenberg Foundation, the Foundation for Strategic Research, the Torsten and Ragnar Söderberg Foundation, the Strategic Cardiovascular Programme of Karolinska Institutet and the Stockholm County Council and the Stockholm County Council (560183); **ASAP-eQTL** Swedish Research Council (12660), the Swedish Heart-Lung foundation, the European Commission (FAD, Health-F2-2008-200647) and a donation by Fredrik Lundberg; **PennCATH/MedStar** was supported by the Cardiovascular Research Institute, Washington Hospital Center and by a research grant from GlaxoSmithKline; **METSIM** was supported by the Academy of Finland (M.L.), the Finnish Diabetes Research Foundation (M.L.), the Finnish Cardiovascular Research Foundation (M.L., J.K.), an EVO grant from the Kuopio University Hospital (5263) and the National Institutes of Health NIDDK (DK062370); **GerMIFS (1, 2 & 3) and Angio-Lüb/KORA** were supported by the Deutsche Forschungsgemeinschaft and the German Federal Ministry of Education and Research (BMBF) in the context of the German National Genome Research Network (NGFN-2 and NGFN-plus), the FP6 and FP7 EU funded integrated projects Cardiogenics (LSHM-CT-2006-037593), ENGAGE (201413), the bi-national BMBF/ANR funded project CARDomics (01KU0908A), and the Nordic Center of Cardiovascular Research (NCCR); **GerMIFS 3** also by the Munich Heart Alliance (MHA); **Corogene** the Aarno Koskelo Foundation, the Finnish Foundation for Cardiovascular Research, and the EVO funds of Helsinki University Central Hospital; **ADVANCE** was supported by a grant from the Reynold's Foundation and NHLBI grant HL087647; **Decode** the CAD/MI Study was funded by NIH grant, National Heart, Lung and Blood Institute R01HL089650-02 and in part through the European Community's Seventh Framework Programme (FP7/2007-2013), ENGAGE project, grant agreement HEALTH-F4-2007- 201413; **FRISCII** was supported by the Swedish Research Council and the Swedish Heart and Lung Foundation; **GLACIER** The GLACIER Study and part of PWF's and DS' salaries were funded by grants from the Swedish Research Council, Swedish Heart-Lung Foundation, Novo Nordisk, Umeå Medical Research Foundation, and the Swedish Diabetes Association (to PWF). I.B. was supported by the United Kingdom NIHR Cambridge Biomedical Research Centre. Genotyping for this specific project was funded by the Wellcome Trust Sanger Institute**; HPS** was supported for genotyping by a grant to Oxford University and CNG from Merck & Co. Jemma C Hopewell acknowledges support from the BHF Centre of Research Excellence, Oxford; **LOLIPOP** supported by the National Institute for Health Research Comprehensive Biomedical Research Centre at Imperial College Healthcare NHS Trust, the British Heart Foundation (SP/04/002), the Medical Research Council (G0700931), the Wellcome Trust (084723/Z/08/Z), the National Institute for Health Research (RP-PG-0407-10371) and the US National Institute of Diabetes and Digestive and Kidney Diseases (U01-DK085545); **MORGAM** was part funded through the European Community's Sixth Framework Programme Cardiogenics project, grant agreement LSHM-CT-2006-037593 and Seventh Framework Programme ENGAGE project, grant agreement HEALTH-F4-2007-201413. The ATBC Study was supported by US Public Health Service contracts N01-CN-45165, N01-RC-45035 and N01-RC-37004 from the National Cancer Institute. The Italian study was supported by the Health Administration of Regione Lombardia [grant numbers 9783/1986, 41795/1993, 31737/1997 and 17155/2004], for the baseline examinations and the follow-up; **Ottawa Heart Genomics Study** supported by Canadian Institutes of Health Research (CIHR) #MOP82810 (to Dr. Roberts), Canada Foundation for Innovation (CFI) # 11966 (to

Dr. Roberts), The Heart and Stroke Foundation of Ontario #NA6001 (to Dr. McPherson), CIHR #MOP172605 (to Dr. McPherson), and CIHR #MOP77682 (to Dr. Stewart); **PROCARDIS** was supported by the EU FP7 Program (LSHM-CT- 2007-037273), AstraZeneca, the British Heart Foundation, the Oxford BHF Centre of Research Excellence, the Wellcome Trust (075491/Z/04), the Swedish Research Council, the Knut and Alice Wallenberg Foundation, the Swedish Heart-Lung Foundation, the Torsten and Ragnar Söderberg Foundation, the Strategic Cardiovascular Program of Karolinska Institutet and Stockholm County Council, the Foundation for Strategic Research and the Stockholm County Council (560283); **SHEEP-SCARF** supported by The Swedish Heart-Lung Foundation, the Swedish Research Council, the Strategic Cardiovascular Programme of Karolinska Institutet and the Stockholm County Council, the Strategic support for epidemiological research at Karolinska Institutet and the Stockholm County Council; **WTCCC** The WTCCC and WTCCC-CAD2 studies were supported by the Wellcome Trust, the British Heart Foundation, the Medical Research Council, and the UK National Institute for Health Research**.** NJS holds a chair funded by the British Heart Foundation and is an NIHR Senior Investigator. This work forms part of the portfolio of research supported by the NIHR Leicester Cardiovascular Biomedical Research Unit. Analysis of the data was supported by the European Union Framework 7 ENGAGE Project (HEALTH-F4-2007- 201413); **Korean GenRIC** was supported by grants from the Korean Genome Analysis Project (4845-301), the Korea Biobank Project (4851-307), the Korean Genome Epidemiology Study (4851-302) and the Korea Healthcare technology R&D project (A000385) by the Ministry for Health, Welfare and Family Affairs, the Korean government for the National Research Foundation (MEST) (2010-0020258), and the Korea National Institute of Health, Korea Center for Disease Control, Republic of Korea. **PIVUS/Swedish Twin Registry** was supported by grants from the US National Institutes of Health (AG028555, AG08724, AG04563, AG10175, AG08861), the Swedish Research Council, the Swedish Heart-Lung Foundation, the Swedish Foundation for Strategic Research, the Royal Swedish Academy of Science, and ENGAGE (FP7, HEALTH-F4-2007-201413); The SNP Technology Platform is supported by Uppsala University, Uppsala University Hospital and the Swedish Research Council for Infrastructures. **FINCAVAS** was supported by the Competitive Research Funding of the Tampere University Hospital (Grant 9M048 and 9N035), the Finnish Cultural Foundation, the Finnish Foundation for Cardiovascular Research, the Emil Aaltonen Foundation, Finland, and the Tampere Tuberculosis Foundation; **Go-DARTS Dundee** The Wellcome Trust supported the Wellcome Trust UK Type 2 Diabetes Case Control Collection (Go-DARTS) and the Scottish Health Informatics Programme. The Chief Scientist Office supported informatics. Project funded by the UK Medical Research Council (G0601261); **FGENTCARD** Wellcome Senior Fellowship (057733) and European Commission FP6 grant FGENTCARD (LSHG-CT-2006-037683) and Wellcome Trust Grant 075491/Z/04; **EPIC-Norfolk study** was supported by the Medical Research Council UK and Cancer Research UK.

5. Consortia

Membership of the Cardiogenics Consortium

Tony Attwood[1], Stephanie Belz[2], Peter Braund[3], Jessy Brocheton[4], François Cambien[4], Jason Cooper[5], Abi Crisp-Hihn[1], Patrick Diemert (formerly Linsel-Nitschke)[2], Panos Deloukas[6], Jeanette Eardman[2], Nicola Foad[1], Tiphaine Godefroy[4], Alison H Goodall[3,11], Jay Gracey[3], Emma Gray[6],, Rhian Gwilliams[6], Susanne Heimerl[7], Christian Hengstenberg[7], Jennifer Jolley[1], Unni Krishnan[3], Heather Lloyd-Jones[1], Ulrika Liljedahl[8], Ingrid Lugauer[7], Per Lundmark[8], Seraya Maouche[2,4], Jasbir S Moore[3] , Gilles Montalescot[4], David Muir[1], Elizabeth Murray[1], Chris P Nelson[3], Jessica Neudert[9], David Niblett[6], Karen O'Leary[1], Willem H Ouwehand[1,6], Helen Pollard[3], Carole Proust[4], Angela Rankin[1], Augusto Rendon[12], Catherine M Rice[6], Hendrik B Sager[2], Nilesh J Samani[3,11], Jennifer Sambrook[1], Gerd Schmitz[10], Michael Scholz[9], Laura Schroeder[2], Heribert Schunkert[2], Jonathan Stephens[1], Ann-Christine Syvannen[8], Stefanie Tennstedt (formerly Gulde)[2], Chris Wallace[5].
[1]Department of Haematology, University of Cambridge, Long Road, Cambridge, CB2 2PT, UK and National Health Service Blood and Transplant, Cambridge Centre, Long Road, Cambridge, CB2 2PT, UK; [2]Medizinische Klinik 2, Universität zu Lübeck, Lübeck

Nature Genetics: doi:10.1038/ng.2480

Germany; [3]Department of Cardiovascular Sciences, University of Leicester, Glenfield Hospital, Groby Road, Leicester, LE3 9QP, UK; [4] INSERM UMRS 937, Pierre and Marie Curie University (UPMC, Paris 6) and Medical School, 91 Bd de l'Hôpital 75013, Paris, France; [5]Juvenile Diabetes Research Foundation/Wellcome Trust Diabetes and Inflammation Laboratory, Department of Medical Genetics, Cambridge Institute for Medical Research, University of Cambridge, Wellcome Trust/MRC Building, Cambridge, CB2 0XY, UK; [6]The Wellcome Trust Sanger Institute, Wellcome Trust Genome Campus, Hinxton, Cambridge CB10 1SA, UK; [7]Klinik und Poliklinik für Innere Medizin II, Universität Regensburg, Germany; [8]Molecular Medicine, Department of Medical Sciences, Uppsala University, Uppsala, Sweden; [9]Trium, Analysis Online GmbH, Hohenlindenerstr. 1, 81677, München, Germany; [10]Institut für Klinische Chemie und Laboratoriumsmedizin, Universität, Regensburg, D-93053 Regensburg, Germany; [11] Leicester NIHR Biomedical Research Unit in Cardiovascular Disease, Glenfield Hospital, Leicester, LE3 9QP, UK; [12] European Bioinformatics Institute, Wellcome Trust Genome Campus, Hinxton, Cambridge, CB10 1SD, UK

Members of the DIAGRAM Consortium

Benjamin F Voight[1,2,3], Laura J Scott[4], Valgerdur Steinthorsdottir[5], Andrew P Morris[6], Christian Dina[7,8], Ryan P Welch[9], Eleftheria Zeggini[6,10], Cornelia Huth[11,12], Yurii S Aulchenko[13], Gudmar Thorleifsson[5], Laura J McCulloch[14], Teresa Ferreira[6], Harald Grallert[11,12], Najaf Amin[13], Guanming Wu[15], Cristen J Willer[4], Soumya Raychaudhuri[1,2,16], Steve A McCarroll[1,17], Claudia Langenberg[18], Oliver M Hofmann[19], Josée Dupuis[20,21], Lu Qi[22-24], Ayellet V Segrè[1,2,17], Mandy van Hoek[25], Pau Navarro[26], Kristin Ardlie[1], Beverley Balkau[27,28], Rafn Benediktsson[29,30], Amanda J Bennett[14], Roza Blagieva[31], Eric Boerwinkle[32], Lori L Bonnycastle[33], Kristina Bengtsson Boström[34], Bert Bravenboer[35], Suzannah Bumpstead[10], Noël P Burtt[1], Guillaume Charpentier[36], Peter S Chines[33], Marilyn Cornelis[24], David J Couper[37], Gabe Crawford[1], Alex SF Doney[38,39], Katherine S Elliott[6], Amanda L Elliott[1,17,40], Michael R Erdos[33], Caroline S Fox[21,41], Christopher S Franklin[42], Martha Ganser[4], Christian Gieger[11], Niels Grarup[43], Todd Green[1,2], Simon Griffin[18], Christopher J Groves[14], Candace Guiducci[1], Samy Hadjadj[44], Neelam Hassanali[14], Christian Herder[45], Bo Isomaa[46,47], Anne U Jackson[4], Paul RV Johnson[48], Torben Jørgensen[49,50], Wen HL Kao[51,52], Norman Klopp[11], Augustine Kong[5], Peter Kraft[22,23], Johanna Kuusisto[53], Torsten Lauritzen[54], Man Li[51], Aloysius Lieverse[55], Cecilia M Lindgren[6], Valeriya Lyssenko[56], Michel Marre[57,58], Thomas Meitinger[59,60], Kristian Midthjell[61], Mario A Morken[33], Narisu Narisu[33], Peter Nilsson[56], Katharine R Owen[14], Felicity Payne[10], John RB Perry[62,63], Ann-Kristin Petersen[11], Carl Platou[61], Christine Proença[7], Inga Prokopenko[6,14], Wolfgang Rathmann[64], N William Rayner[6,14], Neil R Robertson[6,14], Ghislain Rocheleau[65-67], Michael Roden[45,68], Michael J Sampson[69], Richa Saxena[1,2,40], Beverley M Shields[62,63], Peter Shrader[3,70], Gunnar Sigurdsson[29,30], Thomas Sparsø[43], Klaus Strassburger[64], Heather M Stringham[4], Qi Sun[22,23], Amy J Swift[33], Barbara Thorand[11], Jean Tichet[71], Tiinamaija Tuomi[46,72], Rob M van Dam[24], Timon W van Haeften[73], Thijs van Herpt[25,55], Jana V van Vliet-Ostaptchouk[74], G Bragi Walters[5], Michael N Weedon[62,63], Cisca Wijmenga[75], Jacqueline Witteman[13], Richard N Bergman[76], Stephane Cauchi[7], Francis S Collins[77], Anna L Gloyn[14], Ulf Gyllensten[78], Torben Hansen[43,79], Winston A Hide[19], Graham A Hitman[80], Albert Hofman[13], David J Hunter[22,23], Kristian Hveem[61,81], Markku Laakso[53], Karen L Mohlke[82], Andrew D Morris[38,39], Colin NA Palmer[38,39], Peter P Pramstaller[83], Igor Rudan[42,84,85], Eric Sijbrands[25], Lincoln D Stein[15], Jaakko Tuomilehto[86], Andre Uitterlinden[25], Mark Walker[87], Nicholas J Wareham[18], Richard M Watanabe[76,88], Goncalo R Abecasis[4], Bernhard O Boehm[31], Harry Campbell[42], Mark J Daly[1,2], Andrew T Hattersley[62,63], Frank B Hu[22-24], James B Meigs[3,70], James S Pankow[89], Oluf Pedersen[43,90,91], H.-Erich Wichmann[11,12,92], Inês Barroso[10], Jose C Florez[1,2,3,93], Timothy M Frayling[62,63], Leif Groop[56,72], Rob Sladek[65-67], Unnur Thorsteinsdottir[5,94], James F Wilson[42], Thomas Illig[11], Philippe Froguel[7,95], Cornelia M van Duijn[13], Kari Stefansson[5,94], David Altshuler[1,2,3,17,40,93], Michael Boehnke[4], Mark I McCarthy[6,14,96].

[1]Broad Institute of Harvard and Massachusetts Institute of Technology (MIT), Cambridge, Massachusetts 02142, USA; [2]Center for Human Genetic Research, Massachusetts General Hospital, 185 Cambridge Street, Boston, Massachusetts 02114, USA; [3]Department of Medicine, Harvard Medical School, Boston, Massachusetts 02115, USA; [4]Department of Biostatistics, University of Michigan, Ann Arbor, Michigan 48109-2029, USA; [5]deCODE Genetics, 101 Reykjavik, Iceland; [6]Wellcome Trust Centre for Human Genetics, University of Oxford, Oxford, OX3 7BN, UK; [7]CNRS-UMR-8090, Institute of Biology and Lille 2 University, Pasteur Institute, F-59019 Lille, France; [8]INSERM UMR915 CNRS ERL3147 F-44007 Nantes, France; [9]Bioinformatics Program, University of Michigan, Ann Arbor MI USA 48109[10]Wellcome Trust Sanger Institute, Hinxton, CB10 1HH, UK; [11]Institute of Epidemiology, Helmholtz Zentrum Muenchen, 85764 Neuherberg, Germany; [12]Institute of Medical Informatics, Biometry and Epidemiology, Ludwig-Maximilians-Universität, 81377 Munich, Germany; [13]Department of Epidemiology, Erasmus University Medical Center,

Nature Genetics: doi:10.1038/ng.2480

P.O. Box 2040, 3000 CA Rotterdam, The Netherlands.; [14]Oxford Centre for Diabetes, Endocrinology and Metabolism, University of Oxford, OX3 7LJ, UK; [15]Ontario Institute for Cancer Research, 101 College Street, Suite 800, Toronto, Ontario M5G 0A3, Canada; [16]Division of Rheumatology, Immunology and Allergy, Brigham and Women's Hospital, Harvard Medical School, Boston, Massachusetts 02115, USA; [17]Department of Molecular Biology, Harvard Medical School, Boston, Massachusetts 02115, USA; [18]MRC Epidemiology Unit, Institute of Metabolic Science, Addenbrooke's Hospital, Cambridge CB2 0QQ, UK; [19]Department of Biostatistics, Harvard School of Public Health, Boston, Massachusetts 02115, USA; [20]Department of Biostatistics, Boston University School of Public Health, Boston, Massachusetts 02118, USA; [21]National Heart, Lung, and Blood Institute's Framingham Heart Study, Framingham, Massachusetts 01702, USA; [22]Department of Nutrition, Harvard School of Public Health, 665 Huntington Ave, Boston, MA 02115, USA; [23]Department of Epidemiology, Harvard School of Public Health, 665 Huntington Ave, Boston, MA 02115, USA; [24]Channing Laboratory, Dept. of Medicine, Brigham and Women's Hospital and Harvard Medical School, 181 Longwood Ave, Boston, MA 02115, USA; [25]Department of Internal Medicine, Erasmus University Medical Centre, PO-Box 2040, 3000 CA Rotterdam, The Netherlands; [26]MRC Human Genetics Unit, Institute of Genetics and Molecular Medicine, Western General Hospital, Edinburgh, EH4 2XU, UK; [27]INSERM U780, F-94807 Villejuif. France ; [28]University Paris-Sud, F-91405 Orsay, France; [29]Landspitali University Hospital, 101 Reykjavik, Iceland; [30]Icelandic Heart Association, 201 Kopavogur, Iceland; [31]Division of Endocrinology, Diabetes and Metabolism, Ulm University, 89081 Ulm, Germany; [32]The Human Genetics Center and Institute of Molecular Medicine, University of Texas Health Science Center, Houston, Texas 77030, USA; [33]National Human Genome Research Institute, National Institute of Health, Bethesda, Maryland 20892, USA; [34]R&D Centre, Skaraborg Primary Care, 541 30 Skövde, Sweden; [35]Department of Internal Medicine, Catharina Hospital, PO-Box 1350, 5602 ZA Eindhoven, The Netherlands; [36]Endocrinology-Diabetology Unit, Corbeil-Essonnes Hospital, F-91100 Corbeil-Essonnes, France ; [37]Department of Biostatistics and Collaborative Studies Coordinating Center, University of North Carolina at Chapel Hill, Chapel Hill, North Carolina, 27599, USA; [38]Diabetes Research Centre, Biomedical Research Institute, University of Dundee, Ninewells Hospital, Dundee DD1 9SY, UK; [39]Pharmacogenomics Centre, Biomedical Research Institute, University of Dundee, Ninewells Hospital, Dundee DD1 9SY, UK; [40]Department of Genetics, Harvard Medical School, Boston, Massachusetts 02115, USA; [41]Division of Endocrinology, Diabetes, and Hypertension, Brigham and Women's Hospital, Harvard Medical School, Boston, Massachusetts 02115, USA; [42]Centre for Population Health Sciences, University of Edinburgh, Teviot Place, Edinburgh, EH8 9AG, UK; [43]Hagedorn Research Institute, DK-2820 Gentofte, Denmark; [44]Centre Hospitalier Universitaire de Poitiers, Endocrinologie Diabetologie, CIC INSERM 0801, INSERM U927, Université de Poitiers, UFR, Médecine Pharmacie, 86021 Poitiers Cedex, France; [45]Institute for Clinical Diabetology, German Diabetes Center, Leibniz Center for Diabetes Research at Heinrich Heine University Düsseldorf, 40225 Düsseldorf, Germany; [46]Folkhälsan Research Center, FIN-00014 Helsinki, Finland; [47]Malmska Municipal Health Center and Hospital, 68601 Jakobstad, Finland; [48]Diabetes Research and Wellness Foundation Human Islet Isolation Facility and Oxford Islet Transplant Programme, University of Oxford, Old Road, Headington, Oxford, OX3 7LJ, UK; [49]Research Centre for Prevention and Health, Glostrup University Hospital, DK-2600 Glostrup, Denmark; [50]Faculty of Health Science, University of Copenhagen, 2200 Copenhagen, Denmark; [51]Department of Epidemiology, Johns Hopkins University, Baltimore, Maryland 21287, USA; [52]Department of Medicine, and Welch Center for Prevention, Epidemiology, and Clinical Research, Johns Hopkins University, Baltimore, Maryland 21287, USA; [53]Department of Medicine, University of Kuopio and Kuopio University Hospital, FIN-70211 Kuopio, Finland; [54]Department of General Medical Practice, University of Aarhus, DK-8000 Aarhus, Denmark; [55]Department of Internal Medicine, Maxima MC, PO-Box 90052, 5600 PD Eindhoven, The Netherlands; [56]Department of Clinical Sciences, Diabetes and Endocrinology Research Unit, University Hospital Malmö, Lund University, 205 02 Malmö, Sweden; [57]Department of Endocrinology, Diabetology and Nutrition, Bichat-Claude Bernard University Hospital, Assistance Publique des Hôpitaux de Paris, 75870 Paris Cedex 18, France; [58]INSERM U695, Université Paris 7, 75018 Paris , France; [59]Institute of Human Genetics, Helmholtz Zentrum Muenchen, 85764 Neuherberg, Germany; [60]Institute of Human Genetics, Klinikum rechts der Isar, Technische Universität München, 81675 Muenchen, Germany; [61]Nord-Trøndelag Health Study (HUNT) Research Center, Department of Community Medicine and General Practice, Norwegian University of Science and Technology, NO-7491 Trondheim, Norway; [62]Genetics of Complex Traits, Institute of Biomedical and Clinical Science, Peninsula Medical School, University of Exeter, Magdalen Road, Exeter EX1 2LU, UK; [63]Diabetes Genetics, Institute of Biomedical and Clinical Science, Peninsula Medical School, University of Exeter, Barrack Road, Exeter EX2 5DW, UK; [64]Institute of Biometrics and Epidemiology, German Diabetes Center, Leibniz Center for Diabetes Research at Heinrich Heine University Düsseldorf, 40225 Düsseldorf, Germany; [65]Department of Human Genetics, McGill University, Montreal H3H 1P3, Canada; [66]Department of Medicine, Faculty of Medicine, McGill University, Montreal, H3A 1A4, Canada; [67]McGill University and Genome Quebec Innovation Centre, Montreal, H3A 1A4. Canada; [68]Department of Metabolic Diseases, Heinrich Heine University Düsseldorf, 40225 Düsseldorf, Germany; [69]Department of Endocrinology and Diabetes, Norfolk and Norwich University Hospital NHS Trust , Norwich, NR1 7UY, UK.; [70]General Medicine Division, Massachusetts General Hospital, Boston, Massachusetts, USA; [71]Institut interrégional pour la Santé (IRSA), F-37521 La Riche, France; [72]Department of Medicine, Helsinki University Hospital, University of Helsinki, FIN-00290 Helsinki, Finland; [73]Department of Internal Medicine, University Medical Center Utrecht, 3584 CG Utrecht,The Netherlands; [74]Molecular Genetics, Medical Biology Section, Department of Pathology and Medical Biology, University Medical Center Groningen and University of Groningen, 9700 RB Groningen, The Netherlands ; [75]Department of Genetics, University Medical Center Groningen and University of Groningen, 9713 EX Groningen, The Netherlands; [76]Department of Physiology and Biophysics, University of Southern California School of Medicine, Los Angeles, California 90033, USA; [77]National Institute of Health, Bethesda, Maryland 20892, USA; [78]Department of Genetics and Pathology, Rudbeck Laboratory, Uppsala University, S-751 85 Uppsala, Sweden.; [79]University of Southern Denmark, DK-5230 Odense, Denmark; [80]Centre for Diabetes, Barts and The London School of Medicine and Dentistry, Queen Mary University of London, London E1 2AT, UK; [81]Department of

Nature Genetics: doi:10.1038/ng.2480

Medicine, The Hospital of Levanger, N-7600 Levanger, Norway; [82]Department of Genetics, University of North Carolina, Chapel Hill, North Carolina 27599, USA; [83]Institute of Genetic Medicine, European Academy Bozen/Bolzano (EURAC), Viale Druso 1, 39100 Bolzano, Italy; [84]Croatian Centre for Global Health, Faculty of Medicine, University of Split, Soltanska 2, 21000 Split, Croatia; [85]Institute for Clinical Medical Research, University Hospital "Sestre Milosrdnice", Vinogradska 29, 10000 Zagreb, Croatia; [86]Department of Chronic Disease Prevention, National Institute for Health and Welfare, Helsinki FIN-00300, Finland, ; [87]Diabetes Research Group, Institute of Cellular Medicine, Newcastle University, Framlington Place, Newcastle upon Tyne NE2 4HH, UK; [88]Department of Preventitive Medicine, Keck Medical School, University of Southern California, Los Angeles, CA, 90089-9001, USA; [89]Division of Epidemiology and Community Health, University of Minnesota, Minneapolis, Minnesota 55454, USA; [90]Department of Biomedical Science, Panum, Faculty of Health Science, University of Copenhagen, 2200 Copenhagen, Denmark; [91]Faculty of Health Science, University of Aarhus, DK–8000 Aarhus, Denmark; [92]Klinikum Grosshadern, 81377 Munich, Germany; [93]Diabetes Unit, Massachusetts General Hospital, Boston, Massachusetts 02144, USA; [94]Faculty of Medicine, University of Iceland, 101 Reykjavík, Iceland; [95]Genomic Medicine, Imperial College London, Hammersmith Hospital, W12 0NN, London, UK; [96]Oxford National Institute for Health Research Biomedical Research Centre, Churchill Hospital, Old Road Headington, Oxford, OX3 7LJ, UK, Old Road Headington, Oxford, OX3 7LJ, UK;

Members of the MuTHER Consortium

Kourosh R. Ahmadi[1], Chrysanthi Ainali[2], Amy Barrett[3], Veronique Bataille[1], Jordana T. Bell[1,4], Alfonso Buil[5], Panos Deloukas[6], Emmanouil T. Dermitzakis[5], Antigone S. Dimas[4,5], Richard Durbin[6], Daniel Glass[1], Elin Grundberg[1,6], Neelam Hassanali[3], Åsa K. Hedman[4], Catherine Ingle[6], David Knowles[7], Maria Krestyaninova[8], Cecilia M. Lindgren[4], Christopher E. Lowe[9,10], Mark I. McCarthy[3,4,11], Eshwar Meduri [1,6], Paola di Meglio[12], Josine L. Min[4], Stephen B. Montgomery[5], Frank O. Nestle[12], Alexandra C. Nica[5], James Nisbet[6], Stephen O'Rahilly[9,10], Leopold Parts[6], Simon Potter[6], Magdalena Sekowska[6], So-Youn Shin[6], Kerrin S. Small[1,6], Nicole Soranzo[1,6], Tim D. Spector[1], Gabriela Surdulescu[1], Mary E. Travers[3], Loukia Tsaprouni[6], Sophia Tsoka[2], Alicja Wilk[6], Tsun-Po Yang[6], Krina T. Zondervan[4]

[1]Department of Twin Research and Genetic Epidemiology, King's College London, London, UK; [2] Department of Informatics, School of Natural and Mathematical Sciences, King's College London, Strand, London, UK; [3] Oxford Centre for Diabetes, Endocrinology & Metabolism, University of Oxford, Churchill Hospital, Oxford, UK; [4] Wellcome Trust Centre for Human Genetics, University of Oxford, Oxford, UK; [5]Department of Genetic Medicine and Development, University of Geneva Medical School, Geneva, Switzerland; [6] Wellcome Trust Sanger Institute, Wellcome Trust Genome Campus, Hinxton, UK; [7] University of Cambridge, Cambridge, UK; [8] European Bioinformatics Institute, Hinxton, UK; [9] University of Cambridge Metabolic Research Labs, Institute of Metabolic Science Addenbrooke's Hospital Cambridge, UK; [10] Cambridge NIHR Biomedical Research Centre, Addenbrooke's Hospital, Cambridge, UK; [11] Oxford NIHR Biomedical Research Centre, Churchill Hospital, Oxford, UK; [12] St. John's Institute of Dermatology, King's College London, London, UK

Membership of the Wellcome Trust Case Control Consortium (WTCCC+)

Jan Aerts[1], Tariq Ahmad[2], Hazel Arbury[1], Anthony Attwood[1,3,4], Adam Auton[5], Stephen G Ball[6], Anthony J Balmforth[6], Chris Barnes[1], Jeffrey C Barrett[1], Inês Barroso[1], Anne Barton[7], Amanda J Bennett[8], Sanjeev Bhaskar[1], Katarzyna Blaszczyk[9], John Bowes[7], Oliver J Brand[8,10], Peter S Braund[11], Francesca Bredin[12], Gerome Breen[13,14], Morris J Brown[15], Ian N Bruce[7], Jaswinder Bull[16], Oliver S Burren[17], John Burton[1], Jake Byrnes[18], Sian Caesar[19], Niall Cardin[5], Chris M Clee[1], Alison J Coffey[1], John MC Connell[20], Donald F Conrad[1], Jason D Cooper[17], Anna F Dominiczak[20], Kate Downes[17], Hazel E Drummond[21], Darshna Dudakia[16], Andrew Dunham[1], Bernadette Ebbs[16], Diana Eccles[22], Sarah Edkins[1], Cathryn Edwards[23], Anna Elliot[16], Paul Emery[24], David M Evans[25], Gareth Evans[26], Steve Eyre[7], Anne Farmer[14], I Nicol Ferrier[27], Edward Flynn[7], Alistair Forbes[28], Liz Forty[29], Jayne A Franklyn[10,30], Timothy M Frayling[2], Rachel M Freathy[2], Eleni Giannoulatou[5], Polly Gibbs[16], Paul Gilbert[7], Katherine Gordon-Smith[19,29], Emma Gray[1], Elaine Green[29], Chris J Groves[8], Detelina Grozeva[29], Rhian Gwilliam[1], Anita Hall[16], Naomi Hammond[1], Matt Hardy[17], Pile Harrison[31], Neelam Hassanali[8], Husam Hebaishi[1], Sarah Hines[16], Anne Hinks[7], Graham A Hitman[32], Lynne Hocking[33], Chris Holmes[5], Eleanor Howard[1], Philip Howard[34], Joanna MM Howson[17], Debbie Hughes[16], Sarah Hunt[1], John D Isaacs[35], Mahim Jain[18], Derek P Jewell[36], Toby Johnson[34], Jennifer D Jolley[3,4], Ian R Jones[29], Lisa A Jones[19], George Kirov[29],

Nature Genetics: doi:10.1038/ng.2480

Cordelia F Langford[1], Hana Lango-Allen[2], G Mark Lathrop[37], James Lee[12], Kate L Lee[34], Charlie Lees[21], Kevin Lewis[1], Cecilia M Lindgren[8,18], Meeta Maisuria-Armer[17], Julian Maller[18], John Mansfield[38], Jonathan L Marchini[5], Paul Martin[7], Dunecan CO Massey[12], Wendy L McArdle[39], Peter McGuffin[14], Kirsten E McLay[1], Gil McVean[5,18], Alex Mentzer[40], Michael L Mimmack[1], Ann E Morgan[41], Andrew P Morris[18], Craig Mowat[42], Patricia B Munroe[34], Simon Myers[18], William Newman[26], Elaine R Nimmo[21], Michael C O'Donovan[29], Abiodun Onipinla[34], Nigel R Ovington[17], Michael J Owen[29], Kimmo Palin[1], Aarno Palotie[1], Kirstie Parnell[2], Richard Pearson[8], David Pernet[16], John RB Perry[2,18], Anne Phillips[42], Vincent Plagnol[17], Natalie J Prescott[9], Inga Prokopenko[8,18], Michael A Quail[1], Suzanne Rafelt[11], Nigel W Rayner[8,18], David M Reid[33], Anthony Renwick[16], Susan M Ring[39], Neil Robertson[8,18], Samuel Robson[1], Ellie Russell[29], David St Clair[13], Jennifer G Sambrook[3,4], Jeremy D Sanderson[40], Stephen J Sawcer[43], Helen Schuilenburg[17], Carol E Scott[1], Richard Scott[16], Sheila Seal[16], Sue Shaw-Hawkins[34], Beverley M Shields[2], Matthew J Simmonds[8,10], Debbie J Smyth[17], Elilan Somaskantharajah[1], Katarina Spanova[16], Sophia Steer[44], Jonathan Stephens[3,4], Helen E Stevens[17], Kathy Stirrups[1], Millicent A Stone[45,46], David P Strachan[47], Zhan Su[5], Deborah PM Symmons[7], John R Thompson[48], Wendy Thomson[7], Martin D Tobin[48], Mary E Travers[8], Clare Turnbull[16], Damjan Vukcevic[18], Louise V Wain[48], Mark Walker[49], Neil M Walker[17], Chris Wallace[17], Margaret Warren-Perry[16], Nicholas A Watkins[3,4], John Webster[50], Michael N Weedon[2], Anthony G Wilson[51], Matthew Woodburn[17], B Paul Wordsworth[52], Chris Yau[5], Allan H Young[27,53], Eleftheria Zeggini[1], Matthew A Brown[52,54], Paul R Burton[48], Mark J Caulfield[34], Alastair Compston[43], Martin Farrall[55], Stephen CL Gough[8,10,30], Alistair S Hall[6], Andrew T Hattersley[2,56], Adrian VS Hill[18], Christopher G Mathew[9], Marcus Pembrey[57], Jack Satsangi[21], Michael R Stratton[1,16], Jane Worthington[7], Matthew E Hurles[1], Audrey Duncanson[58], Willem H Ouwehand[1,3,4], Miles Parkes[12], Nazneen Rahman[16], John A Todd[17], Nilesh J Samani[11,59], Dominic P Kwiatkowski[1,18], Mark I McCarthy[8,18,60], Nick Craddock[29], Panos Deloukas[1], Peter Donnelly[5,18].

[1]The Wellcome Trust Sanger Institute, Wellcome Trust Genome Campus, Hinxton, Cambridge, CB10 1SA UK. [2]Genetics of Complex Traits, Peninsula College of Medicine and Dentistry University of Exeter, EX1 2LU, UK. [3]Department of Haematology, University of Cambridge, Long Road, Cambridge, CB2 0PT, UK. [4]National Health Service Blood and Transplant, Cambridge Centre, Long Road, Cambridge CB2 0PT, UK. [5]Department of Statistics, University of Oxford, 1 South Parks Road, Oxford, OX1 3TG, UK. [6]Multidisciplinary Cardiovascular Research Centre (MCRC), Leeds Institute of Genetics, Health and Therapeutics (LIGHT), University of Leeds, Leeds, LS2 9JT, UK. [7]arc Epidemiology Unit, Stopford Building, University of Manchester, Oxford Road, Manchester, M13 9PT, UK. [8]Oxford Centre for Diabetes, Endocrinology and Medicine, University of Oxford, Churchill Hospital, Oxford OX3 7LJ, UK. [9]Department of Medical and Molecular Genetics, King's College London School of Medicine, 8th Floor Guy's Tower, Guy's Hospital, London, SE1 9RT, UK. [10]Centre for Endocrinology, Diabetes and Metabolism, Institute of Biomedical Research, University of Birmingham, Birmingham, B15 2TT, UK. [11]Department of Cardiovascular Sciences, University of Leicester, Glenfield Hospital, Groby Road, Leicester LE3 9QP, UK. [12]IBD Genetics Research Group, Addenbrooke's Hospital, Cambridge, CB2 0QQ, UK. [13]University of Aberdeen, Institute of Medical Sciences, Foresterhill, Aberdeen AB25 2ZD, UK. [14]SGDP, The Institute of Psychiatry, King's College London, De Crespigny Park, Denmark Hill, London SE5 8AF, UK. [15]Clinical Pharmacology Unit, University of Cambridge, Addenbrookes Hospital, Hills Road, Cambridge CB2 2QQ, UK. [16]Section of Cancer Genetics, Institute of Cancer Research, 15 Cotswold Road, Sutton SM2 5NG, UK. [17]Juvenile Diabetes Research Foundation/Wellcome Trust Diabetes and Inflammation Laboratory, Department of Medical Genetics, Cambridge Institute for Medical Research, University of Cambridge, Wellcome Trust/MRC Building, Cambridge CB2 0XY, UK. [18]The Wellcome Trust Centre for Human Genetics, University of Oxford, Roosevelt Drive, Oxford OX3 7BN, UK. [19]Department of Psychiatry, University of Birmingham, National Centre for Mental Health, 25 Vincent Drive, Birmingham, B15 2FG, UK. [20]BHF Glasgow Cardiovascular Research Centre, University of Glasgow, 126 University Place, Glasgow, G12 8TA, UK. [21]Gastrointestinal Unit, Division of Medical Sciences, School of Molecular and Clinical Medicine, University of Edinburgh, Western General Hospital, Edinburgh EH4 2XU, UK. [22]Academic Unit of Genetic Medicine, University of Southampton, Southampton, UK. [23]Endoscopy Regional Training Unit, Torbay Hospital, Torbay TQ2 7AA, UK. [24]Academic Unit of Musculoskeletal Disease, University of Leeds, Chapel Allerton Hospital, Leeds, West Yorkshire LS7 4SA, UK. [25]MRC Centre for Causal Analyses in Translational Epidemiology, Department of Social Medicine, University of Bristol, Bristol, BS8 2BN, UK. [26]Department of Medical Genetics, Manchester Academic Health Science Centre (MAHSC), University of Manchester, Manchester M13 0JH, UK. [27]School of Neurology, Neurobiology and Psychiatry, Royal Victoria Infirmary, Queen Victoria Road, Newcastle upon Tyne, NE1 4LP, UK. [28]Institute for Digestive Diseases, University College London Hospitals Trust, London NW1 2BU, UK. [29]MRC Centre for Neuropsychiatric Genetics and Genomics, School of Medicine, Cardiff University, Heath Park, Cardiff, CF14 4XN, UK. [30]University Hospital Birmingham NHS Foundation Trust, Birmingham, B15 2TT, UK. [31]University of Oxford, Institute of Musculoskeletal Sciences, Botnar Research Centre, Oxford, OX3 7LD, UK. [32]Centre for Diabetes and Metabolic Medicine, Barts and The London, Royal London Hospital, Whitechapel, London, E1 1BB, UK. [33]Bone Research Group, Department of Medicine and Therapeutics, University of Aberdeen, Aberdeen, AB25 2ZD, UK. [34]Clinical Pharmacology and Barts and The London Genome

Nature Genetics: doi:10.1038/ng.2480

Centre, William Harvey Research Institute, Barts and The London School of Medicine and Dentistry, Queen Mary University of London, Charterhouse Square, London EC1M 6BQ, UK. [35]Institute of Cellular Medicine, Musculoskeletal Research Group, 4th Floor, Catherine Cookson Building, The Medical School, Framlington Place, Newcastle upon Tyne, NE2 4HH, UK. [36]Gastroenterology Unit, Radcliffe Infirmary, University of Oxford, Oxford, OX2 6HE, UK. [37]Centre National de Genotypage, 2, Rue Gaston Cremieux, Evry, Paris 91057, France. [38]Department of Gastroenterology & Hepatology, University of Newcastle upon Tyne, Royal Victoria Infirmary, Newcastle upon Tyne NE1 4LP, UK. [39]ALSPAC Laboratory, Department of Social Medicine, University of Bristol, BS8 2BN, UK. [40]Division of Nutritional Sciences, King's College London School of Biomedical and Health Sciences, London SE1 9NH, UK. [41]NIHR-Leeds Musculoskeletal Biomedical Research Unit, University of Leeds, Chapel Allerton Hospital, Leeds, West Yorkshire LS7 4SA, UK. [42]Department of General Internal Medicine, Ninewells Hospital and Medical School, Ninewells Avenue, Dundee DD1 9SY, UK. [43]Department of Clinical Neurosciences, University of Cambridge, Addenbrooke's Hospital, Hills Road, Cambridge, CB2 2QQ, UK. [44]Clinical and Academic Rheumatology, Kings College Hosptal National Health Service Foundation Trust, Denmark Hill, London SE5 9RS, UK. [45]University of Toronto, St. Michael's Hospital, 30 Bond Street, Toronto, Ontario M5B 1W8, Canada. [46]University of Bath, Claverdon, Norwood House, Room 5.11a Bath Somerset BA2 7AY, UK. [47]Division of Community Health Sciences, St George's, University of London, London SW17 0RE, UK. [48]Departments of Health Sciences and Genetics, University of Leicester, 217 Adrian Building, University Road, Leicester, LE1 7RH, UK. [49]Diabetes Research Group, School of Clinical Medical Sciences, Newcastle University, Framlington Place, Newcastle upon Tyne NE2 4HH, UK. [50]Medicine and Therapeutics, Aberdeen Royal Infirmary, Foresterhill, Aberdeen, Grampian AB9 2ZB, UK. [51]School of Medicine and Biomedical Sciences, University of Sheffield, Sheffield, S10 2JF, UK. [52]Nuffield Department of Orthopaedics, Rheumatology and Musculoskeletal Sciences, Nuffield Orthopaedic Centre, University of Oxford, Windmill Road, Headington, Oxford, OX3 7LD, UK. [53]UBC Institute of Mental Health, 430-5950 University Boulevard Vancouver, British Columbia, V6T 1Z3, Canada. [54]Diamantina Institute of Cancer, Immunology and Metabolic Medicine, Princess Alexandra Hospital, University of Queensland, Ipswich Road, Woolloongabba, Brisbane, Queensland, 4102, Australia. [55]Cardiovascular Medicine, University of Oxford, Wellcome Trust Centre for Human Genetics, Roosevelt Drive, Oxford OX3 7BN, UK. [56]Genetics of Diabetes, Peninsula College of Medicine and Dentistry, University of Exeter, Barrack Road, Exeter, EX2 5DW, UK. [57]Clinical and Molecular Genetics Unit, Institute of Child Health, University College London, 30 Guilford Street, London WC1N 1EH, UK. [58]The Wellcome Trust, Gibbs Building, 215 Euston Road, London NW1 2BE, UK. [59]Leicester NIHR Biomedical Research Unit in Cardiovascular Disease, Glenfield Hospital, Leicester, LE3 9QP, UK. [60]Oxford NIHR Biomedical Research Centre, Churchill Hospital, Oxford, OX3 7LJ, UK

Nature Genetics: doi:10.1038/ng.2480

References

1. Schunkert, H. et al. Large-scale association analysis identifies 13 new susceptibility loci for coronary artery disease. *Nat Genet* **43**, 333-8.
2. Iribarren, C. et al. Metabolic syndrome and early-onset coronary artery disease: is the whole greater than its parts? *J Am Coll Cardiol* **48**, 1800-7 (2006).
3. Go, A.S. et al. Statin and beta-blocker therapy and the initial presentation of coronary heart disease. *Ann Intern Med* **144**, 229-38 (2006).
4. Assimes, T.L. et al. Susceptibility locus for clinical and subclinical coronary artery disease at chromosome 9p21 in the multi-ethnic ADVANCE study. *Hum Mol Genet* **17**, 2320-8 (2008).
5. Taylor-Piliae, R.E. et al. Validation of a new brief physical activity survey among men and women aged 60-69 years. *Am J Epidemiol* **164**, 598-606 (2006).
6. Samani, N.J. et al. Large scale association analysis of novel genetic loci for coronary artery disease. *Arterioscler Thromb Vasc Biol* **29**, 774-80 (2009).
7. Arking, D.E. et al. A common genetic variant in the NOS1 regulator NOS1AP modulates cardiac repolarization. *Nat Genet* **38**, 644-51 (2006).
8. Samani, N.J. et al. Genomewide association analysis of coronary artery disease. *N Engl J Med* **357**, 443-53 (2007).
9. Inouye, M. et al. Metabonomic, transcriptomic, and genomic variation of a population cohort. *Mol Syst Biol* **6**, 441.
10. Shah, S.H. et al. Association of a peripheral blood metabolic profile with coronary artery disease and risk of subsequent cardiovascular events. *Circ Cardiovasc Genet* **3**, 207-14.
11. Nelis, M. et al. Genetic structure of Europeans: a view from the North-East. *PLoS One* **4**, e5472 (2009).
12. Invasive compared with non-invasive treatment in unstable coronary-artery disease: FRISC II prospective randomised multicentre study. FRagmin and Fast Revascularisation during InStability in Coronary artery disease Investigators. *Lancet* **354**, 708-15 (1999).
13. Renstrom, F. et al. Genetic predisposition to long-term nondiabetic deteriorations in glucose homeostasis: Ten-year follow-up of the GLACIER study. *Diabetes* **60**, 345-54.
14. Morris, A.D. et al. The diabetes audit and research in Tayside Scotland (DARTS) study: electronic record linkage to create a diabetes register. DARTS/MEMO Collaboration. *Bmj* **315**, 524-8 (1997).
15. Collins, R., Armitage, J., Parish, S., Sleight, P. & Peto, R. Effects of cholesterol-lowering with simvastatin on stroke and other major vascular events in 20536 people with cerebrovascular disease or other high-risk conditions. *Lancet* **363**, 757-67 (2004).
16. Anand, S.S. et al. Genetic variants associated with myocardial infarction risk factors in over 8000 individuals from five ethnic groups: The INTERHEART Genetics Study. *Circ Cardiovasc Genet* **2**, 16-25 (2009).
17. Chambers, J.C. et al. Common genetic variation near MC4R is associated with waist circumference and insulin resistance. *Nat Genet* **40**, 716-8 (2008).
18. Chambers, J.C. et al. Genome-wide association study identifies variants in TMPRSS6 associated with hemoglobin levels. *Nat Genet* **41**, 1170-2 (2009).
19. Kooner, J.S. et al. Genome-wide scan identifies variation in MLXIPL associated with plasma triglycerides. *Nat Genet* **40**, 149-51 (2008).
20. Ritsch, A. et al. Cholesteryl ester transfer protein and mortality in patients undergoing coronary angiography: the Ludwigshafen Risk and Cardiovascular Health study. *Circulation* **121**, 366-74.
21. Haenle, M.M. et al. Overweight, physical activity, tobacco and alcohol consumption in a cross-sectional random sample of German adults. *BMC Public Health* **6**, 233 (2006).
22. Stancakova, A. et al. Changes in insulin sensitivity and insulin release in relation to glycemia and glucose tolerance in 6,414 Finnish men. *Diabetes* **58**, 1212-21 (2009).
23. Evans, A. et al. MORGAM (an international pooling of cardiovascular cohorts). *Int J Epidemiol* **34**, 21-7 (2005).

Nature Genetics: doi:10.1038/ng.2480

24. Tunstall-Pedoe H, K.K., Tolonen H, Davidson M, Mendis S. *MONICA Monograph and Multimedia Sourcebook.*, (Geneva: World Health Organization, 2003).
25. McPherson, R. et al. A common allele on chromosome 9 associated with coronary heart disease. *Science* **316**, 1488-91 (2007).
26. Ingelsson, E., Hulthe, J. & Lind, L. Inflammatory markers in relation to insulin resistance and the metabolic syndrome. *Eur J Clin Invest* **38**, 502-9 (2008).
27. Schunkert, H. et al. Repeated replication and a prospective meta-analysis of the association between chromosome 9p21.3 and coronary artery disease. *Circulation* **117**, 1675-84 (2008).
28. Broadbent, H.M. et al. Susceptibility to coronary artery disease and diabetes is encoded by distinct, tightly linked SNPs in the ANRIL locus on chromosome 9p. *Hum Mol Genet* **17**, 806-14 (2008).
29. Saleheen, D. et al. The Pakistan Risk of Myocardial Infarction Study: a resource for the study of genetic, lifestyle and other determinants of myocardial infarction in South Asia. *Eur J Epidemiol* **24**, 329-38 (2009).
30. Hong, Y., Pedersen, N.L., Brismar, K. & de Faire, U. Genetic and environmental architecture of the features of the insulin-resistance syndrome. *Am J Hum Genet* **60**, 143-52 (1997).
31. Theodoraki, E.V. et al. Fibrinogen beta variants confer protection against coronary artery disease in a Greek case-control study. *BMC Med Genet* **11**, 28.
32. Zethelius, B., Byberg, L., Hales, C.N., Lithell, H. & Berne, C. Proinsulin and acute insulin response independently predict Type 2 diabetes mellitus in men--report from 27 years of follow-up study. *Diabetologia* **46**, 20-6 (2003).
33. Brouilette, S., Singh, R.K., Thompson, J.R., Goodall, A.H. & Samani, N.J. White cell telomere length and risk of premature myocardial infarction. *Arterioscler Thromb Vasc Biol* **23**, 842-6 (2003).
34. Hall, A.S. et al. A randomized, controlled trial of simvastatin versus rosuvastatin in patients with acute myocardial infarction: the Secondary Prevention of Acute Coronary Events--Reduction of Cholesterol to Key European Targets Trial. *Eur J Cardiovasc Prev Rehabil* **16**, 712-21 (2009).
35. Samani, N.J. et al. A genomewide linkage study of 1,933 families affected by premature coronary artery disease: The British Heart Foundation (BHF) Family Heart Study. *Am J Hum Genet* **77**, 1011-20 (2005).
36. Vaara, S. et al. Cohort Profile: The Corogene study. *Int J Epidemiol* **41**, 1265-71.
37. Nieminen, T. et al. The Finnish Cardiovascular Study (FINCAVAS): characterising patients with high risk of cardiovascular morbidity and mortality. *BMC Cardiovasc Disord* **6**, 9 (2006).
38. Thygesen, K. et al. Universal definition of myocardial infarction. *Circulation* **116**, 2634-53 (2007).
39. Ferreira, M.A. et al. Identification of IL6R and chromosome 11q13.5 as risk loci for asthma. *Lancet* **378**, 1006-14.
40. Dehghan, A. et al. Meta-analysis of genome-wide association studies in >80 000 subjects identifies multiple loci for C-reactive protein levels. *Circulation* **123**, 731-8.
41. Aulchenko, Y.S. et al. Loci influencing lipid levels and coronary heart disease risk in 16 European population cohorts. *Nat Genet* **41**, 47-55 (2009).
42. Teupser, D. et al. Genetic regulation of serum phytosterol levels and risk of coronary artery disease. *Circ Cardiovasc Genet* **3**, 331-9.
43. Teslovich, T.M. et al. Biological, clinical and population relevance of 95 loci for blood lipids. *Nature* **466**, 707-13.
44. Siednienko, J., Nowak, J., Moynagh, P.N. & Gorczyca, W.A. Nitric oxide affects IL-6 expression in human peripheral blood mononuclear cells involving cGMP-dependent modulation of NF-kappaB activity. *Cytokine* **54**, 282-8.
45. Bis, J.C. et al. Meta-analysis of genome-wide association studies from the CHARGE consortium identifies common variants associated with carotid intima media thickness and plaque. *Nat Genet* **43**, 940-7.
46. Franke, A. et al. Genome-wide meta-analysis increases to 71 the number of confirmed Crohn's disease susceptibility loci. *Nat Genet* **42**, 1118-25.

Nature Genetics: doi:10.1038/ng.2480

47. Bellenguez, C. et al. Genome-wide association study identifies a variant in HDAC9 associated with large vessel ischemic stroke. *Nat Genet* **44**, 328-33.
48. Loucif, Y., Methot, J., Tremblay, K., Brisson, D. & Gaudet, D. Contribution of adiponectin to the cardiometabolic risk of postmenopausal women with loss-of-function lipoprotein lipase gene mutations. *Menopause* **18**, 558-62.
49. Agirbasli, M., Sumerkan, M.C., Eren, F. & Agirbasli, D. The S447X variant of lipoprotein lipase gene is inversely associated with severity of coronary artery disease. *Heart Vessels* **26**, 457-63.

Nature Genetics: doi:10.1038/ng.2480
