# Supplementary material for: Causal effects for genetic variants of osteoprotegerin on the risk of acute myocardial infarction and coronary heart disease: A two-sample Mendelian randomization study
Source: Front Cardiovasc Med. 2023 Mar 7;10:1041231. doi: 10.3389/fcvm.2023.1041231 (PMC10028206; doi:10.3389/fcvm.2023.1041231)
Supplement: Supplementary file 2 [file Table_3.DOCX]

Table S 1 **Sensitivity analyses with complementary methods after removing rs1385492**

|  |  | Directional pleiotropy | | Cochran Q-test | |  |
| --- | --- | --- | --- | --- | --- | --- |
| Exposure | outcome | intercepts | p-value | Q-statistic | p | Steiger P |
| osteoprotegerin | AMI | 0.009 | 0.677 | 5.596 | 0.347 | 5.73*10^-83^ |
| osteoprotegerin | CHD | 0.014 | 0.4723 | 5.887 | 0.3173 | 1.17*10^-83^ |

AMI: acute myocardial infarction; CHD: coronary heart disease

**Table S2 MVMR analysis of genetic determined OPG (P < 5 × 10−8) with AMI or CHD**

|  |  | AMI | | | CHD | | |
| --- | --- | --- | --- | --- | --- | --- | --- |
| Exposure | Methods | β | SE | P | β | SE | P |
| OPG | MR Egger | -0.066441 | 0.096979 | 0.494094 | -0.057714 | 0.090267 | 0.523344 |
|  | WM | -0.029066 | 0.063871 | 0.0449059 | -0.126275 | 0.058819 | 0.031807 |
|  | IVW | -0.070865 | 0.082364 | 0.0389575 | -0.081339 | 0.076842 | 0.0289816 |
|  | SM | -0.154793 | 7.939388 | 0.984465 | -0.277611 | 10.11758 | 0.978138 |
|  | Weighted mode | -0.154793 | 8.293655 | 0.985128 | -0.277611 | 9.818741 | 0.977473 |
| Smoking | MR Egger | 0.498982 | 0.189245 | 0.00905 | 0.436653 | 0.177571 | 0.014808 |
|  | WM | 0.223016 | 0.087118 | 0.010469 | 0.271906 | 0.07657 | 0.000384 |
|  | IVW | 0.277531 | 0.129933 | 0.032683 | 0.261165 | 0.121625 | 0.03177 |
|  | SM | 3.483118 | 1719.446 | 0.998386 | 2.659192 | 1110.918 | 0.998093 |
|  | Weighted mode | 3.483118 | 1847.885 | 0.998498 | 2.659192 | 1055.817 | 0.997993 |
| LDL | MR Egger | 0.563615 | 0.094672 | <0.0001 | 0.630507 | 0.082789 | <0.0001 |
|  | WM | 0.573941 | 0.066333 | <0.0001 | 0.641885 | 0.056551 | <0.0001 |
|  | IVW | 0.476471 | 0.07311 | <0.0001 | 0.548151 | 0.064162 | <0.0001 |
|  | SM | 0.596464 | 14.1993 | 0.966536 | 0.63592 | 10.86488 | 0.953387 |
|  | Weighted mode | 0.596464 | 12.87309 | 0.963091 | 0.63592 | 11.19595 | 0.954763 |

AMI: acute myocardial infarction; CHD: coronary heart disease; OPG: osteoprotegerin; LDL: Low-Density Lipoprotein; WM: Weighted Median; IVW: Inverse variance weighted;

**Table S3 TSMR analysis of genetic determined smoking，LDL(P < 5 × 10−8) with OPG**

|  |  | OPG | | |
| --- | --- | --- | --- | --- |
| Exposure | Methods | β | SE | P |
| Smoking | MR Egger | 0.434665 | 0.248365 | 0.083887 |
|  | WM | -0.051243 | 0.066641 | 0.441929 |
|  | IVW | -0.043226 | 0.050192 | 0.389118 |
|  | SM | -0.106152 | 0.169356 | 0.532533 |
|  | Weighted mode | -0.067832 | 0.157621 | 0.668072 |
| LDL | MR Egger | -0.0099316 | 0.06047378 | 0.8697583 |
|  | WM | 0.00608615 | 0.04611487 | 0.8950016 |
|  | IVW | -0.0023731 | 0.03990807 | 0.9525818 |
|  | SM | -0.0426243 | 0.0998802 | 0.6701317 |
|  | Weighted mode | -0.0426243 | 0.04373038 | 0.3311766 |

OPG: osteoprotegerin; LDL: Low-Density Lipoprotein; WM: Weighted Median; IVW: Inverse variance weighted;
